# The genomic landscape of 2,023 colorectal cancers

**DOI:** 10.1038/s41586-024-07747-9

**Published:** 2024-08-07

**Authors:** Alex J. Cornish, Andreas J. Gruber, Ben Kinnersley, Daniel Chubb, Anna Frangou, Giulio Caravagna, Boris Noyvert, Eszter Lakatos, Henry M. Wood, Steve Thorn, Richard Culliford, Claudia Arnedo-Pac, Jacob Househam, William Cross, Amit Sud, Philip Law, Maire Ni Leathlobhair, Aliah Hawari, Connor Woolley, Kitty Sherwood, Nathalie Feeley, Güler Gül, Juan Fernandez-Tajes, Luis Zapata, Ludmil B. Alexandrov, Nirupa Murugaesu, Alona Sosinsky, Jonathan Mitchell, Nuria Lopez-Bigas, Philip Quirke, David N. Church, Ian P. M. Tomlinson, Andrea Sottoriva, Trevor A. Graham, David C. Wedge, Richard S. Houlston

**Affiliations:** 1 Division of Genetics and Epidemiology, Institute of Cancer Research, London, UK; 2 Department of Biology, University of Konstanz, Konstanz, Germany; 3 Manchester Cancer Research Centre, Division of Cancer Sciences, University of Manchester, Manchester, UK; 4 University College London Cancer Institute, London, UK; 5 Big Data Institute, Nuffield Department of Medicine, University of Oxford, Oxford, UK; 6 Max Planck Institute for Molecular Cell Biology and Genetics, Dresden, Germany; 7 Department of Mathematics and Geosciences, University of Trieste, Trieste, Italy; 8 Centre for Evolution and Cancer, Institute of Cancer Research, London, UK; 9 Cancer Research UK Centre and Centre for Computational Biology, Institute of Cancer and Genomic Sciences, University of Birmingham, Birmingham, UK; 10 Department of Mathematical Sciences, Chalmers University of Technology, Gothenburg, Sweden; 11 Pathology and Data Analytics, Leeds Institute of Medical Research at St James’s, University of Leeds, Leeds, UK; 12 Department of Oncology, University of Oxford, Oxford, UK; 13 Institute for Research in Biomedicine Barcelona, The Barcelona Institute of Science and Technology, Barcelona, Spain; 14 Centro de Investigación Biomédica en Red de Cáncer (CIBERONC), Barcelona, Spain; 15 Institució Catalana de Recerca i Estudis Avançats (ICREA), Barcelona, Spain; 16 Research Department of Pathology, University College London, UCL Cancer Institute, London, UK; 17 Trinity College, Dublin, Ireland; 18 Edinburgh Cancer Research, Institute of Genetics and Cancer, University of Edinburgh, Edinburgh, UK; 19 Department of Cellular and Molecular Medicine, UC San Diego, La Jolla, CA USA; 20 Department of Bioengineering, UC San Diego, La Jolla, CA USA; 21 Moores Cancer Center, UC San Diego, La Jolla, CA USA; 22 Genomics England, William Harvey Research Institute, Queen Mary University of London, London, UK; 23 Wellcome Centre for Human Genetics, University of Oxford, Oxford, UK; 24 Oxford NIHR Comprehensive Biomedical Research Centre, Oxford University Hospitals NHS Foundation Trust, Oxford, UK; 25 Computational Biology Research Centre, Human Technopole, Milan, Italy

**Keywords:** Cancer genomics, Genetics research, Colorectal cancer, Next-generation sequencing, Evolutionary genetics

## Abstract

Colorectal carcinoma (CRC) is a common cause of mortality^1^, but a comprehensive description of its genomic landscape is lacking^2–9^. Here we perform whole-genome sequencing of 2,023 CRC samples from participants in the UK 100,000 Genomes Project, thereby providing a highly detailed somatic mutational landscape of this cancer. Integrated analyses identify more than 250 putative CRC driver genes, many not previously implicated in CRC or other cancers, including several recurrent changes outside the coding genome. We extend the molecular pathways involved in CRC development, define four new common subgroups of microsatellite-stable CRC based on genomic features and show that these groups have independent prognostic associations. We also characterize several rare molecular CRC subgroups, some with potential clinical relevance, including cancers with both microsatellite and chromosomal instability. We demonstrate a spectrum of mutational profiles across the colorectum, which reflect aetiological differences. These include the role of *Escherichia* *coli*^*pks+*^ colibactin in rectal cancers^10^ and the importance of the SBS93 signature^11–13^, which suggests that diet or smoking is a risk factor. Immune-escape driver mutations^14^ are near-ubiquitous in hypermutant tumours and occur in about half of microsatellite-stable CRCs, often in the form of HLA copy number changes. Many driver mutations are actionable, including those associated with rare subgroups (for example, *BRCA1* and *IDH1*), highlighting the role of whole-genome sequencing in optimizing patient care.

## Abstract (web summary)

Whole-genome sequencing of more than 2,000 colorectal carcinoma samples provides a highly detailed view of the genomic landscape of this cancer and identifies new driver mutations.

## Main

CRC is the third most common malignancy worldwide^1^. CRC sequencing projects have been limited to a few hundred cases and/or based on whole exome or gene panel sequencing^2–9^. The full complement of genomic lesions and associations with clinical features have not been fully established. Patients with CRC (median age of 69 years at sampling, range 23–94 years, 59% male) were recruited by the Genomics England 100,000 Genomes Project (100kGP) as detailed in the Methods. Whole-genome sequencing (WGS) was performed on DNA from 2,023 flash-frozen tumour samples (100× depth) and paired blood samples (33× depth) (Methods and Supplementary Tables 1 and 2). Sequenced cancer samples were primary carcinomas (*n* = 1,898), CRC metastases (*n* = 122) or recurrences (*n* = 3). The clinicopathological and molecular features of each cancer are available in a Genomic Data Table accessible in the 100kGP Research Environment (https://www.genomicsengland.co.uk/research/research-environment).

## Mutational processes and driver genes

**Extended Data Fig. 1:**
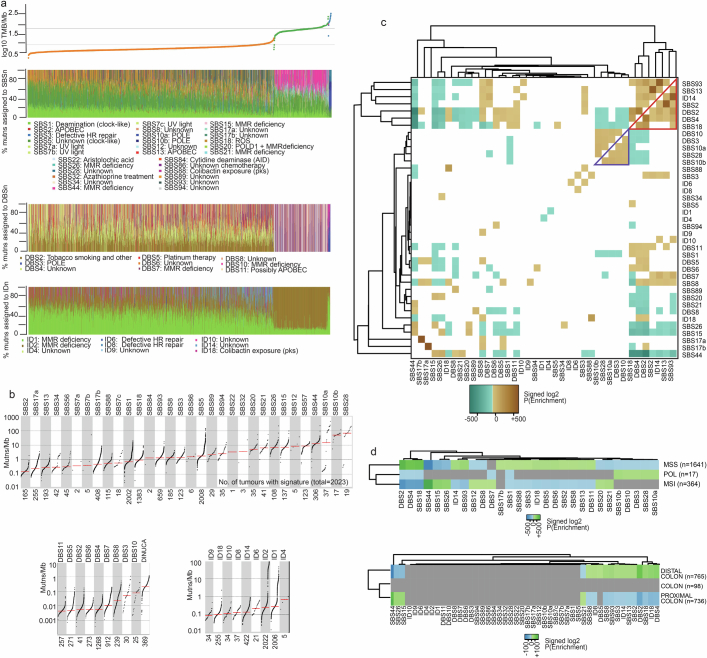
SBS, DBS and ID mutational signatures in each tumour. *(a) top-to-bottom: tumour mutation burden (TMB) per megabase (Mb) and mutational signature activity (% of mutations assigned) for SBS, DBS and ID mutations*. Tumour subtypes are: MSS primary (n = 1641, orange), MSI (n = 364, green) and POL (n = 17, blue). Tumours are first grouped according to their subtype and then ordered within each group from the lowest to the highest TMB. Common signatures included clock-like processes (e.g. SBS1, SBS5) and effects of specific underlying aetiologies (e.g. oxidative damage, SBS18). Signatures previously unreported in CRC included SBS89 and SBS94 (29 and 35 cancers, respectively; both unknown aetiology). Previously reported SBS30 (base excision repair), SBS40 (unknown aetiology) and ID7 (defective mismatch repair) were not found. *(b) Ascribed mutation burdens for each detected signature in all CRCs. (c) Pairwise associations between mutational signatures*. Clusters of co-occurrence, based on binary presence/absence, are highlighted by coloured triangles. Positive values (ochre) represent significant co-occurrence, whereas negative values (cyan) indicate relative exclusivity, with stronger associations in deeper shading (Bonferroni-corrected P values, Fisher’s exact test). Non-significant results are in white. Putative artefact signatures and signatures with no significant result (P_Bonf_ > 0.05) are not shown. Hierarchical clustering (Ward.D2, Euclidean distances) was performed on the rows and columns of the results matrix. Note negative associations between MSS- and MSI-specific signatures and positive associations between signatures with other likely shared aetiology (e.g. SBS17a/b). There were several novel associations of unknown origin. Notable relationships additional to those reported in the main article included an inverse association across all cancers between SBS44 (often MSI, dominated by C > T) and DBS2 (smoking, CC > NN) (P_Bonf_ = 1.4 x 10^−173^), DBS4 (GC > AA, TC > AA) (P_Bonf_ = 5.8 x 10^−137^) and SBS18 (C > A) (P_Bonf_ = 6.5 x1 0^−139^). A further cluster involved SBS10a/b, SBS28, DBS3 and DBS10 (driven by *POLE*). SBS3 tended to co-occur with ID6, ID8 and SBS88. *(d) Selected signatures showing significant differences among MSS primary, MSI and POL cancers (upper) or anatomical locations (lower)*. Associations are assessed as in (c), although co-occurrence is shown by green hues and mutual exclusivity in blue. MSI tumours were principally characterised by SBS44, and POL by SBS10a/b and SBS28. MSS cancers were enriched for SBS2, SBS8, SBS13, SBS18 and SBS93. SBS88, pks+ pathogenic E. coli exposure, was present in 115 (6%) cancers and ID18 (colibactin-derived) in 255 (13%). Note that these associations are uncorrected for covariables; multivariable analysis is shown in Supplementary Table 32. Further information is provided in Supplementary Result 1.

We initially classified CRCs into the three established subtypes: MSI (microsatellite instability-positive, mismatch repair deficient; *n* = 364); POL (DNA polymerase ε proofreading-deficient; *n* =18); and MSS (microsatellite-stable; *n* = 1,641). All except three of the metastasis samples were MSS (Methods). MSS cancers showed highly variable ploidy, whereas most MSI and POL cancers were near-diploid. Single-base substitution (SBS), doublet-base substitution (DBS) and small insertion–deletion (indel) mutational signature activities were broadly concordant with published work^12,15,16^, albeit with some important differences (Extended Data Fig. 1a,b and Supplementary Table 3).

We identified a potentially important role in CRC for SBS93 (mostly TTA>TCA and T>G), the fourth most common SBS signature (around 40% frequency in MSS primary tumours, but almost absent in MSI; mean activity 29%, range 13–82%). SBS93 has been associated with oesophageal and gastric cancers (https://cancer.sanger.ac.uk/signatures/sbs/sbs93/). Its presence in CRC has previously been noted^11–13^, but not accorded any significance. SBS93 showed transcriptional strand bias in our tumour samples (*P* < 0.001, Wilcoxon test), as it does in other cancers^12^, consistent with the action of transcription-coupled nucleotide excision repair on bulky DNA adducts caused by exogenous mutagens^17^. In MSS primary tumours, SBS93 co-occurred in a cluster with the signatures indel 14 (ID14; mostly insT in longer homopolymers and insC; *P*_Bonferroni_ = 1.6 × 10^–150^), SBS2 (TCN>TTN, *APOBEC*), SBS13 (TCN>TGN, *APOBEC*), SBS18 (C>A, oxidative damage), DBS2 (CC>AA, tobacco and aldehydes) and DBS4 (GC>AA, TC>AA) (Supplementary Table 3 and Extended Data Fig. 1c). Co-occurrence relationships for other signatures are described in Supplementary Result 1.

**Fig. 1:**
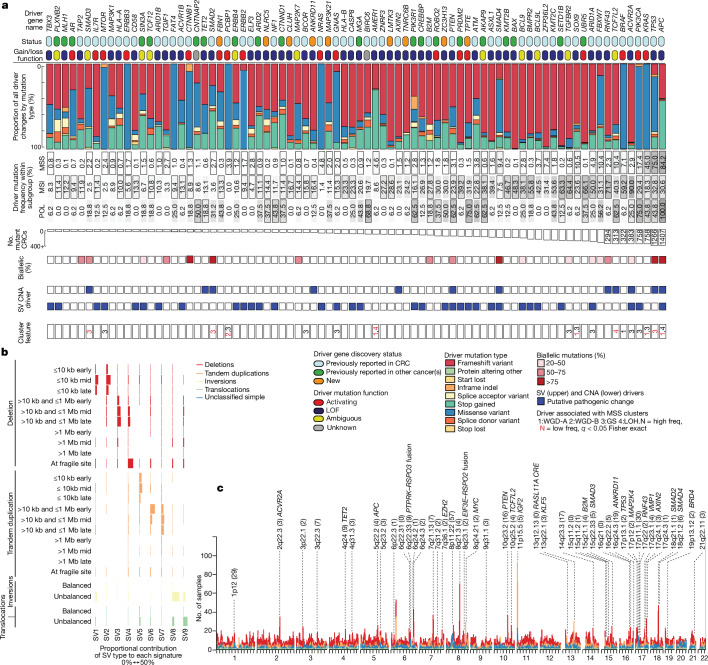
Driver genes and structural variants in CRC. **a**, The most commonly mutated driver genes based on separate analyses of SNVs, small indels and other base-level changes in the MSS primary, MSI, POL and MSS metastasis sets. Genes with the highest oncogenic mutation frequencies across the entire cohort are shown in rank order (most frequent on the right). For driver gene discovery, CRC drivers had previously been identified in any CRC cohort (or cohorts)^18^, whereas other cancer drivers had previously been identified only in non-CRC or multicancer cancer cohorts^2,18^. The remaining drivers were considered new. Mutation role (loss of function (LOF), activating, unknown or ambiguous) was assigned considering previous curation^18^ and predictions by this study. Conflicts or uncertainty were termed ambiguous. The percentage of tumours with a pathogenic mutation in the MSS primary (*n* = 1,521), MSI (*n* = 360) and POL (*n* = 16) cohorts are shown. Drivers identified in a specific cohort are in cells with a black border. Number mutated represents all tumours with a pathogenic mutation across all three cohorts. Also shown are: the percentage of tumours with biallelic mutations including LOH; status as a putative SV and/or focal CNA driver; and discriminant genes in the MSS primary cluster analysis. See also Extended Data Fig. 2. **b**, Nine SV signatures by underlying SV type in MSS primary, MSI and POL CRCs (*n* = 1,898). Horizontal coloured bars represent the contribution of each SV type to each signature. **c**, Significant simple SV hotspots identified in MSS primary CRCs (*n* = 1,354). Numbers of tumours with a SV at each genomic location (1 Mb regions) are coloured by the underlying type. Hotspots (excluding fragile sites) identified at *Q* < 0.05 (one-sided permutation test) are annotated with cytoband, the number of genes contained (in parentheses) and any candidate gene (Supplementary Table 10). Simple SVs comprise ≤2 individual rearrangements. Unclassified SVs could not be identified clearly as a deletion, tandem duplication, inversion or translocation.

**Extended Data Fig. 2:**
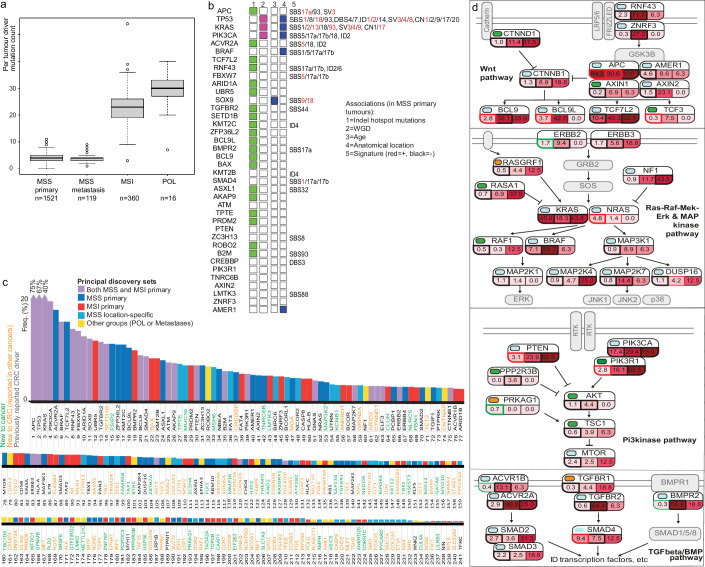
Driver mutations. *(a) Distribution of per-tumour driver mutation counts by CRC type*. Predicted pathogenic mutations from 193 driver genes (Supplementary Table 4) were included in the analysis which showed a highly significant difference (P = 2.6 x 10^−198^, two-sided Kruskal-Wallis). n, numbers of tumours in each of the four groups. *(b) Significant pairwise associations between the most frequently mutated driver genes and indel hotspot mutations, whole genome duplication, age, anatomical location and mutational signatures (Q<0.05). (c) Frequencies of 241 CRC SNV/indel driver gene mutations across all samples (including analysis of MSS primary cancers by anatomical location*, Supplementary Table 35*)*. The plot shows the sample sets in which the driver was discovered (colour of bar) and previous reports of the gene as a driver in CRC or other cancers (colour of gene name). The y-axis shows the proportion of cancers with a predicted pathogenic SNV or small indel mutation across the whole tumour set. In addition to these drivers, eight SV hotspots were denoted as likely drivers, involving genes *CDKAL1, BRD4, EZH2, IGF2, KCNQ1, MYC, UBE3A* and *VMP1* (Supplementary Table 35). *(d) Frequencies of putative driver mutations in four major signaling pathways, Wnt, Ras-Raf-Mek-Erk/MAP-kinase, Pi3 kinase and TGFβ/BMP*. Pathway information obtained from KEGG and TCGA. Key pathway genes not identified as CRC drivers by IntOGen are included in grey. Colour code for driver status is as per Fig. 1. Numbers refer to mutation frequency in that CRC subgroup (left-right: MSS, MSI, POL), with increasingly red shading for higher frequencies). Subgroups in which the gene was identified as a driver are shown with bold outline as per Fig. 1.

Driver gene identification at the base-pair level^18^ was performed separately in MSS primary, MSI (all primary), POL (all primary) and MSS metastasis CRCs to account for different background mutation rates (Methods). Overall, 193 putative CRC driver genes were detected using this strategy (Fig. 1a, Extended Data Fig. 2 and Supplementary Tables 4 and 5), with totals of 89, 96, 49 and 39, respectively in the four groups. In total, 57 drivers were identified in more than one group, leaving 136 present in a single group (44, 57, 27 and 8, respectively). Many of the candidate driver genes had not previously been reported in any cancer and others were new to CRC^2–9^.

Known CRC driver genes were generally mutated at reported frequencies. As expected given previous exome sequencing studies, all new MSS-specific coding drivers were low frequency (0.9–3.9%) and often with hotspot mutations (Supplementary Table 6 and Supplementary Result 2). By contrast, several of the new MSI drivers were relatively common, and were detectable in up to 50% of MSI tumours. Their identification was probably a reflection, in part, of the large sample size, but also of improved indel mutation calling compared with previous studies^7^. A prime example is the *BAX* tumour suppressor gene (TSG) (Supplementary Tables 6 and 7 and Supplementary Result 2).

Biological mechanisms highlighted by new drivers (Supplementary Table 4) included existing pathways, such as WNT and TGFβ–BMP, and less expected functions, such as RNA regulation (*ZC3H13* and *ZC3H4*) and transcriptional control (for example, the transcription factors *GTF2IRD2*, *MITF*, *MLF1*, *NCOA1*, *OLIG2*, *PRDM16*, *RUNX1*, *RUNX1T1*, *TCF12* and *TCF3*). Multiple members of the same family or pathway were frequently mutated. For example, several RAS–RAF–MEK–ERK and other MAP kinase pathway genes were MSS tumour drivers, including not only established ‘major drivers’ (*KRAS*, *NRAS* or *BRAF*) but also several ‘minor drivers’, including five MAP2 or MAP3 kinase genes, mostly involved in JUN kinase activation and signalling to MEK^19^ (Fig. 1a and Extended Data Fig. 2c,d). Other minor RAS pathway drivers included the RAS activator *RASGRF1* (RhoGEF domain mutations), *RAF1* (hotspot p.Ser257Leu) and the RAS suppressor *RASA1*, and an exemplar new MSI driver, the GTPase *RGS12* (Supplementary Result 3 and Supplementary Table 7). None of the minor RAS or MAP kinase drivers (Supplementary Table 4) was mutually exclusive with an established major RAS driver. Moreover, there was no association between the presence of major and minor RAS pathway drivers (odds ratio (OR) = 1.07, 95% confidence interval (CI) = 0.79–1.45, *P* = 0.73, two-tailed Fisher’s exact test, *n* = 1,521 MSS primary tumours). Finally, there was no evidence that minor RAS drivers could substitute for a major driver (mean minor RAS driver frequency of 0.12 in tumours with a major RAS driver compared with 0.13 without a major RAS driver, *P* = 0.58, two-tailed *t*-test, *n* = 1,521 MSS primary tumours). These data therefore suggest that the minor RAS and MAP kinase drivers act as modifiers of major RAS drivers and/or in a different branch of the MAP kinase pathway.

MSS tumours typically had four pathogenic driver mutations, whereas primary MSI and POL tumours had 23 and 30, respectively (*P* = 2.6 × 10^−198^, two-sided Kruskal–Wallis test; Extended Data Fig. 2a and Supplementary Table 8). Thirty genes were drivers in both MSS and MSI cancers, which emphasized the shared roles of WNT, RAS–RAF–MEK–ERK, PI3K, TGFβ–BMP, *TP53* and chromatin remodelling across CRC subtypes (Extended Data Fig. 2d). Other drivers were subtype-specific, yet indicated functional defects shared between MSS and MSI tumours, including genes that provided alternative ways of dysregulating the same pathways (Supplementary Tables 4–7). For example, TGFβ–BMP signalling was mostly inactivated by co-SMAD *SMAD4* mutations in MSS cancers, but by one or more indel receptor mutations (*TGFBR2*, *ACVR2A*, *BMPR2* and *ACVR1B*) in MSI cancers. Similarly, *BAX* mutations provided a biological alternative to *TP53* mutations in MSI tumours. Marked functional dissimilarities between MSS and MSI tumours were also found. For example, 12 MSI-specific drivers were annotated to immune functions compared with just 1 MSS-specific driver (detailed below). With the caveats of different sample sizes and mutational processes, the principal factors that underlie differences between MSS and MSI drivers were that the latter are subject to stronger selection for immune escape and can tolerate multiple and/or non-canonical changes in driver pathways (Supplementary Tables 4–6).

The identification of driver mutations remains subject to uncertainty, especially in hypermutant cancers and poor-quality samples. Of nearly 1,000 CRC drivers reported by other studies of primary CRC^2–9,20^, we only replicated 68 (7%) (Supplementary Table 9). Careful validation and functional assessment of our new putative drivers by other studies are similarly essential.

## Structural and copy number variants

Simple structural variants (SVs), inter-chromosomal translocations and complex SVs were identified using a consensus approach^16^ (Methods). Nine SV signatures were extracted across the cohort (Fig. 1b). SV8 (unbalanced inversions) and SV9 (unbalanced translocations) had not previously been identified in CRC.

**Extended Data Fig. 3:**
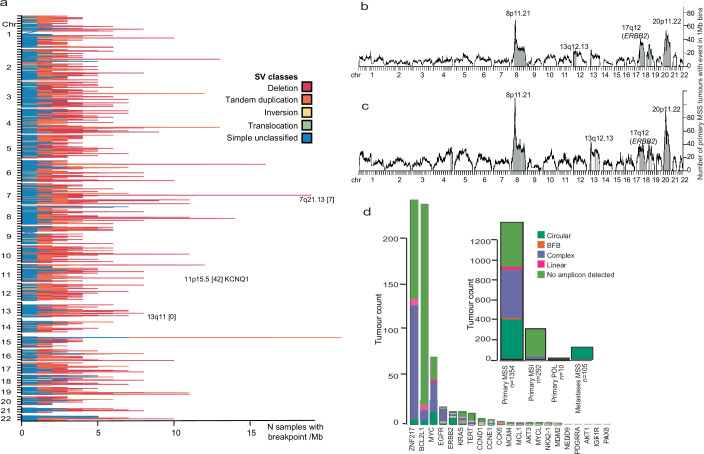
Somatic structural variation. *(a) Hotspots of simple structural variants (SVs) identified in MSI tumours (n* = *292)*. Coloured lines represent numbers of samples with a SV breakpoint of each class in 1Mb genome regions. Hotspots are annotated with their cytoband, the number of genes within their boundaries (in brackets) and any candidate gene. SVs at fragile sites are not included. *(b, c) Numbers of MSS primary tumours with (b) chromothripsis events and (c) unclassified complex SVs*. Regions enriched for chromothripsis and unclassified complex SV at a 5% FDR and greater than 5Mb in size are shaded. SVs at fragile sites are not included. *(d) Extrachromosomal DNA (ecDNA) across CRC subtypes and its contribution to common oncogene amplification*. The smaller chart shows the counts of tumours carrying at least one ecDNA amplicon across tumour subtypes (e.g. tumour counted as “Circular” if ≥ 1 circularised amplicon detected, otherwise “BFB” if ≥ 1 BFB amplicon detected until “No amp” where no valid amplicon detected). The larger chart shows ecDNA classification of commonly amplified oncogenes in MSS primary tumours. Classification was restricted to gene amplifications with a total copy number ≥ 5 in diploid tumours or ≥ 10 in tetraploid tumours (i.e. amplifications or “big gains”). See Supplementary Table 13.

Using simulation, 45 non-fragile SV hotspots (regarded as candidate driver changes) were found in MSS primary tumours and 3 in MSI tumours (*Q* < 0.05, one-sided permutation test; Fig. 1c, Extended Data Fig. 3a and Supplementary Table 10). Previously reported SV hotspots in MSS primary cancers included deletions (for example, *APC*, *PTEN*, *SMAD4* and *TP53*), amplifications (for example, *IGF2*, *MYC* and *RASL11A* regulatory element) and fusions (for example, *EIF3E–RSPO2* and *PTPRK–RSPO3*)^4,7,8,21^. Fusions involving the kinase domain of previously reported partner genes were identified in 0.4% and 4.1% of MSS and MSI cancers, respectively^22^ (8 *NTRK1*, 6 *BRAF*, 2 *ALK*, 1 *NTRK3* and 1 *RET*; Supplementary Table 11). Focal *TP53* deletions previously observed in osteosarcoma and prostate carcinoma^16^ were found in 2.4% of MSS primary tumours. A region on 17q23.1 containing *VMP1*, previously reported in breast cancer and pancreatic cancer^23,24^, was deleted in 1.2% of MSS primary tumours. Recurrent intronic deletions at 19p13.12 included a regulatory element interacting with the *BRD4* promoter^25^. *TET2* (0.8%) was a potential target of previously unknown 4q24 rearrangements, given its driver status in our POL cancers and a role in haematological malignancies^26^. *EZH2* was a credible target of a newly identified 7q31.2 deletion, given that low *EZH2* expression is associated with poor CRC prognosis^27^. In MSI cancers, we confirmed recurrent 11p15.1 deletions that encompass the MSI driver *CDKN1C*^28^, and six new SV hotspots. In MSS primary cancers, there was enrichment of complex SVs at locations with arm-level copy number alterations (CNAs), which indicated a common causal origin (Supplementary Table 12).

We analysed extrachromosomal DNA (ecDNA)^29^ to distinguish as far as possible truly circular ecDNA molecules from those characterized by breakage–fusion–bridge (BFB) cycles. ecDNA content differed by CRC type, with 28% (380 out of 1,354) of MSS primary tumours containing ≥1 predicted circular ecDNA compared with 1.4% (4 out of 292) MSI, 0% (0 out of 10) POL and 36% (38 out of 105) metastatic MSS tumours (*P* < 0.001, MSS primary compared with MSI, two-sided Kruskal–Wallis test; Extended Data Fig. 3d and Supplementary Table 13). MSS primary tumours with ecDNA were more likely to exhibit chromothripsis (*P* = 1.09 × 10^–12^, OR = 2.43, two-sided Fisher’s exact test), a result consistent with previous reports^30^. In MSS primary tumours, only 5% (34 out of 665) of oncogene amplifications (total copy number ≥ 5 in diploid tumours, ≥10 in tetraploid tumours) mapped to circular ecDNA. However, circular DNA was implicated in 14 out of 74 amplifications at *MYC* and 8 out of 15 at *ERBB2*. Our findings suggest that oncogene amplification through circularized ecDNA in CRC has only a modest role compared with other cancer types.

**Extended Data Fig. 4:**
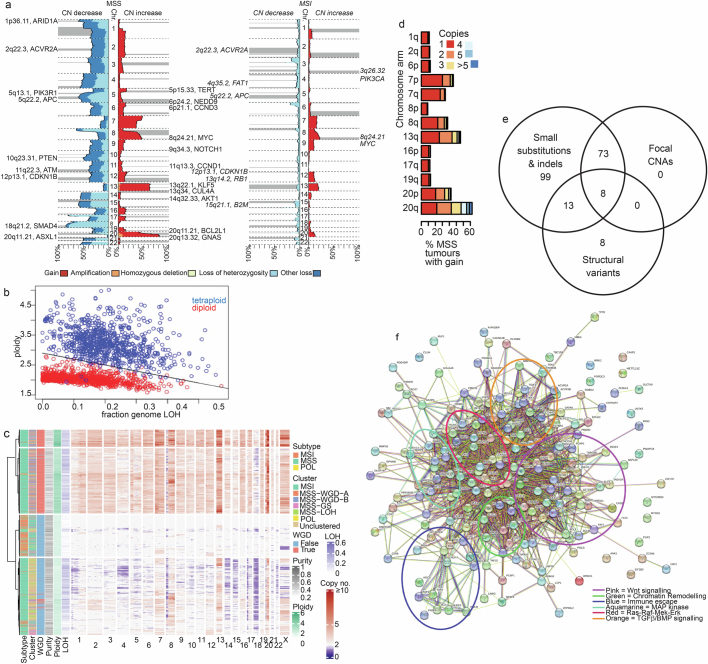
CNAs, SVs, WGD and pathways of tumorigenesis. *(a) CNA summary in MSS primary and MSI tumours*. Genome-wide frequencies of CNA in MSS primary (*n* = 1,354) and MSI (*n* = 292) tumours are shown. Focal amplifications and deletions reported by GISTIC analysis are shown as grey bars, and annotated with a cytoband and likely candidate gene where identified. Black dashed lines represent chromosome boundaries. *(b) Classification of all tumours into diploid and tetraploid (genome-doubled). (c) Hierarchical clustering of all tumours based only on copy number states identifies WGD/non-WGD split (column 2)*. CNA-based clustering identified a division based on WGD, with features highly reminiscent of the iCMS2/3 division identified by Joanito et al^139^ using single cell transcriptomics. *(d) Frequency of copy number gain in MSS primary tumours by chromosome arm. (e) Numbers of driver genes identified in the three main classes (SNV/indel, SV and focal CNA)*. Putative SV and focal CNA drivers must be (i) at a site significantly over-represented above background levels, and (ii) annotated to either a known SNV/indel driver or a single gene (i.e. there is only a single coding gene in the SV hotspot or focal CNA region). SNV/indel drivers identified in MSS cancers in a specific anatomical region of the colorectum are not shown (see Supplementary Tables 35 & 36). SV and CNA changes at fragile sites are excluded. CNAs in particular and SVs are likely to include some second hits at tumour suppressor genes (Supplementary Table 18). The following genes are annotated as putative CNA drivers based on focal changes, including focal or minimal overlapping regions of change*: ACVR1B, ACVR2A, AKT1, ANK1, APC, ARID1A, ARID1B, ARID2, ASXL1, ATM, AXIN1, B2M, BCL9, BCL9L, BMPR2, CASP8, CCND3, CD58, CDK12, CDKN1B, CHD2, CREBBP, CUL4A, DUSP16, EIF2B3, ELF3, EPHA3, ERBB2, ERBB4, FHIT, FKBP9, FOXP1, FSIP2, FUS, GNAS, GOLGA5, GPNMB, IDH1, IL7R, IRF1, KLF5, LCP1, MGA, MITF, MLF1, MTOR, MYH11, NBEA, NEDD9, NF1, NRAS, PAN3, PDE4DIP, PIK3CA, PIK3R1, PLK1, PLXNB2, POLE, POLG, POPDC3, PRDM2, PRKAG1, PRKCB, PTEN, PTPN11, PWWP2A, RASGRF1, RB1, ROBO2, SAP130, SETD1B, SIN3A, SMAD4, TBX3, TCF3, TFRC, THEMIS, TPTE, USP36, ZBTB7A and ZC3H13*. The following genes are annotated as putative SV drivers based on hotspots: *ACVR2A, ANK1, ANKRD11, APC, AXIN2, B2M, BRD4, CD58, CDKAL1, CDKN1C, CTNNB1, EZH2, IGF2, KCNQ1, KLF5, MAP2K4, MMP16, MYC, PTEN, RNF43, SMAD2, SMAD3, SMAD4, STAG1, TCF7L2, TET2, TP53, UBE3A* and *VMP1. (f) Molecular and functional connections between CRC driver genes from (e)*. Connections are derived from STRING. Gene annotation to the six pathways or “other pathway” was performed manually. Note that this analysis weights all driver genes equally.

Overall, 1,765 (87%) CRC samples passed quality control filters for CNA analysis (Methods and Extended Data Fig. 4a–d). The median CNA burden was 36 (range of 0–378) and the median estimated ploidy was 2.26 (range of 1.43–6.41). CNAs were uncommon in MSI and POL cancers, as expected. Whole-genome duplication (WGD)^31^ was identified in 45.0%, 5.8% and 10.0% of MSS primary, MSI and POL cancers, respectively. Within the MSS primary group, WGD most strongly co-occurred with *TP53* mutation^32^ and chromosome 13q gain, and with the absence of *KRAS* and *PIK3CA* mutations (*P* < 0.001, Fisher’s exact test). We found six CNA signatures (Supplementary Table 14), of which CN17 (*n* = 260, tandem duplication and HRD))^33^ had not previously been reported in CRC. All the identified signatures, except CN1 (diploidy), were enriched in MSS tumours. We found all previously reported, recurrent arm-level CNAs and whole chromosome changes (that is, events >50% of the total arm size)^7,31^ (Supplementary Table 15). Arm-level increased copy number typically involved single-copy or double-copy gains, with the exception of 20q, which gained four or more copies in 18% of MSS primary cancers (Extended Data Fig. 4d).

In total, 16 arm-level gains and 13 deletions were above background frequencies in MSS primary cancers, and we regarded these as candidate driver changes (Supplementary Table 15). Although MSI and POL cancers were mostly near-diploid, 17 arm-level CNAs (for example, gains of 7, 9, 12q and 14q and losses of 21q) were present in MSI cancers at levels above background. We identified a set of focal CNAs ≤3 Mb (Supplementary Table 16), and mapped minimal common regions shared between larger CNAs^34^. Previously reported focal CNAs in MSS primary cancers included single-copy and double-copy gains involving *CCND1*, *ERBB2*, *MYC* and *KLF5*, and deletions of *ARID1A*, *SMAD4* and *APC*^7,31^ (Supplementary Table 17). Although 5p15.33 (*TERT*) amplification was detected in 13 MSS cancers, we found no association with telomere length (TelomereHunter *P* = 0.78, Telomerecat *P* = 0.51, two-sided Kruskal–Wallis test)^35^. The following new focal CNAs were identified: 5q13.1 deletions (29%; *PIK3R1*); 15q11.2 deletions (42%; containing the lncRNA *PWRN1*, a tumour suppressor in gastric cancer^2^); and amplification at 6p21.1 (28%) and 6p25.3 (25%), which may target *CCND3* and *NEDD9*, respectively, genes that we also identified as putative drivers (Supplementary Table 4). There was shared causal overlap between CNAs and SVs, especially on chromosomes 8, 17, 18 and 20 (Extended Data Fig. 3b,c and Supplementary Result 4).

## Combined analysis of putative drivers

By combining small substitutions and indels, SVs and focal CNAs, we identified 201 putative driver genes (Extended Data Fig. 4e). Most candidate SV target genes were annotated to the locations of drivers found in the small-scale mutation analysis. About 7% of driver genes principally affected by indels and single nucleotide variants (SNVs) were also mutated by SVs, the latter typically constituting 1–4% of all mutations. The overlap between the sets of drivers affected by both small-scale mutations and CNAs was also strong, in part owing to second hits at TSGs. Evidence of two hits (Supplementary Table 18) was found for up to 90% of ‘classical’ tumour suppressor mutations (for example, *APC*, *SMAD4* and *TP53*), 75% of immune-escape drivers and 50% of the new RAS–RAF–MEK–ERK–MAP kinase drivers. However, the median second-hit rate across drivers was only 10%, and most new drivers did not adhere to a classical two-hit TSG model (albeit some were probably oncogenes). Almost no known or putative oncogenes showed clear evidence of second hits by amplification.

Pathway analysis of the putative CRC drivers using EnrichR^36^ identified many gene sets strongly associated with tumorigenesis and/or CRC pathogenesis (Supplementary Table 19). Almost all CRCs had changes in WNT, and most had changes in TGFβ–BMP, ERRB–RAS–RAF–MEK–ERK and p53 (Extended Data Figs. 2 and 4f). Other pathways involved less common drivers, including wider MAP kinase, NOTCH, chromatin regulation and transcriptional control (Supplementary Table 19). We found only limited evidence of new driver genes directly involved in DNA repair or hypermutation. Many tumour drivers or other molecular features were potentially clinically actionable (Supplementary Result 5 and Supplementary Tables 20–22).

Several signatures co-occurred with specific driver mutations (Extended Data Fig. 2b). In some cases, shared over-representation in MSS, MSI or POL cancers was the probable cause. Other pairwise relationships probably causally linked to each other included those between *TP53* and multiple copy number signatures, and between *ATM* and SV1.

## Finding common and rare CRC subgroups

**Fig. 2:**
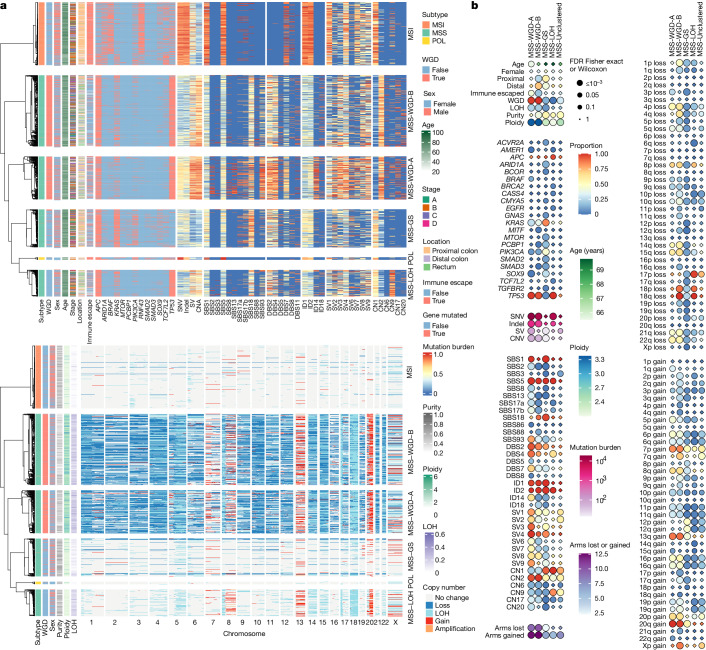
Identification of MSS primary CRC molecular subgroups by cluster analysis. **a**, Heatmap of the six clusters identified by consensus clustering for a subset of variables that showed a significant difference (false discovery rate (FDR) < 0.05) between the MSS clusters. The single cluster analysis is split into two parts for better visualization. Top, subtype (MSS primary, MSI and POL), WGD status, age at sampling, sex, Dukes stage, site, immune-escape status, genes, mutation burdens and signatures. Bottom, subtype, WGD status, purity, ploidy, fraction LOH and copy number states. Values for mutation burdens (SNV, indel, SV, CNA) and signatures (SBS, DBS, ID, SV and CN) are ranked and scaled to lie between 0 and 1. Driver gene mutations are shown by gene name. Chromosome arm-level changes are shown by 1–22 and X. **b**, Summary of significant and other selected associations between molecular features and MSS primary clusters relative to the entire MSS primary group. Circle size shows FDR, diamonds indicate non-significance (FDR > 0.05). For categorical variables measured as the proportion of tumours (for example, signature presence, immune escape), a heatmap scale between 0 and 1 is used. Quantitative variables each have a bespoke scale, as shown. Full data are shown in Supplementary Table 23. No significant difference between clusters (FDR > 0.05) was found for many variables, mostly those with a low frequency in MSS primary tumours. Notable moderate-frequency molecular variables without a significant association with cluster group included signatures DBS6 and SV5 and driver mutations in *FBXW7*, *SMAD4* and *PTEN*. There was also no significant association with microbiome diversity or prevalence of the top 20 bacterial genera.

**Extended Data Fig. 5:**
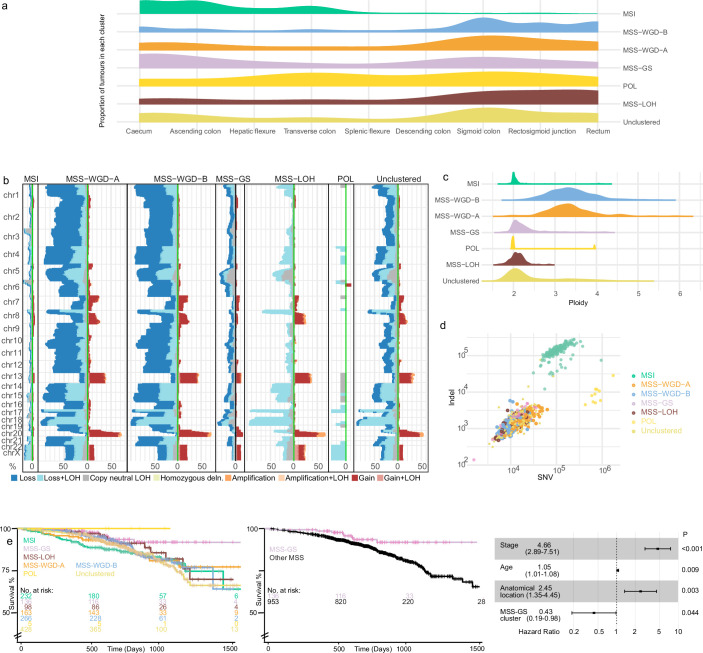
Clinicopathological and molecular features of the four MSS clusters in comparison with MSI and POL cancers. *(a) Anatomical sub-divisions of the colorectum (see* Fig. 4*)*. Note that numbers of CRCs in the splenic flexure and descending colon are generally relatively low compared with other regions*. (b) Copy number changes and LOH. (c) Ploidy. (d) SNV and indel burdens*. Note the lack of obvious structure within the MSS sub-group centroid. *(e) Survival*. Left, Kaplan-Meier plot showing overall survival of patients with tumours in the four MSS clusters with unclustered MSS, MSI and POL also shown. Median follow-up was 755 days. The failure to show the established association between MSI and good prognosis could be accounted for by the higher age and stage of the MSI patients, together with the non-availability of cancer-specific measures of survival. In analysis uncorrected for stage, age, location and other clinicopathological variables, logrankP = 0.16. Centre, Kaplan-Meier plot showing overall survival of MSS-GS cancer patients versus all other MSS cancers. Median follow-up was 754 days. In analysis uncorrected for stage, age, location and other clinicopathological variables, logrankP = 0.019. Right, multivariable analysis, showing that MSS-GS patients had significantly longer overall survival (HR = 0.43, P = 0.044) than the other MSS clusters in a CoxPH model including age, stage (C,D v A,B (reference)), and location (proximal colorectum v distal colorectum (reference)). Sex was not significantly associated with survival. In the forest plot, the boxes represent point estimates and the horizontal lines delimit 95% confidence intervals. No. at risk, number of patients entering the study at time 0, or for subsequent times, number who had not suffered an event or been censored during the previous time period.

To search for molecular subgroups of CRC based on genomic features, hierarchical clustering was performed using 304 molecular and clinical variables (Methods). Based on cancers with available CNA data, we found six stable clusters of 1,000 primary, treatment-naive tumours: MSI; POL; and four MSS clusters. We denoted the MSS clusters as WGD-A (24% of primary treatment-naive MSS), WGD-B (40%), genome stable (GS; 21%) and loss of heterozygosity (LOH; 15%). WGD frequencies in the MSS clusters were 97%, 99%, 14% and 0%, respectively (Figs. 1a and 2, Extended Data Fig. 5 and Supplementary Table 23). SNV and indel burdens of all MSS clusters were distinct from MSI and POL tumours. Both WGD clusters showed hallmarks of chromosomal instability (CIN). Specifically, they showed higher numbers of SV and CNA events, higher LOH and increased numbers of events attributed to copy number signatures CN6 (chromothripsis) and CN17 (arm-level LOH followed by two genome doubling events). Large fractions of these tumours had whole chromosome or arm-level losses (mean number of arms lost per tumour of 9.8).

MSS-WGD-A tumours had higher SNV and indel burdens and markedly higher numbers of events attributed to SBS93, ID14, DBS7 and SV signatures 1, 2, 3, 6, 7 and 9 (Supplementary Table 23). They also had increased frequencies of *BRAF* mutations, which were also strongly associated with MSI cancers. The second WGD cluster (MSS-WGD-B) was the largest, and might be regarded as ‘canonical’ MSS cancers. It was enriched relative to other cancers for distal location, SBS18 and the *E.* *coli*^*pks*+^ signatures SBS88 and ID18, although not for any specific driver mutation (except the rare driver *MITF*).

MSS-GS cancers showed few events associated with CIN (that is, predicted near-diploid karyotype, low levels of LOH, SVs, CNAs and arm-level losses (mean number of arms lost per tumour of 2.3). This cluster had the fewest *TP53* mutations (6%), a result consistent with a role for p53 in preventing multiple types of CIN, but the largest fractions of *KRAS* mutations (83%) and SBS18 activity (97%). The remaining cluster, MSS-LOH, showed an unusual form of CIN characterized by focal and arm-level LOH (and hence high CN9 activity), with intermediate SV, CNA and LOH burdens, and low SNV and indel burdens. In some respects, MSS-GS cancers resembled MSI cancers with respect to proximal location, near-diploid genomes and shared driver genes such as *TGFBR2*, *ACVR2A* and *ARID1A* (Fig. 2a), but there was no increased mutation burden (Extended Data Fig. 5). Patients with MSS-GS cancer had longer overall survival than other MSS cancers, and this cluster was an independent better prognostic factor, alongside worse prognosis associated with higher stage, greater age and proximal location in multivariable survival analysis of the entire patient set (Extended Data Fig. 5e and Supplementary Result 6).

**Extended Data Fig. 6:**
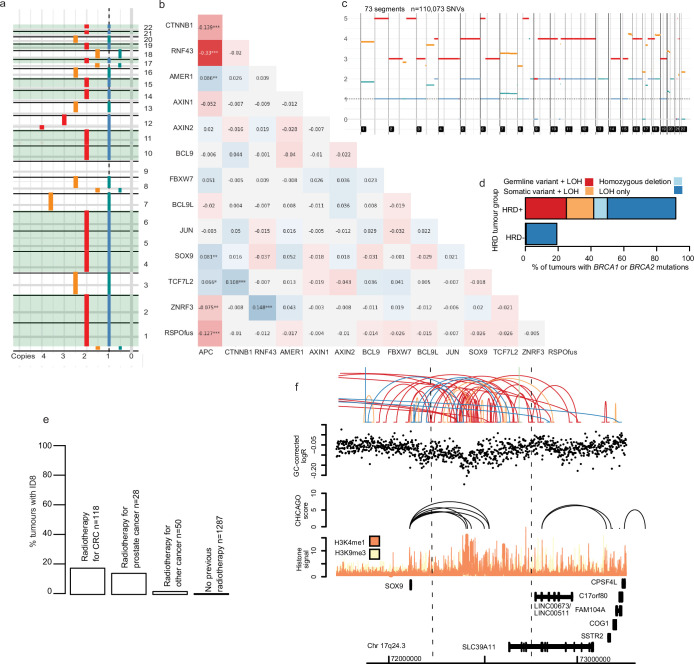
Rare molecular sub-groups and non-coding driver SVs. *(a) Representative copy number analysis of a cancer with sub-clonal SMAD4 (chr18q21.2) mutation*. The Battenberg output shows copy number along the genome from chromosome 1 to 22. Red bars indicate total copy number, orange bars sub-clonal copy number states and blue bars minor allele copy number. Integrating these data with SNV data shows the most parsimonious explanation to be that chromosome 18 has sub-clonal (average copy number ~0.5) loss, by clonal deletion of one homologue and the co-existence of two sub-clones of similar prevalence, one with deletion of the other homologue and the other with a loss of function *SMAD4* mutation. The presence of multiple other sub-clonal copy number changes in this tumour supports this view. *(b) Co-occurrence of Wnt pathway driver mutations in MSS primary tumours*. Pairwise comparison is by logistic regression, using co-variables of TMB, age, sex and location. The pairwise effect size β (co-occurrence >1 (blue), exclusivity <1 (red)) is shown in each square. Uncorrected two-sided *P*-values for the pairwise association are indicated as * <0.05, ** <0.01, *** <0.001. Note the co-occurrence of *CTNNB1* and *TCF7L2*, which is also present in MSI tumours (β = 0.26, P<0.001). *(c) Representative copy number analysis of an MSI cancer with WGD and chromosomal instability (CIN)*. The Battenberg output shows a grossly rearranged, polyploid genome, placing this cancer amongst the most altered of the MSS group. It contrasts sharply with the near-unaltered karyotypes of most other MSI cancers. *(d) Mutation status of BRCA1/2 in tumours with and without predicted homologous recombination deficiency (HRD) based on HRDetect (probability threshold 0.7)*. Germline or somatic *BRCA1/2* variants defined as moderate or high impact by Variant Effect Predictor (VEP) and/or reported as pathogenic or likely pathogenic by ClinVar (v1.20) were included in the analysis, together with CNAs. *(e) Proportion of cancers showing ID8 activity in patients who had received radiotherapy for treatment of their CRC or a different cancer prior to the CRC. (f) Multiple simple structural variants (SVs) identified at 17q24.3 overlapping lncRNAs and a regulatory element that interacts with the SOX9 promoter*. Data from MSS primary cancers (n = 1,354) are shown. Top track arcs represent simple SVs; second track shows mean GC-corrected log ratio between tumour and normal read coverage (logRR) as computed by Battenberg – higher and lower values indicate tendencies for copy number gains and losses respectively amongst the included tumours; third track shows chromosomal interactions identified in HT29 cells using promoter capture Hi-C; fourth track shows histone mark signals; and bottom track shows the locations of coding genes in the region and lncRNA LINC00673/LINC00511. Vertical lines represent hotspot start and end positions.

Rare cancer subgroups can also provide important insights into tumorigenesis, as exemplified by *POLE* driver mutations^7^. These occur in only 1–2% of CRCs but are associated with an exceptionally high mutational burden and good prognosis^37^. Our patient sample size provided an opportunity to identify or characterize other less common molecular subgroups of CRC (Extended Data Fig. 6 and Supplementary Result 7). We focused on five such rare subgroups: (1) subclonal driver mutations, notably parallel evolution of *SMAD4* mutations and 18q deletions (Extended Data Fig. 6a and Supplementary Table 24); (2) activating *CTNNB1* driver mutations that show complex co-occurrence relationships with other WNT drivers and almost all undergo loss of the wild-type allele, despite being dominant oncogenec alleles (Extended Data Fig. 6b and Supplementary Table 18); (3) MSI cancers with highly chromosomally unstable genomes (Extended Data Fig. 6c); (4) *BRCA1* and *BRCA2* mutant cancers and their associated, potentially targetable HRD (Extended Data Fig. 6d); and (5) patients who had received previous radiotherapy for prostate cancer, a risk factor for CRC^38^, showing the absence in most cases of radiotherapy-associated signature ID8 (Extended Data Fig. 6e).

## Immune editing and escape

**Fig. 3:**
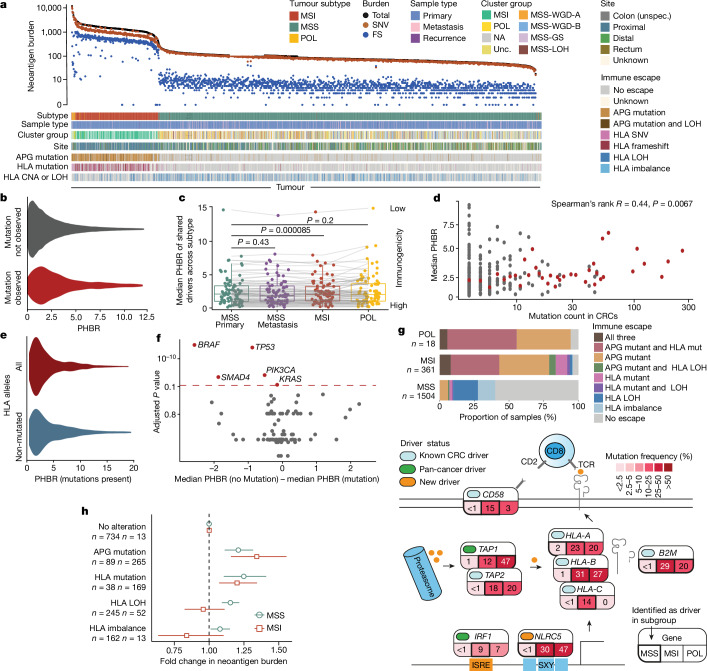
Immune landscape of CRC. **a**, Neoantigen burdens and immune-escape mutations. Bars show antigen-presenting or antigen-processing gene (APG) and HLA alterations in each cancer. FS, frameshift; unspec., unspecified; unc, unclassified. **b**, PHBR of all non-observed mutations in all cancers (*n* = 478,106 mutations) compared with observed mutations (*n* = 3,211 mutations). *P* = 6 × 10^–56^. **c**, Median PHBR of driver mutations (*n* = 80) shared between CRC subtypes, computed separately for cancers of each subtype. Lines connect PHBR values of the same mutation across subtypes. **d**, Median PHBR of driver mutations across the entire CRC cohort by mutation count. Grey dots represent individual mutations, red dots show the median for mutations at the same frequency. **e**, The influence of HLA alterations on PHBR. Values for each driver in each patient with a HLA mutation using the full set of patient-specific HLA alleles (red) are compared with values computed from a reduced, non-mutated set (blue). *P* = 2 × 10^–11^. **f**, Median PHBR difference of non-mutated and mutated driver gene changes within patients. Each dot denotes a driver. Genes with significant difference (*P*_Bonferroni_ < 0.1) are highlighted in red. **g**, Top, somatic mutations in components of the APG pathway by CRC subtype. Bottom, frequencies of cancers with mutations in specific APGs or HLA. A total of 140 cancers were excluded from the analysis owing to incompatible HLA types. **h**, Associations between immune-escape-associated somatic mutations and neoantigen burden. Multivariable regression analysis was performed in 1,412 MSS primary and 309 MSI cancers, using no HLA or APG alteration as the baseline. Circles or squares show odds ratio (OR) point estimates and whiskers show 95% CIs. Numbers of cancers with each type of alteration are shown (tumours can be present in more than one alteration group). Throughout, unless otherwise stated, two-sided Wilcoxon tests were used, and for box plots, the centre line shows the median, the box limits show upper and lower quartiles, and the whiskers show 1.5× inter-quartile range.

**Extended Data Fig. 7:**
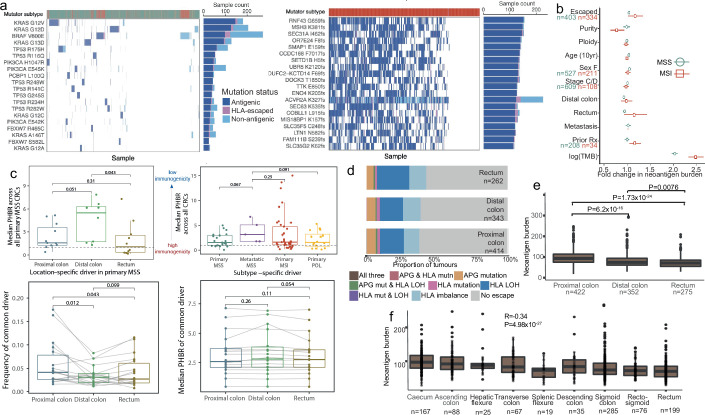
Driver mutation immunogenicity and immune escape. *(a) Heatmap and frequency chart of the 20 most common antigenic SNV and frameshift mutations*. Mutations are shown in order of decreasing frequency across the CRC set. Colours show antigenic mutations (dark blue), escaped antigenicity through HLA alteration (purple), or non-antigenic mutations (light blue). The molecular subtype of each cancer is shown above the heatmap (green: MSS, red: MSI, yellow: POL). Among recurrent non-synonymous mutations, *KRAS* G12V was most antigenic, predicted to bind patient-specific HLA molecules in 80% (146/181) of cancers. *KRAS* G12D and G13D were also frequently predicted to be antigenic, whereas the rarer *KRAS* mutations G12C, A146T and G12A were less so. *BRAF* V600E was predicted to be antigenic in only 36% (98/272) of cancers, as the HLA alleles binding the resulting epitope were either uncommon or, in 20% of cancers with predicted binding, underwent somatic loss. The most common peptide-changing frameshift mutations were principally found in MSI cancers, at a frequency of >40% (and are shown in these cancers only*)*. Frameshift mutations produced a neoantigen in >95% of cases, although the most frequent frameshift in MSS cancers, *APC* E1309fs, had low predicted antigenicity (30%, 14/47 cases). For the 20 most frequent non-synonymous changes, the observed mutation frequency and predicted antigenic frequency were inversely related (*P* = 0.042, two-sided Pearson correlation test). There was no equivalent association for the 20 most frequent frameshift changes (*P* = 0.32), plausibly reflecting their almost universally high immunogenicity. *(b) Dependency of neoantigen burden on immune escape, TMB and other clincopathological and molecular variables separately* in 1,450 MSS and 350 MSI cancers *in multivariable regression models*. Green circles and red squares represent odds ratios for each variable respectively, with whiskers showing 95% confidence intervals. Escape is defined as having HLA LOH or a mutation in HLA, *B2M* or other antigen presenting gene. Note that too few MSI metastases were present for associations to be calculated. The variables listed are tested relative to reference variables, which are (top-bottom, excluding quantitative and categorical variables): non-escaped; males; stage A/B; non-metastasis; and no prior non-surgical therapy. Purity, ploidy, age and TMB are quantitative variables; location (distal colon or rectum) is compared against proximal colon. *(c) Immune features of tumours and driver genes from different anatomical locations*. Top left: PHBR immunogenicity scores for 29 location-specific driver genes (11, 8 and 10 in proximal colon, distal colon and rectum respectively) in 1,049 MSS primary cancers. Top right: PHBR scores for subtype-specific driver genes (21 MSS primary, 5 MSS metastasis, 37 MSI, 16 POL) in 1,933 CRCs. Bottom left: frequencies of mutations in 18 driver genes common to different locations. Bottom right: PHBR of mutations in the 18 location-common driver mutations in each location. For box plots, centre line shows median, box limits show upper and lower quartiles, and whiskers show 1.5x inter-quartile range. Drivers specific to the distal colon had low overall immunogenic potential (median PHBR > 1) and lower immunogenicity (higher median PHBR) than proximal colon- and rectum-specific drivers (P_proximal v distal_ = 0.051; P_rectum v distal_ = 0.043). This also suggests that there is a stronger immune selection acting on drivers in the distal colon. Recurrent mutations in MSS driver genes were less frequent in distal than proximal CRCs (P = 0.012). However, the immunogenic potential of these mutations was near-identical between locations, suggesting that the observed depletion was not a consequence of site-specific driver immunogenicity. For example, *KRAS* G12D was detected in 18%, 7% and 12% of proximal colonic, distal colonic and rectal tumours, respectively (median PHBRs of 3.7, 3.9 and 3.6). Overall, the data are consistent with stronger immune surveillance in the distal colorectum, which lowers the threshold for tolerated immunogenicity, so that mutations that would be tolerated in the proximal colon are pruned in the distal colorectum. *(d) Immune escape mutations in MSS primary tumours from proximal colon, distal colon and rectum*. Cause of immune escape is colour coded. *(e) Neoantigen burdens in MSS primary tumours from proximal colon, distal colon and rectum*. n, numbers of cancers in each location. *(f) Neoantigen burdens in MSS primary tumours in regions 1-9 from caecum to rectum*. P value (two-sided) and correlation R are from Spearman’s rank correlation analysis. n, numbers of cancers in each location. For all panels, box plots are drawn as per panel (c) and statistical analyses used two-sided Wilcoxon tests, unless otherwise stated.

Predicted tumour neoantigen burden, summarized in Fig. 3a, was correlated with tumour mutation burden (TMB) (Pearson *R* = 0.89, *P* < 10^–16^, two-sided test)^39^. Antigenicity of selected common driver mutations is shown in Extended Data Fig. 7a. To examine the immunogenicity of all common driver mutations, we derived patient harmonic-mean best rank (PHBR) scores^40^, which quantify the potential of a mutation to generate a new human leukocyte antigen (HLA)-binding epitope depending on the HLA haplotype of the patient (Methods). We confirmed previous observations that the most commonly detected CRC driver mutations tended to have low immunogenic potential (Fig. 3b–d). Indeed, driver mutations were enriched in patients in whom they had a low immunogenic potential. Moreover, loss of HLA allele function through mutation or LOH reduced the immunogenicity of driver mutations (Fig. 3e). Differential immunogenicity analysis (that is, comparing the predicted immunogenicity of driver gene mutations in cancers with those mutations versus those without those mutations) identified five driver genes (*BRAF*, *TP53*, *SMAD4*, *PIK3CA* and *KRAS*) that had significantly higher mutation frequencies (*P*_Bonferroni_ < 0.1; Wilcoxon rank-sum test) in patients in whom their immunogenicity was predicted to be lower (Fig. 3f). Collectively, these observations are consistent with the idea that immune editing influences the driver landscape. However, the finding that the most common *KRAS* mutations are also more antigenic (Extended Data Fig. 7a) suggests that in some cases, direct positive selection can outweigh immunogenicity.

Several driver genes, especially in MSI and POL tumours, had a putative role in immunity and inflammation (Supplementary Table 4), specifically immune escape. As per other studies, patterns and prevalence of immune escape differed by CRC subtype^4,14,41,42^ (Fig. 3g). We separately evaluated allelic imbalance, LOH and protein-altering mutations in the *HLA-A*, *HLA-B* and *HLA-C* (MHC type I) genes and somatic mutations in a core set of other antigen-presenting or antigen-processing genes (APGs: *PSME3*, *PSME1*, *ERAP2*, *TAP2*, *ERAP1*, *HSPBP1*, *PDIA3*, *CALR*, *B2M*, *PSME2*, *PSMA7*, *IRF1*, *CANX*, *TAP1* and *CIITA*). Of these genes, *TAP2*, *B2M*, *IRF1*, *TAP1*, *HLA-A*, *HLA-B* and *HLA-C* were formally and independently classed as CRC drivers, with strongest signals in MSI cancers, but also discovered in MSS cancers (for example, *HLA-A* and *B2M*) (Fig. 3g and Supplementary Table 4). Multivariate regression analysis that accounted for clinical characteristics and TMB revealed that in MSS cancers, tumours with immune-escape mutations had a higher predicted neoantigen burden (*P* < 0.001; Fig. 3h). This association was present across all mechanisms of immune escape, but the HLA (type I) mutation had the strongest effect (associated with 21% increase in burden compared with HLA wild-type; *P* = 0.001). Conversely, in MSI cancers, only protein-altering mutations of HLA and other APGs were associated with higher neoantigen burden (*P* = 0.002 and *P* =1 × 10^−5^ respectively, Wilcoxon test), with an APG mutation corresponding to a 35% increase in the neoantigen burden. Immune escape from any mechanism remained significantly associated with neoantigen burden in multivariate regression (*P* = 0.012; Extended Data Fig. 7b). In MSI cancers, previous treatment (*n* = 34) was associated with an increased neoantigen burden independent of overall TMB (*P* = 0.006), a finding potentially linked to the genetic immune escape detected in 33 out of 34 treated MSI cancers.

## Beyond the coding nuclear genome

**Extended Data Fig. 8:**
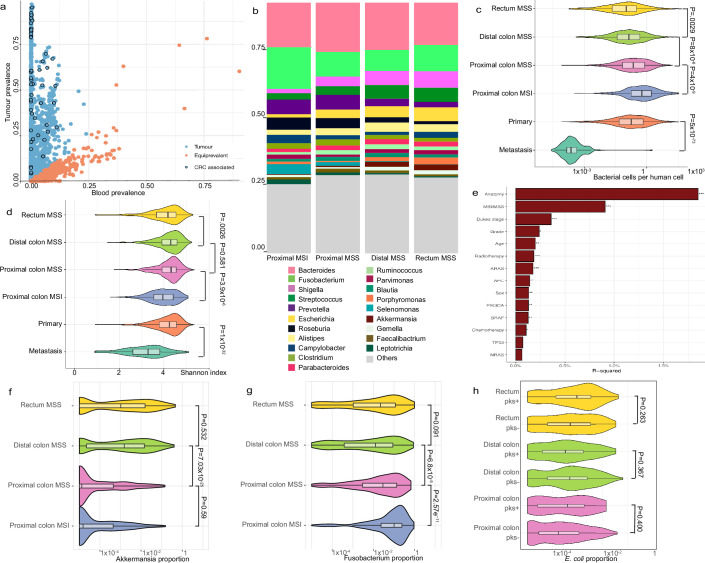
The CRC microbiome. *(a) Microbiome decontamination process*. Tumour and blood prevalence of all species are shown, according to methods based on The Cancer Microbiome Atlas. Orange points indicate taxa thought to be contaminants due to presence in both blood and tumour samples. Outlined points indicate species previously associated with CRC. *(b)* *Mean relative abundance of microbial genera for the four main CRC subtypes*. The most abundant 20 genera are shown. Other taxa are summed as “Others” for ease of visualisation. *(c) Bacterial load and (d) Shannon diversity index for different CRC groupings*. The 33 distal and rectal MSI cancers are not included, as the small cohort sizes do not allow meaningful comparisons. *P*-values for pairwise comparisons are displayed. *(e) Adonis PERMANOVA results comparing Bray-Curtis distances against various clinical and genomic factors*. R-squared is the percentage of diversity linked to each factor. Adonis *P*-value (two-sided) is indicated by symbol: * *P* < 0.05. ** *P* < 0.01. *** *P* < 0.001. *(f, g) Examples of two taxa distributions significantly associated with anatomical location for Akkermansia and Fusobacterium respectively*. Multivariate MaAslin2 *P*-values had been calculated from all samples and associations identified at P<0.05 (two-sided). Univariable *P*-values are shown in the panel, as these plots do not include distal or rectal MSI tumours. *(h) E. coli anatomical site distribution for pks-positive and -negative MSS CRCs. E.coli* proportions in tumours with either ID18 or SBS88 contributing to 5% or more of the mutational burden, compared to tumours with no pks contribution, are shown by anatomical location. No MSI tumours were pks-positive by these thresholds. *P*-values comparing pks-positive and -negative tumours for each location are shown. For panels (b-g), numbers of cancers were: rectum MSS 350; distal colon MSS 382; proximal colon MSS 454; and proximal colon MSI 282. Where reported, 1,898 primary tumours and 122 metastases were analysed. For panel (h), numbers of cancers were: rectum pks+ 101; rectum pks- 249; distal colon pks+ 51; distal colon pks- 331; proximal colon pks+ 28; and proximal colon pks- 426. For all box plots, the box is 25th to 75th percentile, the central bar is the median, and the whiskers are the largest/smallest values within 1.5 x interquartile range beyond the box. All P values are unadjusted from two-sided Wilcoxon tests unless otherwise stated.

To illustrate the utility of WGS in analysing features outside coding regions of the cancer genome, we performed five exemplar studies (details in Supplementary Result 8): (1) an exploration of driver mutations in regulatory noncoding elements (Supplementary Table 25); (2) recurrent, focal copy number changes and SVs outside fragile sites and gene bodies (Extended Data Fig. 6f and Supplementary Tables 12 and 16); (3) splice site driver mutations in *APC* and *SMAD4* (Supplementary Table 26); (4) the mitochondrial genome (Supplementary Table 27); and (5) the CRC-associated microbiome (Extended Data Fig. 8, Supplementary Tables 28–30 and Supplementary Result 9). A particularly promising finding in the noncoding human genome comprised recurrent, focal copy number deletions (chromosome 17: 72429007–72450223) in MSI tumours, involving the lincRNA LINC00673 (also known as LINC00511), a transcript that interacts with the CRC driver genes *EZH2* and *PTPN11* (Supplementary Table 16). This region overlapped with a SV deletion hotspot (chromosome 17: 72228421–72770582) in MSS primary tumours that includes a noncoding regulatory element that interacts with the promoter of the nearby CRC driver *SOX9* (Extended Data Fig. 6f and Supplementary Table 10).

## MSS CRC genomes by anatomical location

**Fig. 4:**
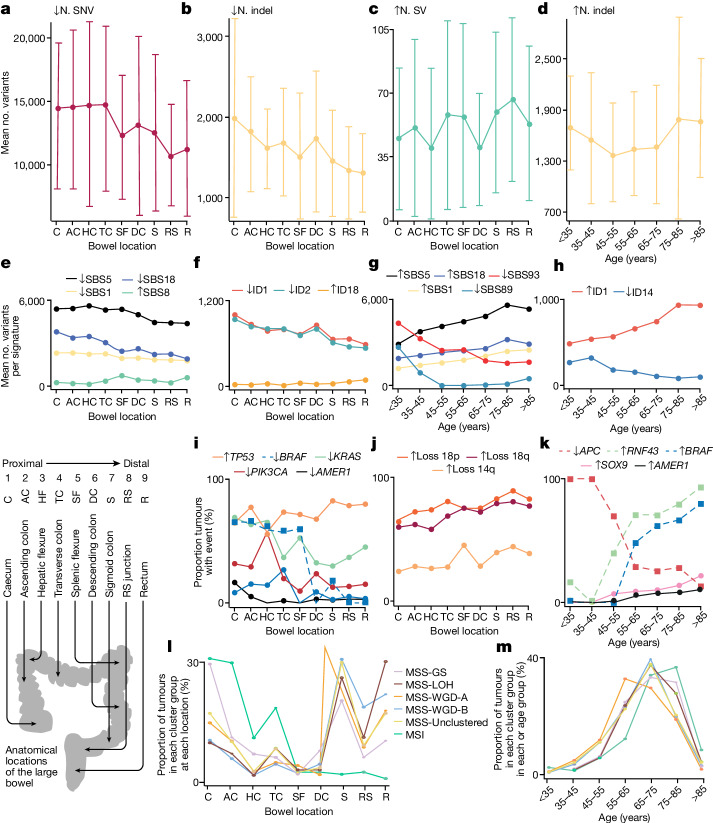
Variation of molecular features with MSS CRC anatomical location in the large bowel and with patient age at presentation. **a**–**d**, Mean number of variants (N) based on bowel location (**a**–**c**) and age (**d**)**. a**, Decreasing SNV burden from proximal to distal colorectum. **b**, Decreasing indel burden from proximal to distal colorectum. **c**, Increasing indel burden from proximal to distal colorectum. **d**, Increasing indel burden with age. **e**–**h**, Mean number of variants per signature based on bowel location (**e**,**f**) and age (**g**,**h**). **e**, Decreasing mutation burdens ascribed to SBS5, SBS18 and SBS1, and increasing *SBS8* burden, from proximal to distal colorectum. **f**, Decreasing mutation burdens ascribed to ID1 and ID2, and increasing ID18 burden, from proximal to distal colorectum. **g**, Decreasing mutation burdens ascribed to SBS93 and SBS89, and increasing SBS5, SBS18 and SBS1 burdens, with age. **h**, Decreasing mutation burdens ascribed to ID14, and increasing ID1 burden, with age. **i**, Decreasing frequencies of *KRAS*, *PIK3CA* and *AMER1* driver mutations, and increasing frequency of *TP53* mutations, from proximal to distal colorectum, with decreasing frequency of *BRAF* in MSI tumours shown for comparison. **j**, Increasing frequencies of arm-level CNAs involving chromosomes 18p, 18q and 14q from proximal to distal colorectum. **k**, Increasing frequencies of *SOX9* and *AMER1* driver mutations with age in MSS primary tumours compared with increasing frequencies of *RNF43* and *BRAF*, yet decreasing *APC*, with age in MSI tumours. **l**, Proportions of tumours in four MSS cluster groups, unclustered MSS and MSI showing increased MSS-GS (and MSI) in proximal locations and increased WGD-B in distal locations. **m**, As per **l** but by age, showing relatively early presentation of WGD-A cancers. Selected MSI data are shown by way of comparison in **i** and **k** using dashed lines. Error bars in **a**–**d** represent standard deviations. The bottom-left panel shows the nine anatomical sub-divisions of the colorectum, from caecum (most proximal) to rectum (most distal). RS, recto-sigmoid. Full data in these panels and additional data are provided in Supplementary Table 37, with further details in Extended Data Fig. 9 and Supplementary Tables 23 and 32–34.

**Extended Data Fig. 9:**
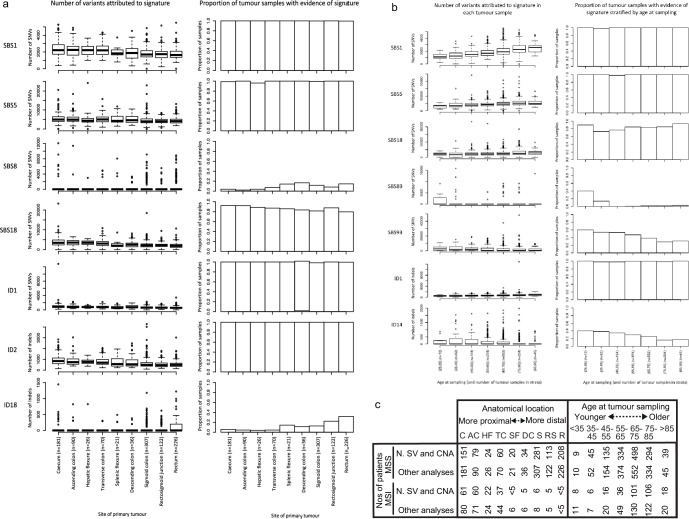
Further details of analyses by anatomical location and age. *(a) Location of primary tumour and number of variants attributed to mutational signatures in microsatellite stable (MSS) primary tumours*. Shown are mutational signatures associated with tumour location at a Bonferroni-corrected two-sided *P*-value of 0.05 using multiple linear regression considering age at sampling, sex, stage, grade and sample purity. n: number of tumour samples from location. *(b) Age at sampling and number of variants attributed to mutational signatures in primary MSS tumours*. Shown are mutational signatures associated with age at sampling (10 year bins) at a Bonferroni-corrected two-sided *P*-value of 0.05 using multiple linear regression considering sex, primary tumour location, stage, grade and sample purity. The Yeo-Johnson extension to the Box-Cox-transformation was applied to variant numbers. *(c) Numbers of patients included in anatomical location or age analyses*. Counts <5 are masked to prevent patient re-identification. In all panels, boxplots show the median value (thick black line), interquartile range (IQR; box bounds), and all outlying values (circles). Boxplot whiskers extend to the most extreme data point which are no more than 1.5 times the IQR from the box.

CRC is often said to comprise several different diseases depending on the tumour location^43^. As location co-varies with MSI status, we assessed the genomic features of MSS primary CRCs from different sites in the bowel. Tumours from distal locations had greater numbers of SVs and CNAs but fewer SNVs and indels (Fig. 4 and Supplementary Tables 31 and 32). Higher SBS8 and lower SBS1, SBS5, SBS18, ID1 and ID2 activities were also observed in cancers from distal sites^44^ (*P*_Bonferroni_ < 0.05, linear regression; Fig. 4, Extended Data Fig. 9a,c and Supplementary Table 32). The burden of *E*. coli^*pks*+^ and colibactin signature ID18 (but not SBS88) was higher in distal CRCs (*P* = 4 × 10^–10^, two-sided Wilcoxon test), a result consistent with healthy colon^10^ (Methods).

Distal MSS cancers were typified by higher frequencies of *TP53* mutations and lower frequencies of *AMER1*, *BRAF*, *KRAS* and *PIK3CA* mutations^9^ (Fig. 4 and Supplementary Table 33). Arm-level deletions of 14q, 18p and 18q also occurred more frequently in distal cancers (Fig. 4 and Supplementary Table 34), as did focal deletions of 1p36.11, 18q21.2, 18q22.3 and 20q13.33 gain. In part reflecting these specific changes, MSS cluster subgroups also showed associations with anatomical location (Fig. 2c, Extended Data Fig. 5a and Supplementary Table 23). The overall proportions of MSS-WGD-A, MSS-WGD-B and MSS-LOH tumours increased from the caecum to the rectum, whereas MSS-GS tumours were relatively common in the proximal colon.

Alongside the trend in indels, there was a decreasing trend in neoantigen burden from the caecum to the rectum (Extended Data Fig. 7b–e). There was no significant site-specific difference in the overall prevalence of immune-escape mutations (43% rectum, 39% distal colon, 38% proximal colon, *P* = 0.20, two-sided Kruskal–Wallis test, *n* = 1,019 MSS primary tumours). However, in rectal cancers, there was a higher prevalence of HLA LOH (*P* = 0.04, *χ*^2^). In a multivariate regression analysis including TMB and other patient co-variables, the distal colorectum was independently associated with lower neoantigen burden (Extended Data Fig. 7b), which suggested a higher level of immunoediting (*P*_distal colon_ = 9 × 10^–7^, *P*_rectum_ = 2 × 10^–4^; two-sided test).

## Driver gene discovery in CRC subgroups

As driver mutation frequencies varied along the bowel, we searched for location-specific driver genes based on a set of developmentally or clinically based anatomical subdivisions of the large bowel. We identified 48 drivers not found by our main analysis, most of which were detected in only a single location (Extended Data Fig. 2c and Supplementary Table 35). Nine of these drivers were previously unknown to any cancer and 35 were new drivers in CRC. These genes included *ETV1*, detected in the distal colon and previously proposed as a target of enhancer mutations in CRC^25^; the WNT transcription factor *LEF1* (proximal colon); *NOTCH2*, long proposed to have a role in CRC pathogenesis (distal colorectum)^45^; the oncogene *SRC* (distal colorectum); the PI3K–mTOR signalling molecule *TFEB* (rectum); and the EGFR signalling component *DDR2* (proximal colon).

Because the frequencies of some driver genes varied significantly among MSS clusters, we reasoned that cluster-specific drivers might exist. Exploratory driver discovery in each of the 4 cluster subgroups identified 35 additional candidate drivers (Supplementary Table 36). These included four genes detected in two subgroups (*BRCA2*, *COL1A1*, *PTPRT* and *SMARCA4*) and other strong candidates such as *ACVR1*, *NOTCH1* and *POT1*.

## Molecular correlates of early-onset CRC

Recent reports of an increase in early-onset CRCs^46,47^ are currently unexplained. We found that individuals with Mendelian syndromes or somatic *POLE* mutations presented earlier in life (median age of 60 years at sampling, range 34–79 years, *P* = 0.0015, Wilcoxon test), as expected^37^. SNV and SV burden were not correlated with age, but in MSS cancers, indel burden was highest in the youngest and oldest patients (<45 years old, mean = 13,428; 45–75 years old, mean = 12,328; >75 years old, mean = 13,906; *P* < 0.05, pair-wise Wilcoxon tests against the 45–75-year-old group) (Fig. 4, Extended Data Fig. 9b,c and Supplementary Table 32). Younger patient age was associated with lower activities of SBS1, SBS5 and ID1 (clock-like signatures) and SBS18 (reactive oxygen species)^15,48^. By contrast, SBS89, SBS93 and ID14 activities were higher in younger patients. The association between SBS93 and earlier age was strong (multiple regression, *P* = 3.3 × 10^–7^, two-sided test), and accounted for a younger presentation of about 5 years. Similar to SBS93, SBS89 has unknown aetiology, although it has been reported to occur in healthy colon tissue during the first decade of life^44^. Younger age also correlated with lower *SOX9* pathogenic mutation frequency in MSS primary cancers. In primary MSI cancers, frequencies of *BRAF* and *RNF43* mutations were lower in younger patients, with correspondingly higher *APC* frequency (*P* < 0.05, two-sided Wilcoxon test; Fig. 4 and Supplementary Table 33).

## Concluding remarks

Here we provided a large and comprehensive analyses of the genomic landscape of more than 2,000 patients with CRC. In addition to providing a comprehensive set of mutations of all types, a principal strength of our study is the ability to detect uncommon features, as evidenced by the discovery of many new driver genes, including SNVs, small indels, SVs and CNAs. Although some rare driver mutations might have uncertain driver status or weakly promote tumorigenesis, others may have considerable relevance, especially if they are known drivers in other cancer types or overlap functionally with other rare drivers that collectively form a higher frequency group.

In addition to the discovery of driver genes, several new insights into CRC genomics and biology were obtained (Supplementary Note). We showed that the large MSS group of CRCs is not a homogenous entity by clustering it into four common subgroups with distinct molecular and clinicopathological features. We also discovered and better characterized rare CRC subgroups, including MSI CIN CRCs, cancers with parallel evolution of copy number and SNV driver mutations, and tumours with putative noncoding driver mutations. We found new mutational signatures in CRC and molecular features associated with early-onset disease or tumour location in the large bowel, the latter showing that proximal MSS CRCs share some features with MSI tumours. We showed evidence of immune editing of driver mutations and frequent immune-escape mutations, especially in MSI and POL hypermutant cancers. All these results have potential clinical implications or utility. We anticipate that our work will fuel future studies, including efforts to characterize putative driver genes, translational analyses and multidisciplinary experiments to address specific questions in a focused fashion.

## Methods

### Sample collection

The following steps were taken for sample collection. (1) Ethics approval was provided to the 100kGP by the HRA Committee East of England–Cambridge South research ethics committee (REC reference 14/EE/1112). Samples were obtained as part of the 100kGP cancer programme, an initiative for high-throughput tumour sequencing for NHS patients with cancer^49,50^. (2) Thirteen Genomic Medicine Centres (GMCs) were established by the NHS and 100kGP, each with multiple affiliated hospitals across in the same region of the UK. (3) Patients undergoing resection for CRC were identified by specialist nurses and other staff. (4) All patients provided written informed consent, and blood samples were taken. (5) Tumour samples were assessed in histopathology cut-ups. Associated clinicopathological data were obtained from health records. (7) Frozen tumour sub-samples were taken and frozen. Haematoxylin and eosin sections were assessed for purity and other histological features of note. (8) Blood and tumour samples that passed quality control were sent for DNA extraction in regional genetics laboratories. (9) DNA was transferred to the 100kGP central national biorepositry. (10) WGS of paired tumour-constitutional (whole blood-derived) DNA was performed by Illumina. (11) Processed BAM files were transferred to Genomics England for additional processing, quality checking and data storage. (12) All sequencing and clinicopathological data were transferred to Colorectal Cancer Domain (GECIP) for further quality control and data analysis.

### WGS and SV calling

Sequencing, mapping and variant calling were generally performed as previously described^51^, although we used a less stringent variant allele frequency (VAF) to enable analyses of subclonal mutations.

#### Sequencing and alignment

Samples were prepared using an Illumina TruSeq DNA PCR-free library preparation kit and sequenced on a HiSeq X, generating 150 bp paired-end reads. Tumour and constitutional DNAs were sequenced to average depths of 100× and 33×, respectively. Poor sequencing quality outliers were identified using principal component analysis and removed on the basis of the following quality metrics: percentage of mapped reads; percentage of chimeric DNA fragments; average insert size; AT/CG dropout; and unevenness of local coverage. Illumina’s North Star pipeline (v.2.6.53.23) was used for the primary WGS analysis. Sequence reads were aligned to the *Homo sapiens* GRCh38Decoy assembly using Isaac (v.03.16.02.19)^52^. Overall, PCR-free tumour and germline sequencing data for 2,492 fresh-frozen CRC samples were obtained from the 100kGP main program (v.8) release and used in our analysis.

#### Single-nucleotide variant and indel calling

Single-nucleotide variant and small indel calling was performed using Strelka (v2.4.7). In addition to the default Strelka filters, we applied the following exclusion filters:

- Variants with a germline allele frequency > 1% in the full Genomics England dataset.
- Variants with a population germline allele frequency > 1% in the gnomAD database^53^.
- Somatic variants with frequency > 5% in the Genomics England cancer dataset. A 5% cut-off was chosen based on the frequency of recurrent non-synonymous variants in Cancer Gene Census genes^54^.
- Variants overlapping simple repeats as defined by Tandem Repeats Finder^55^.
- Indels in regions with high levels of sequencing noise where >10% of the base calls in a window extending 50 bp either side of the indel were filtered out by Strelka owing to the poor quality.
- Indels within 10 bp of 100kGP or gnomAD (v.3) germline indel with allele frequency > 1%.
- Variants in regions of poor mappability where the majority of overlapping 150 bp reads do not map uniquely to the variant position.
- SNVs resulting from systematic mapping and calling artefacts present in both tumour and control 100kGP sample sets. We tested whether the ratio of tumour allele depths at each somatic SNV site was significantly different to the ratio of allele depths at this site in a panel of control samples using Fisher’s exact test. The panel of control was composed of a cohort of 7,000 non-tumour genomes from the Genomics England dataset. At each genomic site, only individuals not carrying the relevant alternative allele were included in the count of allele depths. The mpileup function in bcftools (v.1.9) was used to count allele depths in the PoN. To replicate Strelka filters, duplicate reads were removed and quality thresholds set at mapping quality ≥ 5 and base quality ≥ 5. All somatic SNVs with a Fisher’s exact test phred score < 80 were filtered, with the threshold determined by optimizing precision and recall calculated from a TRACERx truth set^56^.

#### Removing alignment bias introduced by soft clipping of semi-aligned reads

The Isaac --clip-semialigned parameter invokes the soft clipping of read ends until five consecutive bases are matched with the reference genome. This soft clipping therefore results in the loss of support for alternative alleles occurring within 5 bp of each read end, which leads to artefactually low VAFs. To address allelic bias introduced by this clipping, we introduced FixVAF to soft clip all reads by 5 bp at each end, regardless of whether any of the bases are variant sites or whether the reads support reference or alternate alleles^57^. Reads containing small indels at variant positions were ignored (Supplementary Fig. 1).

#### Identifying MSI

Tumours with MSI were identified using MSINGS^58^ following the previously described procedure for background model generation (https://github.com/sheenamt/msings/blob/master/Recommendations_for_custom_assays). A set of 132 tumours with known MSI status (106 MSS, 26 MSI) was randomized into test and training sets of 53 MSS and 13 MSI cases (that is, 2 sets of 66 cases). Microsatellite sites were generated using MISA^59^. Only sites overlapping regions of good mappability were considered. Sites measured as unstable in >5 MSS test tumours and sites not unstable in >1 test MSI tumours were removed. The background model produced using the training set was able to perfectly distinguish between MSI and MSS samples in the test set using default MSINGs settings and was then applied to the full CRC cohort.

#### Identifying pathogenic POL variants

Tumours with pathogenic somatic or germline variants in *POLE* or *POLD1* were identified considering the 22 known pathogenic variants a previously reported^60^. In total, 18 tumours (17 MSS, 1 MSI) had a pathogenic germline (*n* = 1) or somatic (*n* = 17) *POLE* variant and these were considered as a separate POL group in all subsequent analyses. All of the highest mutational burden tumours were either MSI or had a known pathogenic *POLE* variant, which indicated that no pathogenic polymerase proofreading domain mutations were missed. Tumours with pathogenic *POLE* variants also exhibited high SBS10a and SBS10b activity, which are established indicators of *POLE* exonuclease domain mutations^11^.

#### CNA calling

Somatic CNAs were called using a framework implemented in the R package CleanCNA (Supplementary Fig. 2). Genome-wide subclonal CNAs were first called using Battenberg (v.2.2.8)^61^. To check the quality of these CNA calls, we applied DPClust^61^ and CNAqc^62^ to the CNA profiles and SNV VAFs. DPClust clusters variants by their cancer cell fraction (CCF), whereas CNAqc compares observed and expected peaks in SNV VAF distributions to assess CNA calling accuracy. A sample was classified as ‘pass’ if it met both of the following criteria, and ‘fail’ otherwise as follows:

1. A clonal cluster of SNVs (0.95 ≤ CCF ≤ 1.05) was identified by DPClust. This clonal cluster was required to have either the highest CCF of all SNV clusters or contain the largest number of SNVs. SNV clusters containing <1% of all sample SNVs were removed before assessment.
2. The difference in purity estimates from Battenberg and CNAqc was <5%. CNAqc estimates sample purity considering peaks in SNV VAF distributions in genome regions with one of five copy number states (1:0, 1:1, 2:0, 2:1, 2:2).

CNAs were profiled a maximum of four times per sample and the procedure was stopped if both criteria were met. After a failure, CNA were re-called using Battenberg with re-estimated sample purity and tumour ploidy. After the first fail, purity and ploidy were re-estimated using information from DPClust, where CCF_top_ is the CCF of the SNV cluster with the greatest CCF:

$$\begin{array}{c}{p}_{{\rm{new}}}={\rho }_{{\rm{old}}}\,{{\rm{CCF}}}_{{\rm{top}}}\\ {\varPsi }_{{\rm{new}}}=\,(({\rho }_{{\rm{old}}}\,{\psi }_{{\rm{old}}})+2({\rho }_{{\rm{new}}}-{\rho }_{{\rm{old}}}))/{\rho }_{{\rm{new}}}\end{array}$$

After the second fail, purity and ploidy were re-estimated using Ccube^63^, and after the third and fourth fails, purity and ploidy were re-estimated using CNAqc. If a sample failed after four re-runs, then it was removed from downstream analyses reliant on CNAs. Pass CNA profiles were produced for 1,765 out of 2,023 samples.

#### SV calling

SVs (also referred to as chromosomal rearrangements) represent two reference positions (referred to as rearrangement breakpoints) that are non-adjacent in the reference genome and juxtaposed in a specific orientation. We identified somatic rearrangements using a graph-based consensus approach comprising Delly^64^, Lumpy^65^ and Manta^66^ while also considering support from CNAs (Supplementary Fig. 3). Rearrangements were first called using the three individual callers with default parameters. Delly was run with post-filtering of somatic SVs using all normal samples, as described in the Delly documentation. Rearrangements from the three individual callers were further filtered if any reads supporting the variant were identified in the matched normal, if <2% of tumour reads supported the variant or if either variant breakpoint was in a telomeric or centromeric region or on a non-standard reference contig (that is, not chromosomes 1–22, X or Y). Remaining rearrangements were merged with a modified version of PCAWG Merge SV, which uses a graph-based approach to identify and merge rearrangements identified by multiple callers, allowing a maximum 400 bp difference in breakpoint position to account for variant calling ambiguity^16^. Rearrangements were included in the final dataset if they were identified by at least two callers, or by a single caller but with a breakpoint within 3 kb of a CNA segment boundary. SVs were only called in the 1,765 out of 2,023 samples with CNA profiles passing quality control criteria.

Retrotransposition events are mechanistically distinct from other SV-generating events. We searched for retrotransposition events using xTea for LINE-1 elements^67–69^, as other retrotransposition categories (Alu elements, SINE-VNTR-Alu elements and processed pseudogenes, among others) collectively constitute ≤3% of retrotransposition events across human cancers^66^. We subsequently decided to exclude retrotranspositions from our current SV analysis report, to await later separate publication.

Putative kinase gene fusions were identified considering the following genes: *ALK*, *BRAF*, *EGFR*, *ERBB2*, *ERBB4*, *FGFR1*, *FGFR2*, *FGFR3*, *KIT*, *MET*, *NTRK1*, *NTRK2*, *NTRK3*, *ROS1* and *RET*^22^. Fusions were required to involve the kinase domain of the 3′ gene and to have correct strand orientation.

### Clinical data

Clinical data were obtained from the GMCs, NHS Digital (NHSD) and Public Health England’s National Cancer Registration and Analysis Service (PHE-NCRAS) through the Genomics England Research Environment as part the 100kGP main program v.10 release. Survival data were obtained from the 100kGP main program v.13 release. Tumour samples sequenced by Genomics England were matched to their respective PHE-NCRAS records using the date of tumour sampling reported by Genomics England and dates of biopsy or treatment reported by PHE-NCRAS, allowing a maximum discrepancy of 7 days.

Clinical data included sex, age at tumour sampling, date of cancer diagnosis, date of last reported follow-up and date of death, tumour histology, tumour type (primary, recurrence of primary or metastases), anatomical site sampled, anatomical site of primary tumour, Dukes stage, and tumour grade (differentiation). For some variables, data were obtained from multiple sources (GMC, NHSD, PHE-NCRAS), and any conflicts between these sources were resolved by individual inspection. If Dukes staging was not available, it was inferred from TNM staging if reported. Anatomical site of primary tumour was reported at different resolutions by the different data sources (for example, one source may report site as proximal colon, whereas another may report it as caecum). To resolve and standardize the site, we therefore constructed an anatomical ontology based on ICD-10-CM codes and assigned sample terms to this ontology. This enabled us to consider anatomical site at two main levels of resolution: less specific (proximal colon, distal colon and rectum) and more specific (caecum, ascending colon, hepatic flexure, transverse colon, splenic flexure, descending colon, sigmoid colon, rectosigmoid colon and rectum). Certain analyses were also performed on the basis of a combined analysis of proximal and distal colon (colon). The proximal colon comprised the caecum, ascending colon, hepatic flexure and transverse colon, whereas the distal colon comprised the splenic flexure, descending colon and sigmoid colon. The rectosigmoid junction was considered part of the rectum. All associations between clinical and molecular data, and between different molecular data, are reported based on tests unless otherwise stated.

Germline mutations in the Mendelian CRC predisposition genes (*APC*, *MSH2*, *MLH1*, *MSH6*, *MUTYH*, *SMAD4,* *BMPR1A*, *GREM1*, *STK11*, *NTHL1*, *MBD4*, *POLE* and *POLD1*) were explored in the sequenced constitutional DNA. Disease-causing changes were identified based on ClinVar annotation as ‘pathogenic’ or ‘likely pathogenic’, with the exception of *POLE* and *POLD1*, which used the method described in the section ‘Identifying pathogenic POL variants’. Evidence of pathogenic biallelic changes was required to diagnose the recessive conditions (*MUTYH*, *NTHL1* and *MBD4*) and no such cases were found. Twenty patients (aged 30–79 years) were identified as having a previously unreported CRC predisposition caused by germline mutations in Lynch syndrome or polymerase proofreading polyposis genes (seven *MSH2*, five *MLH1*, six *MSH6*, one *POLE*, one *POLD1*).

Based on principal component analysis of germline genotypes, 90.2% (*n* = 1,819) patients were of European ancestry, with 2.6% (*n* = 52) African, 0.7 (*n* = 15) East Asian, 3.2% (*n* = 64) South Asian and 3.3% (*n* = 67) mixed ancestry (Supplementary Fig. 4). There was strong agreement between 16 self-reported ancestry groups and principal component analysis classification.

### Sample selection

Because tumour sample purity and sequencing data quality affect the sensitivity and precision of variant calling^70^, we excluded samples using the following quality control procedures (Supplementary Table 2).

- Tumour samples were excluded if cross-contamination of the tumour sample was >1%, as estimated by VerifyBamID^71^.
- Tumour samples were excluded if cross-contamination of the matched germline sample was >1%, as estimated by VerifyBamID.
- Estimating tumour sample purity is particularly difficult when purity is low. We therefore used the distribution of single-nucleotide variant VAFs to identify low purity samples, as a low average SNV VAF can be indicative of low sample purity^72^. Tumour samples with a median SNV VAF < 0.1 were excluded, with this threshold chosen based on the smaller numbers of potential driver variants observed in MSS CRC samples when compared with all MSS CRC samples (Supplementary Fig. 5). Here driver mutations were defined as any potentially pathogenic coding variant called in 63 driver genes previously identified in MSS CRC^3,4,7,8^.
- Tumour samples were excluded if <100 SNVs were called, as this number is below the smallest number of SNVs previously reported in CRC whole genomes^2–9^ and therefore suggestive of low sample purity or sequencing data quality.
- Tumour samples were excluded if many mutations were associated with a probable artefactual mutational signature^15^.

In total 286 out of 2,492 (11.5%) tumour samples were excluded based on the above criteria.

Tumour samples were also excluded if essential clinical data were missing or there were unresolvable conflicts between the sources from which clinical data were obtained (GMCs, NHSD, PHE-NCRAS) (Supplementary Table 2). In total, 183 out of 2,206 (8.3%) of tumour samples that passed tumour sample purity and sequencing data quality control were excluded based on clinical data, using the following criteria:

- GMC, NHSD and PHE-NCRAS reported conflicting years of birth.
- Sex reported by GMC, NHSD and/or PHE-NCRAS did not match the sex inferred from sequencing data.
- GMC, NHSD and PHE-NCRAS did not report tumour histology or reported conflicting histology.
- Tumour was not classified as a colorectal adenocarcinoma.
- Missing or conflicting data meant it was unclear whether the primary tumour or a metastasis was sampled.
- If multiple primary tumours or multiple metastases from a single individual were sequenced, the primary tumour or metastasis sample with the highest purity was included, and all other primary tumour or metastasis samples were excluded. This procedure was completed after all other exclusion criteria had been applied. Primary tumours and metastases were considered separately for this procedure.

Based on these criteria, 2,023 colorectal adenocarcinoma samples were suitable for analysis (Supplementary Table 2). This cohort comprised 1,898 primary tumours, 122 metastases and 3 recurrences of primary tumours from 2,017 patients. Six patients (all MSS) had both a primary tumour and a metastasis sample sequenced and each tumour was included. One hundred and nineteen metastases were MSS, the other three comprising two MSI and one POL cancer. Some subsequent analyses excluded the MSI and POL metastases (details in Supplementary Tables). The three recurrences were MSS (*n* = 1) and MSI (*n* = 2), and these were included in the appropriate primary cancer group for further analyses. A single cancer was POL and MSI, and this was included in the POL group for further analyses. Clinical data completeness is detailed in Supplementary Table 31.

### Single-nucleotide variant and indel drivers

#### Mutation annotation

Somatic mutations were annotated to Ensembl (v.101, GRCh38) using Variant Effect Predictor (VEP)^73^. The following parameters were used: vep -i <input_vcf> --assembly GRCh38 –no_stats –cache –offline –symbol –protein -o <output> --vcf –canonical –dir <ref_dir> --hgvs –hgvsg –fasta <GRCh38_fasta> --plugin CADD,<CADD_score_file> --plugin UTRannotator,<GRCh38_uORF_reference>.

The CADD score file was obtained using CADD (v.1.6)^74–76^, with scores attained for all SNV and indel mutations using the CADD software available from GitHub (https://github.com/kircherlab/CADD-scripts) before being utilized by the VEP CADD plugin.

#### Protein-coding driver identification

Protein-coding driver genes were identified using the IntOGen pipeline (v.2020, downloaded February 2021)^18^. Identification was performed separately in MSS primary, MSI (all primary), POL (all primary) and MSS metastasis sample sets, with the aim of optimizing correction for varying background mutation rates and spectra among these four groups. Subsequent analyses restricted discovery to specific anatomical locations or cluster groups in MSS primary tumours.

#### Pre-processing of input mutations

Somatic mutations passing the filtering criteria described above were subject to initial sample and mutation pre-processing. In the case of multiple tumours from the same patient, the primary tumour was used. Within each cohort (that is, MSS primary, primary MSI, primary POL, MSS metastasis), tumours were flagged for exclusion from downstream driver gene identification if they contained >10,000 mutations and had an outlier mutation count, defined as upper quartile + (1.5 × interquartile range). Mutations present in a Hartwig Consortium panel of control set were also excluded^77^. Unless otherwise specified, mutations were mapped to canonical protein-coding transcripts from Ensembl (v.101, GRCh38).

#### Driver identification methods

Seven driver gene identification methods were run through the IntOGen pipeline (Supplementary Fig. 6):

1. dNdSCV (v.0.1.0)^6^ is designed to detect genes under positive selection that show an excess of non-synonymous (missense, nonsense, essential splice) mutations after correction for local trinucleotide context. In the primary POL cohort the parameter ‘max_coding_muts_per_sample = Inf’ was used because of the high proportion of hypermutated tumours.
2. OncodriveFML (v.2.4.0)^78^ aims to detect driver genes that show an enrichment of mutations with high functional impact. CADD scores were used as measure of functional impact^74–76^.
3. OncodriveCLUSTL (v.1.1.3)^79^ is a method designed to detect driver genes that are enriched for linear mutation clusters. In the primary POL cohort, pentamer signatures were used rather than trinucleotide signatures because of the improved performance of the pentanucleotide-based background models compared with that of trinucleotides in these tumours.
4. cBaSE (v.1.1.3)^18,80^ aims to detect driver genes under positive selection that exhibit a significant mutation count bias after correction by trinucleotide context.
5. MutPanning (v.2)^81^ is designed to detect driver genes that exhibit enrichment of mutations with unusual nucleotide contexts compared with a background model.
6. HotMaps3D (v.1.1.3)^18,82^ detects driver genes containing missense mutations that are spatially clustered together in the three-dimensional structure of the protein. Protein structures were downloaded from The Protein Data Bank^83^ in March 2020.
7. smRegions (v.1)^84^ detects genes containing an enrichment of non-synonymous mutations in regions of interest, such as protein domains, after correcting for trinucleotide context. This analysis utilized information from protein family (Pfam) domains that were mapped to Ensembl (v.101) canonical transcripts.

#### Combination of driver identification methods

The results of the seven driver identification methods were combined in similar manner as previously described^18^. In brief, the driver combination procedure considered the top 100 ranked genes and their associated *P* and *Q* values in each of the seven driver identification methods. Somatically mutated genes assigned as tier 1 or tier 2 in the COSMIC Cancer Gene Census (CGC; v.92)^54^ were designated as the truth set of known drivers. Through comparison of the relative enrichment of CGC genes in the top ranked gene lists, a per-method weighting was obtained. Per-method ranked lists were combined using Schulze’s voting method to generate a consensus ranking, with combined *P* values estimated using a weighted Stouffer *Z* score method.

Driver candidates were then classified into the following tiers:

- Tier 1: candidates for which the consensus ranking was higher than the ranking of the first gene with Stouffer *Q* ≤ 0.05. These represent high-confidence drivers.
- Tier 2: candidates not meeting the criteria for tier 1, but which are CGC genes and showed a combined Stouffer *Q*_CGC_ < 0.25. These represent a set of ‘rescued’ known cancer drivers.
- Tier 3: candidates not meeting the criteria for tier 1 or tier 2 but with Stouffer *Q* < 0.05. These represent lower confidence drivers.
- Tier 4: candidates not meeting criteria for tier 1 or tier 2 and with Stouffer *Q* > 0.05. These genes are not likely to be drivers.

#### Post-processing of candidate drivers

Candidate driver genes were filtered based on the following annotations:

1. Automatic fail: a candidate driver gene would be excluded from further consideration if annotated with at least one of the following:
  - Tier 4: categorized as tier 4 by the combination procedure.
  - Single method: only significant (*Q* < 0.1) in one of the seven methods (non-CGC genes).
  - Expression: gene has very low or no expression in a relevant tumour type based on data from The Cancer Genome Atlas (TCGA).
  - Olfactory receptor: gene is in list of olfactory receptor genes.
  - Known artefact: gene is in a list of known artefacts or long genes (for example, TTN).
2. Manual review: if a gene is not excluded based on any automatic fail filters, it is retained as a candidate driver:
  - Germline: non-tier 1-CGC gene has ≥1 mutations per sample and oe_syn/ms/lof > 1.5 based on gnomAD (v.2.1) constraint metric estimates.
  - Sample 3 Muts: non-CGC gene for which there are ≥3 mutations in ≥1 tumour.
  - Literature: non-CGC gene for which there are no literature annotations according to CancerMine^85^.
3. Automatic pass: is not flagged by any automatic fail or manual review filters.

Candidate driver roles were assigned on the basis of dN/dS ratios for missense (wmis) and nonsense (wnon) mutations for the given gene derived from dNdSCV (https://bitbucket.org/intogen/intogen-plus/src/master/core/intogen_core/postprocess/drivers/role.py):

- A distance metric was calculated by distance = ((wmis – wnon))/√2
- Candidate drivers with distance >0.1 represent those with an excess of missense to nonsense mutations and are therefore considered oncogenes.
- Candidate drivers with distance <0.1 represent those with an excess of nonsense to missense mutations and are therefore considered TSGs.
- Otherwise, the role of the candidate driver is unclear and considered ambiguous.

In the case of multiple cohorts being run representing subsets of a given tumour type, a consensus role was designated comparing between each subtype role:

- Oncogene if assigned as oncogene in ≥1 cohort and as TSG in no other cohort.
- TSG if assigned as TSG in ≥1 cohort and as oncogene in no other cohort.
- Ambiguous otherwise.

Gene candidates were annotated by their overlap with any IntOGen cohorts from a previous IntOGen pan-cancer analysis (1 February 2020) as well as from a pan-cancer TCGA analysis^2^.

### Noncoding driver identification

#### Defining sets of noncoding regions

Regions from candidate noncoding elements overlapping coding sequence (CDS) or exon regions from canonical protein-coding transcripts were removed using bedops (v.2.4.39)^86^.

The following sets of noncoding regions were defined:

1. Core promoters (*n* = 19,283). Defined based on the transcription start site (TSS) of canonical protein-coding transcripts: 200 bp < TSS < 50 bp. CDS regions were removed.
2. Distal promoters (*n* = 19,296). Defined based on the TSS of canonical protein-coding transcripts: 2 kb < TSS. CDS regions were removed.
3. 5′ untranslated regions (UTRs; *n* = 18,613). Defined based on canonical protein-coding transcripts. CDS regions were removed.
4. 3′ UTRs (*n* = 18,806). Defined based on canonical protein-coding transcripts. CDS regions were removed.
5. lincRNAs (*n* = 16,510). Based on exon regions from transcripts annotated as lincRNAs in Ensembl (v.101). Exon regions from canonical protein-coding transcripts were removed.
6. miRNAs (*n* = 1,793). Based on regions from transcripts annotated as miRNAs in Ensembl (v.101). Exon regions from canonical protein-coding transcripts were removed.
7. Non-canonical splice regions (*n* = 18,163). Defined from regions extending 30 bp into the intron from essential splice donor or acceptor sites in canonical protein-coding transcripts. Exon regions from canonical protein-coding transcripts were removed.
8. Enhancers (*n* = 130,996). Defined from Ensembl (v.101) regulatory elements annotated as ‘enhancer’. Exon regions from canonical protein-coding transcripts were removed.
9. Open chromatin regions (*n* = 95,344). Defined from Ensembl (v.101) regulatory elements annotated as ‘open chromatin’. Exon regions from canonical protein-coding transcripts were removed.
10. CTCF sites (*n* = 173,711). Defined from Ensembl (v.101) regulatory elements annotated as ‘CTCF sites’. Exon regions from canonical protein-coding transcripts were removed.
11. Transcription factor-binding sites (*n* = 29,259). Defined from Ensembl (v.101) regulatory elements annotated as ‘TF binding sites’. Exon regions from canonical protein-coding transcripts were removed.

#### Detecting noncoding drivers

Potential noncoding driver mutations were identified in non-hypermutated MSS primary tumours (*n* = 1,442). OncodriveFML (v.2.4.0) was run on sets of noncoding regions according to the following amended parameters from the protein-coding analysis: indel-max indels are treated as a set of substitutions, with the functional impact of the indel mutation being the maximum of all the substitutions, and the background simulated as substitutions. A *Q* < 0.01 threshold was considered as significant (Supplementary Fig. 7).

### SNV mutations exhibiting extreme strand bias

SNV mutations that otherwise passed filtering criteria as previously detailed were further scrutinized for excessive strand bias (Strelka INFO field SNVSB > 10). This highlighted many missense mutations that cause a recurrent missense change in *CACNA1E* (p.Ile95Leu); these exhibited excessive strand bias and were therefore deemed false calls.

### Driver mutation annotation

Non-synonymous mutations in the 682 gene transcripts considered by OncoKB (v.3.3) were annotated using the OncoKB API^87^. In the first instance, the HGVSg identifier was used; in the rare instances that this failed, a combination of gene symbol, consequence and HGVSp were used to map mutations to OncoKB annotations.

### Annotation of oncogenic mutations

Non-synonymous mutations in candidate driver genes were annotated as pathogenic if any of the following criteria were met:

1. The mutation is annotated by OncoKB as ‘oncogenic, ‘likely oncogenic’ or ‘predicted oncogenic’.
2. The driver is classified as an oncogene, the mutation consequence is missense, and the mutation is recurrent (seen in ≥3 tumours in cohort).
3. The driver is classified as a TSG or ambiguous and either:
  - Consequence is protein-truncating (splice acceptor, splice donor, frameshift, stop lost, stop gained or start lost).
  - Consequence is missense and mutation is recurrent (seen in ≥3 tumours in cohort).

For *POLE*, oncogenic annotations were restricted to missense mutations in the exonuclease domain (amino acid residues 268–471).

Non-synonymous mutations not meeting these criteria were considered as variants of uncertain significance.

#### Lollipop plots of driver gene mutations

Lollipop plots of driver gene mutations (Supplementary Result 2) were generated using the Rpackage trackViewer^79^. Pfam protein domains mapping to the Ensembl (v.101) canonical transcripts were plotted. The protein position was taken from the first position in the HGVSp annotation, apart from splice donor and acceptor mutations, for which the codon nearest to the HGVSc transcript position was assigned as the protein position.

#### Timing driver mutations

The relative evolutionary timings of candidate driver mutations were obtained using MutationTimeR^31^. Copy number input for MutationTimeR was prepared from Battenberg segmentation files, with the clonal frequency of each segment taken as the tumour purity. In the case of subclonal calls, the clonal frequency was calculated by multiplying the tumour purity by the clonal fraction. The clusters input for MutationTimeR was prepared from DPClust cluster estimates. The VAF proportion was calculated by multiplying the estimated cluster CCF by the tumour purity. Superclonal clusters (CCF > 1.1) were removed. VCF input for MutationTimeR was obtained from the small somatic SNV/indel variant VCFs, which had been filtered as previously described. For SNVs, alt and ref depths were obtained using FixVAF. For indels, ref and alt depths were obtained from tier 2 Strelka TAR and TIR fields, respectively. Only mutations within Battenberg copy-number segments were retained (note that for male XY tumours with only 1 copy of the X chromosome, copy number information is restricted to the pseudoautosomal region and Battenberg was not run on the Y chromosome).

MutationTimeR was run with 1,000 bootstraps. For tumours previously defined as having undergone WGD, the parameter isWgd was set to true. Mutations were then classified into estimated simple clonal states (as per figure 1a of ref. ^31^): clonal (early), mutation on ≥ 2 copies per cell; clonal (late), mutation on 1 copy per cell, no retained allele; clonal (NA), mutation on 1 copy per cell, either on amplified or retained allele; subclonal, mutation on <1 copy per cell.

#### Mutational signature attribution

SeqInfo VCFs produced as part of SigProfilerMatrixGenerator^17^ were used to map somatic mutations from input VCFs to their SBS96, DBS78 or ID83 contexts and then to the final SigProfilerExtractor COSMIC (v.3.2) decomposed signature probabilities. For different purposes, mutational signatures were variously measured as follows: presence–absence, for example, when assessing shared aetiology; proportional activity (essentially proportion of mutations fitted to any signature in that tumour), useful for comparing between signatures in the same sample; and number of mutations ascribed, estimated as (activity × burden of mutations of SBS, DBS or ID type fitted to any signature), approximating to burden of mutations from that signature in that tumour.

#### Annotation of DBS mutations

Per-tumour VCFs containing DBS mutations, either directly called originally by Strelka or originally called by Strelka as two adjacent SNVs and reconstructed as DBS mutations, were created and mutation consequences were re-calculated using VEP as above.

### Patterns of somatic CNA

#### WGD classification

Tumours were classified as WGD considering the average genome copy number state (*ψ*_ave_) as follows:

$${\psi }_{{\rm{ave}}}=\left(\mathop{\sum }\limits_{i=1}^{S}{L}_{i}({C}_{{i}_{{\rm{Maj}}}}+{C}_{{i}_{{\rm{Min}}}})\right)\,/\,\left(\mathop{\sum }\limits_{i=1}^{S}{L}_{i}\right)$$

Where *S* is the number of copy number genome segments, ${C}_{{i}_{{\rm{Maj}}}}$ and ${C}_{{i}_{{\rm{Min}}}}$ are the major and minor allele copy numbers, respectively, for genome segment *i*, and *L*_*i*_ is the base pair length of genome segment *i*. If there was evidence of subclonal alteration, then the copy number states corresponding to the largest tumour cell fraction were considered. Tumours were classified as WGD if 2.9–2*H* < *ψ*_ave_ and non-WGD otherwise, where *H* is the fraction of the genome with a minor allele copy number of 0 (ref. ^32^).

#### Classification of CNAs

Individual CNAs were grouped into six categories: homozygous deletion (HD), LOH, including copy-neutral LOH, other loss (OLOSS), no change (NOC), gain (Gain) and amplification (AMP). The classification considers whether a tumour has undergone WGD (Supplementary Table 38).

For cases in which subclonal CNAs existed, the copy number state corresponding to the largest cell fraction was used. Classification into one of the six categories overlaps significantly between non-WGD and WGD tumours, with differences relating to total copy number. Differences include the following:

- In non-WGD tumours, segments were classified as LOH if 1 allele had a copy number state of 0 and the total copy number (*t*_CN_) ≤2. In WGD tumours, segments were classified as LOH if 1 allele had a copy number state of 0 and *t*_CN_ ≤4.
- Non-WGD tumours do not have an OLOSS category.
- NOC was defined as 1+1 in non-WGD tumours and 2+2 in WGD tumours.
- In non-WGD tumours, segments were classified as Gain if 2 < *t*_CN_ ≤ 5. In WGD tumours, segments were classified as Gain if 4 < *t*_*CN*_ ≤10.
- In non-WGD tumours, segments were classified as AMP if *t*_CN_ > 5. In WGD tumours, segments were classified as AMP if *t*_CN_ > 10.

### Positional enrichment of CNAs

#### Preparing GISTIC input

Recurrent arm-level copy number events, as well as focal amplifications and deletions, were identified using GISTIC (v2.0.2.3)^34^. For all samples with CNA profiles passing quality criteria, a copy number segmentation file suitable for GISTIC input was generated using Battenberg output. Chromosomal coordinates and major (*n*_Maj_) and minor (*n*_Min_) copy number states were obtained for each copy number segment identified by Battenberg. In the case of subclonal copy number segments, *n*_Maj_ and *n*_Min_ values corresponding to the largest tumour cell fraction were considered.

Per-segment normalized copy number (SegCN) values were calculated differently for tumours with WGD (for which ploidy was assumed to be four) and without WGD (for which ploidy was assumed to be two). SegCN was thresholded to a minimum of –2 and maximum of 2.

For non-WGD tumours, SegCN was calculated as follows:

$${\rm{SegCN}}=({n}_{{\rm{Maj}}}+{n}_{{\rm{Min}}})-2$$

For non-WGD tumours from males, X chromosome SegCN was calculated as follows:

$${\rm{SegCN}}=({n}_{{\rm{Maj}}}+{n}_{{\rm{Min}}})-1$$

For WGD tumours, SegCN was calculated as follows:

$${\rm{SegCN}}=(({n}_{{\rm{Maj}}}+{n}_{{\rm{Min}}})-4)/2$$

For WGD tumours from males, X chromosome SegCN was calculated as follows:

$${\rm{SegCN}}=({n}_{{\rm{Maj}}}+{n}_{{\rm{Min}}})-2$$

#### Running GISTIC

GISTIC was run using the following parameters: -conf 0.99 -broad 1 -qvt 0.25 -genegistic 1 -gcm extreme -brlen 0.5 -rx 0 -twoside 1 -scent median -armpeel 1 -arb 1 -refgene hg38.UCSC.add_miR.160920.refgene.mat.

#### Prioritizing probable gene targets of focal amplifications and deletions

Candidate target genes at focal amplifications and deletions were annotated using the following criteria:

1. Overlap with genes at focal amplifications and deletions reported in a previous pan-cancer study that used GISTIC^88^. Comparisons were made both with the overall pan-cancer GISTIC analysis, and GISTIC analysis was restricted to the given tumour type. Special consideration was given to genes specifically highlighted by the previous study^88^ as being candidates.
2. Overlap with Cosmic Cancer Gene Census genes and whether their annotated role (oncogene (OG), TSG or ambiguous) is consistent with the copy number change (OG with amplifications and TSG with deletions)^22^.
3. Overlap with driver genes identified in this study and whether their probable role (OG, TSG or ambiguous) is consistent with the copy number change (OG with amplifications and TSG with deletions).

Based on the above criteria, consensus driver genes were manually assigned to peaks. Comparisons were made with all potential gene synonyms as available from the HUGO gene nomenclature name committee (https://www.genenames.org/).

#### Defining copy number segments overlapping recurrent CNAs

Alterations from the broad analysis with *Q* < 0.05 were taken to indicate recurrent arm-level events. Copy number segments constituting greater than half of the total chromosome arm size were taken to indicate arm-level events.

In the case of focal events identified by GISTIC, the ‘wide region’ was used to compare potential extent of overlap with copy number segments. Segments were defined as overlapping focal events if either the segment interval constituted greater than half of the focal region, or vice versa, using pybedtools and bedtools (v.2.3.0)^89,90^.

Tumours were considered to have specific arm-level or focal deletions if an overlapping copy number segment was annotated as HD or LOH (as described above). Similarly, tumours were considered to have specific arm-level or focal amplifications if an overlapping copy number segment was annotated as Gain or AMP. In the case of subclonal CNAs, *n*_Maj_ and *n*_Min_ values corresponding to the largest cell fractions were considered.

#### ecDNA detection

With the caveat that that there is no definitive way to distinguish ecDNA and intrachromosomal amplification in heterogeneously staining regions, potential ecDNA molecules were detected from tumour bam files using AmpliconArchitect (v.1.2)^29^. In brief, per-tumour seed regions were prepared from Battenberg copy number segmentation output if a segment was >100 kb and the total copy number was >5. AmpliconArchitect was then run using these seed regions to extract overlapping sequence reads from the tumour BAM file and to construct candidate amplicons.

Candidate amplicons were classified using AmpliconClassifier (v.0.4.6) into the following categories: (1) cyclic (truly circularized ecDNA); (2) complex non-cyclic; (3) linear amplification; and (4) no amplification or invalid. Amplicons were highlighted if containing a known highly amplified oncogene (*MDM2*, *MYC*, *EGFR*, *CDK4*, *ERBB2*, *SOX2*, *TERT*, *CCND1*, *E2F3*, *CCNE1*, *CDK6*, *MDM4*, *NEDD9*, *MCL1*, *AKT3*, *BCL2L1*, *ZNF217*, *KRAS*, *PDGFRA*, *AKT1*, *MYCL*, *NKX2-1*, *IGF1R* and *PAX8*, as previously reported^30^).

#### Estimation of telomere content

Telomere content was estimated from tumour and germline BAM files using TelomereHunter (v.1.1.0)^91^ and Telomerecat (v.3.3.0)^92^ with default parameters.

Telomere content was normalized by log_2_(tumour content/normal content).

### Patterns of somatic structural variation

#### Classification of simple and complex SVs

Rearrangements identified by the graph-based consensus approach were grouped into footprints and clusters based on their proximity within the genome, the overall number of events in the genome and the size of these events using ClusterSV^93^. Rearrangement footprints represent sets of rearrangement breakpoints that are positionally associated, whereas rearrangement clusters represent sets of rearrangements that are mechanistically associated. Rearrangement footprints were described using the string approach as previously proposed^93^. Simple and complex events were defined as clusters comprising ≤2 or ≥3 individual rearrangements, respectively. Simple events were classified as deletions, tandem duplications, balanced inversions, balanced translocations or unbalanced translocations, whereas complex events were classified as chromoplexy or chromothripsis (detailed further below). Simple and complex events that did not meet the criteria of any of these classifications were described as simple unclassified or complex unclassified, respectively.

Chromothripsis events were inferred using established criteria^93,94^. A rearrangement cluster was defined as chromothripsis if it met all the following criteria:

- A contiguous series of four genome segments oscillating between two copy number states, or five genome segments oscillating between three copy number states.
- At least six interleaved intrachromosomal rearrangements, as per a previous study^94^.
- No evidence (FDR > 0.2) that the distribution of intrachromosomal fragment join orientations diverge from a multinomial distribution with equal probabilities for each of the four orientation categories (duplication-like, deletion-like, head-to-head inversion and tail-to-tail inversion).

A rearrangement cluster was defined as chromoplexy if it met all the following criteria:

- Contains a chain of rearrangements spanning at least three chromosomes^95^. SV chains were identified using a graph-based approach, in which nodes represent breakpoints, and are connected by an edge if they are not involved in the same rearrangement and fall within 1 Mb of each other. The graph-based approach was implemented using the R package igraph^96^.
- At least 50% of rearrangement footprints in the cluster represent balanced translocations, either with no observed copy number change or a deletion bridge between the break ends.
- Consists of between 3 and 30 rearrangements.

#### Identification of simple structural variation hotspots

Rates of somatic structural variation differ throughout the genome and are influenced by local genomic features^97^. Genome regions enriched for simple SVs (Supplementary Table 10) were therefore identified using a permutation-based approach considering genomic features associated with structural variation occurrence. Deletions, tandem duplications, balanced inversions, balanced interchromosomal translocations and unclassified simple SVs were considered separately. Individual rearrangements forming parts of complex SVs were excluded from this analysis. MSS primary and MSI tumours were also analysed separately, whereas primary POL tumours and metastases were not considered owing to low sample numbers.

#### Evaluating relationships between genomic features and SV frequencies

Negative binomial regression was used to test associations between genomic features and numbers of SVs of each simple class^97^. The following features were included in the models: average total copy number across the bin in the CRC sample set, GC content, the presence of genes highly or lowly expressed in CRC, ALU repeats, other genomic repeats, segmental duplications, fragile sites, replication timing, and DNase, H3K36me3 and H3K9me3 peaks. Highly and lowly expressed genes were defined as those with mean RSEM value in the top 25% and bottom 75% of protein-coding genes in TCGA CRC samples with RNA sequencing^7^. ALU and other genomic repeats were obtained from the UCSC Genome Browser^98^. Segmental duplications were obtained for GRCh38 from the Segmental Duplication Database^99^. Fragile sites were obtained from a previous study^66^. Replication timing data from CRC epithelial cells (HCT116) were obtained from ReplicationDomain^100^. DNase-seq data (ENCFF443KCU) and ChIP–seq data for histones H3K36me3 (ENCFF553QXG) and H3K9me3 (ENCFF482DLD) were obtained for the large intestine from ENCODE^101^.

#### Permuting SVs

SVs were simulated to test whether the number of SVs observed in a region was greater than expected by chance given the local genomic features^102^. SVs were simulated for each simple SV class, preserving the number and length (distance between intrachromosomal SV break ends) of SVs observed in the CRC sample sets. To simulate SVs, the genome was divided into non-overlapping 1 Mb bins and the genomic features (listed above) of each bin summarized. All genomic features were normalized to a mean of 0 and standard deviation of 1 to aid comparisons. The number of break ends expected in each bin was then estimated using the effect estimates from the previously generated negative binomial regression model. For each observed SV, a SV was simulated by sampling a bin under probabilities proportional to the expected numbers of break ends in each bin. For intrachromosomal SVs, a partner break end was then simulated by selecting the position either upstream or downstream (with equal probability) equal in distance to the distance between the two break ends in the observed SV. For interchromosomal SVs, a partner break end was simulated by sampling a bin under probabilities proportional to the expected numbers of break ends in each bin, excluding bins on the same chromosome. SVs were re-simulated if either break end fell within an uncallable region (a telomere or centromere). SVs were simulated 1,000 times to generate a null distribution of expected SV numbers for the 1-Mb bins.

#### Identifying SV hotspots

Piece-wise constant fitting (PCF) was used to identify regions of the genome containing greater numbers of SV break ends than expected^102^. SV break ends were first sorted by position and the distance between successive break ends calculated. PCF was then applied to the log_10_ of these inter-mutational distances (IMDs). SV hotspots were identified by first computing the observed (*d*^obs^_*i*_) and expected (*d*^exp^_i_) number of breakends per base pair for each PCF segment (*i*):

$$\begin{array}{c}{d}_{i}^{{\rm{obs}}}={a}_{i}/{s}_{i}\\ {d}_{i}^{{{\exp }}}=\left(\mathop{\sum }\limits_{j=1}^{n}{b}_{j}\right)/n{s}^{{\rm{bin}}}\end{array}$$

Where *a*_*i*_ is the number of break ends in the segment, *s*_*i*_ is the length of the segment in base pairs, *n* is the number of bins overlapping the segment, *b*_*j*_ is the expected number of SVs in bin *j*, and *s*^bin^ is the bin size (1 Mb). A simple SV enrichment factor *β*_*i*_^simple^ is then computed for each PCF segment as follows:

$${{\beta }_{i}}^{{\rm{simple}}}={d}_{i}^{{\rm{obs}}}/{d}_{i}^{{{\exp }}}$$

The PCF algorithm requires parameters *γ* (that controls the smoothness of the segmentation) and *k*_min_ (the minimum number of mutations in a segment). FDRs at each *β*^simple^ value were estimated by applying PCF to both the observed and simulated SV sets and dividing the mean number of segments with a *β*^simple^ value at least as great in the simulated SV sets by the number of segments with a *β*^simple^ value at least as great in the observed SV set. A maximum FDR of one was set and FDR values equal to zero were changed to the lowest non-zero FDR value observed. Optimal *γ* and *k*_min_ values were chosen by repeating this process for values of *γ* between 1 and 20, and values of *k*_min_ between 2 and 20, and selecting values that maximized the number of hotspots identified while minimizing the FDR. In the final analysis, *γ* = 10 was used throughout, whereas *k*_min_ = 2 was used for translocations in MSS primary samples, *k*_min_ = 4 was used for unclassified simple variants in primary MSI samples, and *k*_min_ = 10 was used otherwise. SV hotspots for which no SVs were supported by CNAs were considered potential artefacts and removed. Overlapping SV hotspots identified in the same sample sets were collapsed.

#### Classification of SV hotspots as fragile sites

SV hotspots were classified as fragile sites if they satisfied at least three of the following six criteria (this threshold was chosen by assessing the co-occurrence of these criteria):

- Were late replicating^103^. Replication timing data from CRC epithelial cells (HCT116) were obtained from ReplicationDomain. Late-replicating regions were defined as those with mean Repli-Seq values ≤ 0.
- Had low gene density^104^. A threshold of five genes per megabase was used.
- Overlapped a gene greater than 300 kb in size. This threshold was chosen as fragile sites generally occur in chromosome regions containing genes at least 300 kb in size^105^.
- The overlapping gene of greatest size was the focus of the SV enrichment. This was assessed by computing the ratio between SV break point densities in the overlapping gene of greatest size and intergenic regions flanking 1 Mb upstream and downstream. A threshold of five was used.
- Overlapped a fragile site as previously reported^66^. These fragile sites were originally obtained from either the NCBI or literature curation and were mapped from NCRI36 to GRCh38 co-ordinates using LiftOver^98^.
- Overlapped a fragile site identified in a pan-cancer analysis of whole-genome-sequenced tumours^102^ and mapped from GRCh37 to GRCh38 co-ordinates using LiftOver^98^.

SV hotspots were not considered as potential fragile sites if they contained an identified CRC driver gene. SV hotspots at potential fragile sites likely occur for mechanistic rather than selective reasons and were therefore not considered further^66^.

#### Identification of candidate gene targets of recurrent SVs

Genes were reported as candidate targets of recurrent SVs if they had been identified as targets in previously analyses^4,7,8,102^, were known CRC driver genes overlapping an SV hotspot or were the sole expressed gene in the hotspot region. Numbers of samples with a focal change at a candidate gene were computed considering SVs <3 Mb in size^106^ at least partially overlapping the gene coding sequence.

#### Enrichment of complex structural variation

Genome regions enriched for complex SVs were identified using a permutation-based approach, considering chromothripsis, chromoplexy and unclassified complex SVs separately. MSS primary and MSI tumours were also analysed separately, whereas POL tumours and were not considered owing to low sample numbers. The genome was first split into non-overlapping 1 Mb bins and the observed number of tumour samples with complex SV footprints (*g*^pbs^_*i*_) overlapping each bin (*j*) counted. Complex SV footprint positions were next permuted 100,000 times by randomly sampling genome regions equal in size to the footprints. The expected number of tumour samples with complex SV footprints (*g*^exp^_*i*_) overlapping each 100-kb bin was then estimated as the mean number of tumour samples with SV footprints overlapping the bin across all permutations. A complex SV enrichment factor *β*_*i*_^complex^ was calculated for bin (*j*) as follows:

$${{\beta }_{i}}^{{\rm{complex}}}={g}_{i}^{{\rm{obs}}}/{g}_{i}^{{{\exp }}}$$

FDRs at each *β*^complex^ value were estimated by computing *β*^complex^ for each bin in both the observed and permuted SV sets and dividing the mean number of bins with a *β*^complex^ value at least as great in the permuted SV sets by the number of bins with a *β*^complex^ value at least as great in the observed SV set. A maximum FDR of 1 was set and FDR values equal to zero were changed to the lowest non-zero FDR value.

### Mutational processes

#### Characterizing SBS, DBS and indel signatures

SBS, DBS and indel signatures were extracted de novo and related to known COSMIC signatures (v.3.2) using SigProfilerExtractor^11^. SBS, DBS and indel signatures were extracted using random initialization, 500 NMF replicates, and between 10,000 and 1,000,000 NMF iterations. We assumed the presence of between 1 and 30 SBS signatures (minimum signatures and maximum signatures parameters, respectively), 1 and 15 DBS signatures, and 1 and 10 indel signatures. Default settings were used for all other parameters. Investigation of the new DBS-A signature (Supplementary Table 3) hinted towards the signature being a technical artefact of the high number of short indels at homopolymer regions occurring in MSI samples.

#### Characterizing SV signatures

SV signatures were extracted considering only simple SVs, specifically deletions, tandem duplications, balanced and unbalanced inversions, and balanced and unbalanced interchromosomal translocations. Deletion and tandem duplication size distributions are multimodal, and we therefore classified these variants as <10 kb, 10 kb to 1 Mb, and > 1Mb. Variant site replication timing is also multimodal and we therefore classified variants as late, mid or early replicating considering mean Repli-Seq thresholds of <–2, –2 to 2, and >2 using CRC epithelial cell data from ReplicationDomain (Supplementary Fig. 8).

Mechanisms of fragile site instability differ from other SVs, and deletions and tandem duplications at fragile sites were therefore considered separately^91^. Signatures were extracted using a hierarchical Dirichlet process (HDP) implemented in the R package hdp (v.0.1.5)^91^. The hierarchical Dirichlet process structure was initialized with one common grandparent node, a parent node for each of the MSS, MSI and POL tumour subtypes, and a child node for each of the 1,765 tumour samples in which SVs were called. Four separate Markov chain Monte Carlo posterior sampling chains were run with 5,000 burn-in iterations, extracting 12 SV signatures. Extraction stability was assessed by splitting the cohort into halves, maintaining proportions of MSS, MSI and POL tumours, and re-extracting signatures from each half. Nine signatures extracted from the cohort halves showed high similarity between halves (cosine similarity > 0.9) and high similarity with signatures extracted from the full cohort. These nine signatures were named SV1–SV9 and considered in subsequent analyses.

To investigate DNA repair mechanism perturbation, we correlated driver gene mutation with SV signature activity. A gene was considered mutated if it harboured a likely pathogenic germline SNP or indel (variants annotated as ‘pathogenic’ or ‘likely pathogenic’ in ClinVar^107^), a likely oncogenic somatic SNV or indel, or a homozygous deletion at a gene exon. Pairwise associations between gene mutation and SV signature activity were tested for using multiple linear regression, including gene mutation status, age at sampling, primary tumour site and tumour sample purity as independent variables. Genes were considered if they were mutated in at least 1% of tumours. *TP53* mutation is associated with increased CIN, and *TP53* was therefore included in all models. The Yeo–Johnson extension to the Box–Cox transformation was applied to mutation numbers to reduce heteroscedacity and to ensure distributions were approximately normal^108^. Samples with missing independent variable values were excluded. Owing to mutational burden heterogeneity, only MSS primary tumours were considered in this analysis. *P* values were adjusted for multiple testing using Bonferroni correction and a threshold of *P* = 0.05 considered significant.

#### Characterizing copy number signatures

SigProfilerExtractor was used to extract copy number (CN) signatures in the 1,765 tumours with profiled CNAs^12^. Where Battenberg identified a subclonal CNA, the copy number states corresponding to the largest tumour cell fraction were used, as SigProfilerExtractor cannot consider subclonal copy number states. Each copy number segment was assigned to 1 of 48 categories using SigProfilerMatrixGenerator, considering heterozygous or homozygous state, total copy number and segment length^12,17^. Combinations of 1–30 de novo signatures were extracted and the recommended solution was accepted, balancing cosine distance with average stability (Supplementary Fig. 9), with the selection plot showing the mean sample cosine difference and average stability for de novo extraction of 1–30 CN signature. The accepted solution contained four de novo signatures.

De novo CN signatures were then deconvolved into their matching component COSMIC CN signatures from COSMIC (v.3) to identify six contributing COSMIC signatures, as shown below (CN1 (near-diploid state); CN2 (genome doubling); CN6 (chromothripsis/amplification with WGD); CN9 (CIN without WGD); CN17 (chromosomal-scale LOH); and CN20 (unknown aetiology)). CNV48A is a heterogeneous signature, dominated by heterozygous segments of 3–8 copies. It is decomposed into three COSMIC signatures: CN17, associated with HRD and TD (42.18%); CN6, associated with chromothripsis (29/72%); and CN20, which has a currently unexplained aetiology (28.1%). CNV48B is comprised primarily of heterozygous segments of 3-4 copies with a length of >40 Mb it is deconvoluted into a single cosmic signature CN2, associated with tetraploidy. CNV48C is dominated by heterozygous segments with a copy number of 2 and is decomposed to CN1, indicative of a diploid state. CNV48D is dominated by LOH segments with a copy number of 1 and heterozygous segments with a copy number of 2 and to a lesser extent 3–4, it deconvoluted into CN9, which has previously been associated with chromosomally unstable diploid tumours.

Each CN signature was assigned as being active or inactive in each sample. Associations with MSI status, ploidy and HRD status were calculated using Fisher’s exact test, comparing samples with and without the phenotype with those that had or did not have the active signature.

#### Predicting HRD

Evidence of HRD was assessed using HRDetect^109^. HRDetect considers six genomic features predictive of HRD: (1) proportion of deletions with microhomology, (2) SBS3 contribution, (3) SBS8 contribution, (4) rearrangement signature RS3 contribution, (5) rearrangement signature RS5 contribution and (6) HRD index. HRDetect requires CNA data and was therefore run only on the 1,765 out of 2,023 tumours passing CNA calling. SBS3 and SBS8 contribution estimates were obtained from SigProfiler. Rearrangement signatures RS3 and RS5 were computed using HRDetect, using a previously reported rearrangement signature^73^. Although HRDetect was trained on breast cancers, it has demonstrated high efficacy when applied to other cancer types^109^. It was not possible to retrain HRDetect using our CRC samples, as few tumours exhibited a pathogenic germline *BRCA1* or *BRCA2* variant with somatic loss of heterozygosity of the wild-type allele.

### Pathway analysis

#### Analysis of disrupted pathways

Altered pathways were identified by integrating coding and noncoding mutations using ActivePathways^110^. MSS, MSI and POL cancers were considered separately. Six mutation features were used: coding driver *P* values from IntOGen^18^ and 3′ UTR, 5′ UTR, core promoter, distal promoter and non-canonical splice site *P* values from OncodriveFML^78^. We tested Reactome pathways obtained from MSigDB^111^. All protein-coding genes included in at least one Reactome pathway were considered as the background gene set.

#### Driver mutation co-occurrence

Simple methods such as Fisher’s exact test and multiple regression were used to assess pairwise co-occurrence of driver genes. As these methods assume a null in which the probability of a gene alteration is independent of another gene, we also investigated use of the DISCOVER algorithm^112^, which accounts for mutational heterogeneity at both the gene and tumour level. In practice, we reported simple association statistics, as we wished to include positively or negatively co-occurring driver genes or mutations, irrespective of a shared aetiology (for example, both genes containing short repeats prone to small indels in MSI tumours).

#### Cluster analysis

To search for groups of tumours with similar features, we used consensus clustering^113,114^. We clustered 1,471 primary, treatment-naive tumours with CNA data using the following 304 clinical and molecular features: SNV, indel, SV and CNA burdens; all SBS, DBS, ID, SV and CN signature burdens; binary presence of mutations in 196 driver genes; ploidy; WGD status; fraction of the genome with LOH; mean ploidy across each chromosome arm divided by total ploidy (excluding the short arms of acrocentric chromosomes); age at sampling; sex; and subtype.

The features were ranked and normalized such that the resulting values were between zero and one. Hierarchical agglomerative clustering was run on these features using the diceR R package with the following distance metrics and linkage criteria:

- Distance metrics: Euclidean, Manhattan, cosine, correlation, Jaccard, eJaccard and fJaccard (from the R package proxy).
- Linkage criteria: average, complete, median, mcquitty, ward.D and ward.D2 (from R’s hclust function).

Each combination of distance metric and linkage criterion was run 10 times on random samples of 80% of the tumours. The number of clusters was varied from two to ten. We looked for robust clustering using the following criteria:

- The clustering must closely recapitulate the MSS, MSI, and POL subtypes
- High average clustering consensus^114^
- Absence of tiny clusters (<5 samples)

The ward.D2 linkage^115,116^ consistently performed better than the other linkage criteria. With this linkage, Euclidean and Manhattan distances gave good clustering, but we chose Euclidean because the Manhattan distance failed to reproduce the POL subtype when the number of clusters was greater than six.

To increase the robustness of the clustering, we removed tumours that had an item consensus <0.7 and re-clustered using the resulting consensus matrix. This step removed 471 tumours that were difficult to cluster consistently and led to an increase in mean cluster consensus from 0.77 to 0.91. Following these steps, all samples had their subtype correctly classified, except for two MSI samples misclassified as MSS.

### Immune profiling

#### HLA haplotyping

HLA typing of blood-derived normal samples was conducted using HLATyper, which is part of the Illumina Whole Genome Sequencing Service Informatic pipeline. The highest-ranking allele pair prediction for each type-I HLA allele (A, B and C) was taken to define a six-allele HLA set for each case.

#### Immune-escape prediction

We predicted three separate mechanisms of immune escape: (1) HLA gene mutation; (2) HLA gene LOH; and (3) mutation and LOH of other APGs.

Somatic mutations in the HLA locus were predicted using POLYSOLVER^117^. First, alleles were converted to a POLYSOLVER-compatible format (lower case, digits separated by underscore) and outputted into a patient-specific winners.hla.txt file. Next, the POLYSOLVER mutation-detection script (shell_call_hla_mutations_from_type) was run on matched tumour–normal pairs to call tumour-specific alterations in HLA-aligned sequencing reads using MuTect^118^. Strelka (v.2.9.9)^70^ was also run to detect short insertions and deletions in HLA-aligned reads, as it offers increased sensitivity over POLYSOLVER’s default caller. Finally, both SNVs and indels passing quality control were annotated with POLYSOLVER’s annotation script (shell_annotate_hla_mutations).

LOH at the HLA locus was predicted using LOHHLA^119^. The same winners.hla.txt files were used as input, with POLYSOLVER’s comprehensive deduplicated FASTA of HLA haplotype sequences as reference. A type-I allele of a patient was annotated as allelic imbalance (AI) if the *P* value corresponding to the difference in evidence for the two alleles was <0.01. Alleles with AI were further labelled as LOH if the following criteria held: (1) the predicted copy number of the lost allele was <0.50 with CI < 0.70; (2) the copy number of the kept allele was >0.75; and (3) the number of mismatched sites between alleles was >10.

We also evaluated somatic mutations and copy number status of the following APGs^120^: *B2M*, *CALR*, *CANX*, *CIITA*, *ERAP1*, *ERAP2*, *HSPBP1*, *IRF1*, *PDIA3*, *PSMA7*, *PSME1*, *PSME2*, *PSME3*, *TAP1* and *TAP2*. First, somatic mutations were annotated using ANNOVAR^121^. An APG was deemed mutated if it contained any non-synonymous, frameshift, stop-loss, or stop-gain mutation in its exons. The copy number status of each gene was evaluated using Battenberg output.

A sample was defined as immune escaped if it showed at least one of the following: (1) HLA mutation; (2) HLA LOH; or (3) APG mutation. HLA AI was not considered to provide immune escape as AI can arise from multiple sources (including subclonal LOH and unequal focal gains of the locus) and therefore the effect of AI on antigen presentation is uncertain. For cases when HLA alterations could not be fully evaluated (see ‘Sample subsetting and statistical analysis’ below), but no HLA or APG alteration was detected, the immune escape status was considered unknown as we could not eliminate the possibility of immune escape.

#### Neoantigen prediction

We predicted neoantigens using NeoPredPipe, a Python-based pipeline combining ANNOVAR and netMHCpan (v.4.0)^122–124^. In brief, all somatic SNVs and indels were annotated using ANNOVAR and for all non-synonymous exonic mutations the mutated peptide sequence was predicted. We took any 9- and 10-mer spanning the mutated amino acid (or acids), resulting in either (1) a 19-amino acid window for SNVs or (2) a peptide until the next predicted stop codon for frameshift mutations. These peptides were evaluated according to their novelty and predicted binding strength to the patient’s six-allele HLA set comprised of the *HLA-A*, *HLA-B* and *HLA-C* genes. Peptides that were new compared with the healthy human proteome with binding rank of two or below (among the best 2% of binders compared with a set of random peptides) were reported as neoantigens. All patient-specific HLA alleles were used for neoantigen prediction, regardless of mutation or LOH status of the HLA locus.

We considered a mutation neoantigenic if at least one of its downstream mutated peptides was a neoantigen with respect to any of the patient’s six HLA alleles. We defined neoantigen burden as the total number of neoantigenic mutations in the sample. We also evaluated the following alternative measures: (1) number of peptide–HLA binding pairs; (2) number of strong binder (best 0.5% of peptides) peptide–HLA binding pairs; (3) number of neoantigenic mutations in genes expressed in CRC (expression ≥10 TPM in ≥10% of TCGA CRCs)^7^. We found that all these measures were highly correlated with our definition of neoantigen burden: (1) *R* = 0.993, (2) *R* = 0.989, (3) 0.983; *P* < 10^–16^ for all (Supplementary Fig. 10).

#### Sample subsetting and statistical analysis

Eighty-five samples were excluded from neoantigen calling because netMHCpan was unable to predict at least one of their HLA haplotypes. Overall, 217 samples had 1 or more haplotypes incompatible with POLYSOLVER, for which HLA mutation and LOH calling was restricted to the compatible haplotypes (1, 2 and 3 haplotypes were excluded in 171, 37 and 9 samples, respectively). In addition, LOH was not considered for 15 patients because they were homozygous for all type-I HLA genes. In total, 1,744 out of 2,023 samples had complete neoantigen and HLA alteration information available.

As CRC subtypes (MSS, MSI and POL) have substantially different mutation and immune properties, all analyses were completed separately for each subtype. Pairwise comparisons were conducted using Wilcoxon tests. Analysis of immune differences associated with tumour site was restricted to MSS primary samples, with samples that lacked specific information (site information missing or only specified as ‘colon’) excluded, leaving *n* = 1,100 samples.

Multivariate regression between immune escape types and neoantigen burden was performed using the lm function against the logarithm of neoantigen burden and therefore defined the fold change in burden associated with each escape type. Multivariate regression, including clinical characteristics, was carried out similarly, using the logarithm of total mutation burden as an additional independent variable. The number of POL samples was insufficient for statistical analysis and the regression analyses were therefore only conducted for MSS and MSI tumours.

#### PHBR analysis

We computed the immunogenicity of a given mutation in a given patient using PPHBR^40^, which takes into account all novel peptides produced by that mutation and all HLA alleles present in the patient. Low PHBR values correspond to mutations that are likely to be presented on the cell surface and hence with a high immunogenic potential, whereas high PHBR mutations are less immunogenic. The overall immunogenic potential of a mutation within a cohort is defined as the median of PHBR values within that cohort. For each mutation and HLA haplotype pair considered, we generated all 8–11-mers overlapping the mutation and evaluated their binding affinity to the HLA allele using the ‘all-predictions’ mode of NeoPredPipe. The best (lowest) rank was recorded. For a given patient, PHBR were computed as the harmonic mean of six best rank values corresponding to the patient’s six HLA haplotypes (homozygous alleles were counted twice). We computed PHBR values for all single nucleotide mutations located in driver genes that were present in at least four cancers in the cohort. The 85 samples with incompatible HLA alleles were excluded.

To evaluate the effect of HLA alterations on PHBR values, we repeated the same analysis for affected patients with a reduced set (<6) of HLA alleles that were unaltered. To measure the level of patient- (HLA-) dependent selection on driver genes, we compared PHBR values for mutations in these genes between patients that did not carry the mutation and patients that did. Negative values indicate that mutations of the gene are enriched in patients for whom they have lower immunogenic potential. PHBR values between patients with no mutations and patients with mutations were compared using Wilcoxon rank-test, and *P* values were adjusted for multiple testing using Benjamini–Hochberg correction.

For comparisons such as those shown in Extended Data Fig. 7, the immunogenic potential of individual mutations was quantified using the median of PHBR values associated with that single nucleotide change for each patient belonging to a specific cohort or subcohort. Immunogenicity of groups of driver genes (for example, metastasis-specific drivers) was evaluated by considering all mutations observed in the genes and median PHBR computed across the entire cohort of CRCs or MSS primary CRCs, as indicated. Values for an individual mutation across different cohorts were compared using paired Wilcoxon rank-tests.

### Mitochondrial genome characterization

#### Calling mitochondrial somatic SNVs and indels

Somatic mitochondrial SNVs and indels were called using Mutect2 (v.4.1.4.1)^125^, with the light strand as reference based on the human mtDNA revised Cambridge reference sequence (rCRS). Somatic mitochondrial variants were excluded if they had the following:

- Low mapping quality score (<20).
- Low base quality score (<20).
- An alternative allele frequency <1%.
- Missing alternative reads in any stand direction.
- Location within hypermutated regions (302–316, 514–525 or 3106–3109).

Mutational distributions of SNVs, categorized by the six possible pyrimidine substitution classes, were constructed to analyse mutational processes. Distributions of substitutions on the D-loop, including and excluding variants between the two origins of replication (${\text{O}}_{H}$ and Ori-b, between sites 16,197 and 191) were also analysed by substitution class^65^. Pathogenic variants were identified using ClinVar^107^, considering annotations where at least one submitter provided an ‘interpretation with assertion criteria and evidence’.

#### Mitochondrial copy number estimation

Autosomal and mitochondrial genome coverage was computed using fastMitoCalc^126^. Using estimated sample purity ($\rho$), tumour ploidy ($\varphi$) and mean coverage depth, tumour sample mitochondrial DNA copy number was estimated as previously described^31^:

$$\begin{array}{l}{\rm{Tumour}}\,{\rm{sample}}\,{\rm{mtDNA}}\,{\rm{copy}}\,{\rm{number}}\\ \,=\,({\rm{mtDNA}}\,{\rm{mean}}\,{\rm{coverage}})/({\rm{autosomal}}\,{\rm{DNA}}\,{\rm{mean}}\,{\rm{coverage}})(\,\rho \varphi +2(1-\rho ))\end{array}$$

Mitochondrial copy number was estimated for only the 1,765 out of 2,023 tumours that passed CNA calling and therefore had purity and tumour ploidy estimates.

Linear regression was used to correlate mtDNA copy number with age at sampling, tumour stage, site of primary tumour, sex and tumour purity. The Yeo–Johnson extension of the Box–Cox transformation was applied to mtDNA copy number. Linear regression was applied considering all tumours and segregating MSS and MSI tumours. Regression results were adjusted for multiple testing using the Benjamini–Hochberg procedure.

#### Selection of mitochondrial mutation and *POLG* correlation

For the 13 mitochondrial protein-coding genes, selective pressure was quantified by calculating the respective dN/dS values using the R package dNdScv, with non-mtDNA chromosomes removed from the reference genome^6^. A global mitochondrial dN/dS value was also estimated, excluding *MT-ND6* due to a suspected replication bias. Results were adjusted for multiple testing using the Benjamini–Hochberg procedure. In addition, it was investigated whether *POLG* mutations resulted in altered mitochondria mutational burden compared to other tumours. Only the primary MSI cohort was analysed for this trait, as other subcohorts had too few tumours with non-synonymous *POLG* mutations.

### Genomic impact of previous treatments

Whether individuals had received systemic treatment or colorectum-targeting radiotherapy before sampling was based on data from NHSD and PHE-NCRAS. For NHSD, records related to systematic treatment were obtained from the Admitted Patient Care and Outpatients tables using associated Office of Population Censuses and Surveys (OPCS)-4 codes. For PHE-NCRAS, records related to systemic treatment were obtained from the AV_TREATMENT table using the event description codes, and from the Systemic Anti-Cancer Therapy (SACT) table. For PHE-NCRAS, records related to radiotherapy were obtained from the AV_TREATMENT table using the event description codes, and from the National Radiotherapy Dataset (RTDS) table considering records associated with a CRC diagnosis.

In total, 315 participants received systemic treatment or radiotherapy before tumour sampling. A total of 278 participants received systemic therapy before CRC sampling for sequencing, and information on the drugs administered was available for 182 of these participants. For 253 participants, the systemic treatment was used to treat CRC, whereas for 25 participants, it was used previously to treat another cancer. Overall, 94 participants received capecitabine, 23 received cetuximab, 93 received fluorouracil, 39 received irinotecan, 109 received oxaliplatin, 46 received steroids and 28 received other drugs. In total, 118 participants received colorectum-targeted radiotherapy before tumour sampling.

Associations between systemic treatment and colorectum-targeting radiotherapy before sampling with mutational signature activity were tested using multiple logistic regression. Previous treatment with radiotherapy, capecitabine, cetuximab, fluorouracil, irinotecan, oxaliplatin and steroids was included in the models as binary independent variants. Other treatments administered before sampling occurred in fewer than five individuals and were therefore not included in the models. One model was created for each of the identified SBS, ID, DBS and SV signatures, with signature presence encoded as a binary dependent variable based on whether any evidence of the signature was identified in each sample. In total, 96 samples that received treatment before sampling, but for which the specific administered drugs were unknown, were not included. Both primary tumours and metastases were considered in these analyses. Treatment coefficient *P* values were adjusted for multiple testing using Bonferroni correction and a threshold of *P* = 0.05, considered significant. Treatment duration was measured as the time between the first and last treatment administration.

### Metastasis-specific analyses

Tumours were split between primary (*n* = 1,354) and metastatic (*n* = 105) MSS samples. Only MSS samples were included as there was just one MSI metastasis and no POL metastasis. Five primary tumours were matched to metastasis samples in this cohort, but for the purposes of the analysis all samples were treated as unmatched. To determine mutational burden, VCF files were filtered for PASS variants and the number of SNVs and indels summed. These were then divided by the total genome length (3,088.27 Mb). For the binned copy number analysis, the genome was first partitioned into 2,766 1 Mb windows. For each sample, the absolute allele-specific copy number within each bin was recorded. If two copy number segments overlapped a bin, the copy number of the segment with the larger overlap was recorded. Copy numbers were then classified according to the section ‘Classification of CNAs’. For each aberration type (gain or deletion/LOH) the proportion of primary tumours with that aberration was compared to the proportion of metastatic samples with two-sided Fisher’s exact tests. The difference between the proportions was then plotted as a trace along the genome with stars indicating significantly different bins. *P* values were corrected for multiple testing (FDR < 0.05). Absolute copy number calls were divided by mean integer ploidy to account for differences in ploidy between the two groups. The adjusted copy numbers for each bin were then compared between primaries and metastases using Wilcoxon signed-rank tests while correcting for multiple testing (FDR < 0.05). The difference in the mean (ploidy-adjusted) copy number was then plotted as a trace along the genome, with stars indicating significant bins.

### Microbiome

#### Microbial identification

Microbial sequences^126^ were identified using GATK PathSeq^127^ aligned against the default PathSeq microbial genome bundles. A minimum clipped read length of 60 bp was used with all other parameters set to their defaults. Unambiguously assigned reads were used for the decontamination steps. Thereafter the adjusted score output was used, sharing ambiguous reads between species. Score output for each sample was converted to microbial cells per human cell for each taxon by adjusting for microbial and human average genome size (average human genome calculated from copy number and tumour cell percentage data).

$${\rm{Microbial}}\,{\rm{cells}}\,{\rm{per}}\,{\rm{human}}\,{\rm{cell}}=\frac{({\rm{Microbial}}\,{\rm{reads}})/({\rm{Average}}\,{\rm{microbial}}\,{\rm{genome}}\,{\rm{size}})}{({\rm{Human}}\,{\rm{reads}})/({\rm{Average}}\,{\rm{human}}\,{\rm{genome}}\,{\rm{size}})}$$

This analysis showed that metastases had extremely low microbial content and therefore subsequent steps included only primary tumours unless otherwise stated. Reads passing PathSeq filters were realigned against the *E.* *coli* colibactin gene cluster^128^ using bwa^102^, and matching reads counted.

#### Contaminants

Potential contaminant species were identified using methods developed by The Cancer Microbiome Atlas^129^. In brief, the prevalence of species found in primary tumours and matched blood was compared (Extended Data Fig. 8a). Samples were called as positive for a species if two or more unambiguously aligned reads from the species was found. Species were deemed as probable tumour sample origin if a Fisher one-sided exact test found them to be more prevalent in the tumour sample than the blood sample (FDR < 0.05) and blood sample prevalence was <20% of samples. Genus level scores were recalculated from species scores by only including the species scores that survived this decontamination step. To mitigate the effects of species with mixed biological and contaminant components^130^, downstream steps were adjusted for NHS Hospital Trust where possible (see below) as the processing laboratory was a plausible source of contamination.

#### Identifying taxa associated with CRC

CRC-associated taxa were identified by pooling all species level read numbers from eight published stool metagenomic studies^2,131–134^. Application of LEfSe to these data identified 73 species and 37 genera associated with CRC^135^. Bacterial species were classified as oral microbes if they were identified as ‘oral taxon’ or ‘oral species’ by PathSeq or if they were present in the expanded Human Oral Microbe Database^136^.

#### Comparing microbiome and clinicopathological data

Microbial relative abundances were compared to clinicopathological data using decontaminated PathSeq output. Only tumours with complete data for the relevant categories were included in each comparison. Genus and species level alpha diversity was measured using the Shannon index and beta diversity using Bray–Curtis dissimilarity of relative abundance. Differences in beta diversity were measured by PERMANOVA using the adonis function^137^ in Vegan using default settings, with permutations confined to within NHS Trusts using the ‘strata’ setting to minimize cross-site contamination differences. Taxa differing between clinicopathological categories were measured using MaAsLin2^138^, with minimum abundance of 0, minimum prevalence of 0.1, and NHS Trust added as a random effect to minimize cross-site contamination differences.

### Statistical analysis and clinicopathological correlates

Statistical tests were two-sided and unpaired unless otherwise stated. Fisher’s exact and *χ*^2^ tests were used for categorical variables. Wilcoxon (rank-sum) tests, *t*-tests and Kruskal–Wallis tests were used for quantitative variables. Multivariable analyses are described below.

#### Correlating variables

Multiple linear regression was used to investigate the relationship between clinicopathological features and numbers of SNVs, indels, CNAs and SVs, and numbers of mutations attributed to SBS, ID, DBS and SV signatures. Number of CNAs was defined as the number of genome segments for which the clonal or subclonal copy number state was not 1:1 in non-WGD tumours or was not 2:2 in WGD tumours.

Multiple logistic regression was used to investigate the relationship between the presence or absence of clinicopathological features and driver gene mutation, recurrent arm-level CNAs, recurrent focal CNAs, WGD and evidence of CN signatures. Unlike SBS, ID, DBS and SV signatures, the activities of CN signatures do not represent numbers of mutations attributed to the signature^12^. We primarily considered the presence or absence of CN signatures, but also assessed measures of activity or burdens where stated.

MSS primary and primary MSI tumours were considered separately. Signatures were tested if they were identified in at least 1% of the tumour set, driver genes were considered if they were mutated in at least 5% of the tumour set, and arm-level and focal copy number alterations were considered if identified as recurrent by GISTIC. *TP53* mutation is associated with increased CIN, and *TP53* somatic mutation status was therefore included in mutation number models. Considering multiple variables together in a single model is essential given that many of these variables are correlated, including age, primary tumour site and stage. The Yeo–Johnson extension to the Box–Cox transformation was applied to mutation numbers to reduce heteroscedacity and to ensure distributions were approximately normal^108^. Samples with missing independent variable values were excluded. Primary tumour site and tumour stage were considered as ordinal variables. Primary tumour site was encoded as a single ordinal variable with the following values: caecum = 1; ascending colon = 2; hepatic flexure = 3; transverse colon = 4; splenic flexure = 5; descending colon = 6; sigmoid colon = 7, rectosigmoid junction = 8; rectum = 9. Exploratory analyses with location as a binary variable (proximal versus distal colorectum) or ternary variable (proximal colon, distal colon, rectum) were also performed in some cases (Extended Data Fig. 9b). Tumour stage was also encoded a single ordinal variable with values corresponding to the four Dukes stages. Unless otherwise stated, for each individual variable, *P* values were adjusted for multiple testing using Bonferroni correction and a threshold of *P* = 0.05 considered significant.

#### Survival analysis

Correlation of clinicopathological and genomic variables with all-cause mortality (overall survival) was assessed using Cox proportional hazards models. Follow-up time was measured from the date that the tumour was sampled (as a proxy for date of presentation or diagnosis) to the corresponding patient’s most recent time of contact. The median follow-up time was 1,075 days. Only individuals for whom the primary tumour was sequenced were included. To avoid proportional hazards assumption violation, individuals with MSS and MSI tumours were considered separately. Individuals were excluded if tumour sampling occurred before 1 January 2015 or the time between CRC diagnosis and tumour sampling was greater than 1 year. Hazard ratios were adjusted for sex, patient age at sampling, primary tumour location and Dukes stage. Owing to small numbers of deaths, Dukes stages A and B were combined. Analyses were performed regarding location as a binary variable (proximal versus distal colorectum) and as an ordinal variable (locations 1–9 from caecum to rectum).

After excluding individuals with missing covariate data, the MSS and MSI cohorts comprised 836 (144 deaths) and 272 (48 deaths) individuals. The following variables were analysed:

- Total mutational burden (SNVs and indels).
- SBS, DBS and ID mutational signature activity as binary indicators. Signatures were analysed if they were identified in <50% of tumours in the respective cohort.
- Immune escape status.

For analyses that required CNA profiles, smaller MSS and MSI cohorts comprising 810 (141 deaths) and 222 (40 deaths) individuals were used. The following variables were analysed using these smaller cohorts:

- Driver gene mutation status. Driver genes were considered mutated in a tumour if: (1) they contained an oncogenic mutation as defined by OncoKB and dNdScv annotation, (2) were homozygously deleted, or (3) were affected by a large copy number gain (total copy number state >5 for non-WGD tumours and total copy number state >10 copies for WGD tumours).
- WGD status.
- Chromosome-arm-level gains and deletions.
- Total SV number.

For each cohort, variables were only tested if at least 5% of deaths were present in each category. A variable was considered correlated with survival if it improved model fit using ANOVA and the *z*-test provided association evidence. The Benjamini–Hochberg procedure was used to determine FDR to adjust for multiple testing. Proportional hazards assumption violations were analysed for each test. In multiple Cox regression analysis, *P* = 0.05 was considered significant.

#### Normal colorectal epithelial cell signatures

Numbers and proportions of SNVs associated with each SBS signature were obtained from a previous study^44^. For cases in which multiple crypts from the same colon region were sampled in a single individual, the median number and proportion of SNVs associated with each SBS signature was computed across these samples. For cases in which multiple crypts from the same colon region had been sampled in a single participant, the median number and proportion of variants attributed to each signature was considered. Supplementary Fig. 11 shows data from ref. ^44^. IDA closely resembles ID18. *P* values were computed using Wilcoxon tests.

### Software used

Supplementary Table 1 lists software versions used in this study and their URLs.

### Reporting summary

Further information on research design is available in the Nature Portfolio Reporting Summary linked to this article.

## Online content

Any methods, additional references, Nature Portfolio reporting summaries, source data, extended data, supplementary information, acknowledgements, peer review information; details of author contributions and competing interests; and statements of data and code availability are available at 10.1038/s41586-024-07747-9.

## Supplementary information

Supplementary Information Guide This file contains a full guide for the Supplementary Results, Note, Figures and Tables.

Reporting Summary

Supplementary Information This file contains Supplementary Results 1–9, Supplementary Note, Supplementary Figs 1–11 and Supplementary References.

Supplementary Tables Supplementary Tables 1–38 – see the Supplementary Information guide for full descriptions.

## Data Availability

Genomics England permits access to data used for this study subject to the following conditions. Research on the de-identified patient data used in this publication can be carried out in the Genomics England Research Environment subject to a collaborative agreement that adheres to patient-led governance. All interested readers will be able to access the data in the same manner that the authors accessed the data. For more information about accessing the data, interested readers may contact research-network@genomicsengland.co.uk or access the relevant information on the Genomics England website (https://www.genomicsengland.co.uk/research). To expedite follow-on analyses, we have made available in the Genomics England Research Environment a Genomic Data Table that provides for each patient and their tumour, all the individual clinical and molecular variable data used in this article (Supplementary Information Guide).
