# Supplementary material for: The genomic landscape of 2,023 colorectal cancers
Source: Nature. 2024 Aug 7;633(8028):127–36. doi: 10.1038/s41586-024-07747-9 (PMC11374690; doi:10.1038/s41586-024-07747-9)
Supplement: Supplementary file 1 — This file contains a full guide for the Supplementary Results, Note, Figures and Tables. [file 41586_2024_7747_MOESM1_ESM.pdf]

nature portfolio

https://doi.org/10.1038/s41586-024-07747-9

Supplementary information

The genomic landscape of 2,023 colorectal cancers

In the format provided by the
authors and unedited

SUPPLEMENTARY INFORMATION GUIDE

SUPPLEMENTARY RESULTS

The data provided here represent an expansion of the findings presented in selected sections of the main manuscript, Figures or Extended Data items.

Page	Title
1	*Supplementary Result 1. Co-occurrence of mutational signatures and other molecular changes*
2	*Supplementary Result 2. Selected driver gene mutations identified in primary CRCs*
8	*Supplementary Result 3. Ras-Raf-Mek-Erk pathway driver mutations*
8	*Supplementary Result 4. SVs underlie some chromosomal-scale CNAs*
9	*Supplementary Result 5. Clinical actionability*
9	*Supplementary Result 6. Stage, survival and molecular variables*
10	*Supplementary Result 7. Five exemplar analyses of rare molecular sub-types of CRC*
13	*Supplementary Result 8. Four exemplar analyses of the non-coding and/or non-nuclear CRC genome*
14	*Supplementary Result 9. Micro-organisms, the non-human CRC-associated genome*
15	*Supplementary Note: Extended synopsis of study*

SUPPLEMENTARY FIGURES

Page Title

18 *Supplementary Figure 1. Removing bias introduced by soft clipping of semi-aligned reads.* An alignment of six reads to a reference sequence containing an A/T variant is shown. Bold black T and red A represent reference and alternate alleles respectively. Soft clipping is represented by strikethrough. Without soft clipping, three reads would support both the reference (T) and alternative (A) alleles, resulting in an unbiased variant allele frequency (VAF) of 3/6=0.5. (a) Read R3 is soft clipped until five consecutive matches with the reference are obtained. After clipping, only two reads support the alternate allele (A), whilst three reads support the reference allele (T), resulting in a biased VAF of 2/5=0.4. (b) FixVAF clips all reads by five bases, regardless of whether they contain a variant site or support a reference or alternate allele. Reads supporting both the reference and alternate alleles are now clipped by five bases. In this example, FixVAF would compute a VAF of 2/4=0.5, and therefore remove bias.

19 *Supplementary Figure 2. Overview of copy number aberration calling pipeline.* BAM: binary sequence alignment map, CCF: cancer cell fraction, SNV: single nucleotide variant, vcf: variant call format.

20 *Supplementary Figure 3. Overview of structural-variant-calling pipeline.* BAM: binary sequence alignment map, PCAWG: The Pan-Cancer Analysis of Whole Genomes, SV: structural variant.

21 *Supplementary Figure 4. Ancestry.* Upper, principal component analysis of 2,017 CRC patients showing major European, smaller clusters of Asian and African ancestry, and individuals of mixed ancestry outside the major cluster. Lower, high correspondence between 16 self-reported ancestry groups and PCA classification.

22 *Supplementary Figure 5. Association between SNV VAF and numbers of driver mutations called in primary microsatellite stable (MSS) colorectal cancer (CRC) samples (n=1956), stratified by median single nucleotide variant (SNV) variant allele frequency (VAF).* Potential driver mutations were defined as any coding variant annotated as probably pathogenic from 63 driver genes previously identified in MSS CRC[2,3,4,7 3,30-32]. To the left of the dashed line, it is likely that low median VAF results from low cancer cell purity and this causes a failure to identify some driver mutations. Tumour samples below the threshold were therefore excluded from the analysis.

23 *Supplementary Figure 6. IntOGen pipeline.* Red circles indicate non-coding elements with *Q*<0.01. The overdispersion of the test statistic for driver status revealed by the QQ plots suggests numerous non-coding drivers. However, we remain cautious about declaring driver status for any specific gene or element based on genomic data alone. For example, analysis of the *CSMD3* distal promoter in MSS primary samples revealed two recurrently mutated positions at chr8:113437866 (n=7, 0.5%) and chr8:113437869 (n=11, 0.7%). The chr8:113437866 hotspot exclusively comprised by GTT>GGT transversions. The chr8:113437869 hotspot was characterised by CTT>CGT (n=5), CTT>CCT (n=4) and CTT>CAT (n=2) substitutions. Each was respectively located 3bp and 1bp immediately upstream of a CTCF binding motif predicted by the JASPAR database [33] and supported by CTCF ChIP data in colon [34] . This is consistent with the significant functional impact signals found at the CTCF binding site overlapping the hotspots (chr8:113437400-113438400). In line with previous observations, five of 12 CTT>CGY and three

of four CTT>CCT mutations were attributable to SBS17b and SBS17a, respectively [35,36] . CTCF binding sites are known to be enriched in mutations, creating mutation clusters and hotspots [37]. Based on this, we cannot exclude the possibility that these two hotspots in the *CSMD3* distal promoter are passenger mutations.

24 *Supplementary Figure 7. Quantile-quantile plots of OncodriveFML results for non-coding elements in MSS primary tumours.*

25 *Supplementary Figure 8. Structural variant (SV) signature extraction.* (a) Multimodal SV size and replication timing distributions. Dashed lines represent thresholds used to categorize variants for signature extraction. (b) To assess stability of SV signature extraction using the hierarchical Dirichlet process (HDP), the cohort was randomly split into halves and signatures extracted independently from each. Nine signatures extracted from the cohort halves showed high similarity between halves (red and blue grey; cosine similarity >0.9) and high similarity with signatures extracted from the full cohort and were therefore included in subsequent analyses. MSS: microsatellite stable; MSI: microsatellite unstable.

26 *Supplementary Figure 9. CN signatures.* (a) Deconvolution of the four de novo signatures using the 21 COSMIC CNV signatures, to identify six contributing COSMIC signatures. CNV48A is a heterogeneous signature, dominated by heterozygous segments of 3-8 copies. It is decomposed in to three COSMIC signatures: CN17, associated with homologous recombination deficiency (HRD) and tandem duplications (42.18%); CN6, associated with chromothripsis (29/72%); and CN20, which has a currently unexplained aetiology (28.1%). CNV48B is comprised primarily of heterozygous segments of 3-4 copies with a length of >40Mb it is deconvoluted into a single cosmic signature CN2, associated with tetraploidy. CNV48C is dominated by heterozygous segments with a copy number (CN) of 2 and is decomposed to CN1, indicative of a diploid state. CNV48D is dominated by LOH segments with a CN of 1 and heterozygous segments with a CN of 2 and to a lesser extent 3-4, it deconvoluted into CN9 which has previously been associated with chromosomally unstable diploid tumours. For each de novo CN signature on the top left of each plot the contributing COSMIC signatures are provided on the right, alongside the final refitted signature on the bottom left. (b) Selection plot showing the mean sample cosine difference and average stability for de novo extraction of 1-30 copy number signature. The accepted solution contained four de novo signatures.

31 *Supplementary Figure 10. Comparison of methods for neoantigen prediction.* Lower and upper triangle and diagonal cells represent pairwise scatter plots, correlation statistics and density plots respectively (green: MSS cancers; red: MSI; yellow: POL). NA mutation: number of unique mutations giving rise to one or more neoantigens; NA peptide: number of unique neoantigen peptides; NA total: number of antigenic HLA-peptide interactions detected; Expr NA total: number of antigenic HLA-peptide interactions detected in genes expressed in ≥10% TCGA CRCs. Measures presented on $\log_{10}$ scale.

32 *Supplementary Figure 11. Signature activity in normal crypt epithelial cells from the right, transverse and left colon.* Where multiple crypts from the same colon region had been sampled in a single participant, the median number and proportion of variants attributed to each signature were considered. Data from Lee-Six *et al* [38]. IDA closely resembles ID18. *P*-values were computed using two-sided Wilcoxon rank sum tests. *n*: number of participants, Trans: transverse colon.

SUPPLEMENTARY TABLES

Supplementary Table 1. Software and data used in this study.

Supplementary Table 2. Quality control and exclusion of tumour samples from The 100,000 Genomes Project colorectal cancer domain. A sample is removed during quality control if it fails at least one quality control filter. VAF: variant allele frequency.

Supplementary Table 3. Small-scale (SBS, DBS and ID) mutational signatures: presence, activity and co-occurrence. (a) The frequencies and activities of each signature (presence or absence) in the full set of CRCs. The frequencies in COSMIC v3 are shown for comparison. These are based on 841 CRC exomes (for SBS), 17 genomes (for dBS) and 127 genomes (for ID). The signature activity (proportion of all SBS, DBS or ID mutations in any cancer) is summarised as the mean and variance across all cancers. Frequencies and mean activities for patients without prior genotoxic therapy are also shown for comparison. (b) *Pearson's correlation coefficients for pairs of mutational signatures* (.cor), significance of the correlations (.pval), and Bonferroni adjusted p-values (.padj).

Supplementary Table 4. Identified coding driver genes from analysis of MSS, MSI, POL and Metastasis subtypes. The results of previous other driver gene detection studies or database entires are shown. The driver gene identification programs within IntOGen that resulted in assignment of driver status are also shown. CGC: Cancer Gene Census; ONC: oncogenic/pathogenic variant; VUS: variant of unknown significance. N. Carriers VUS indicates tumours containing 0 ONC and 1+ VUS mutation in the given gene. Note that many of these putative drivers have several functions and/or their roles are incompletely determined. The functional role should be interpreted cautiously.

Supplementary Table 5. Frequency of driver gene mutations and enrichment across CRC subtypes. Carriers are those with one or more predicted pathogenic mutations in the candidate driver gene. Fisher's exact test was used to test for signifiant differences in pairwise carrier frequencies between CRC subtypes. Enriched subtypes are those in which the Fisher's exact test P-value for that subtype compared to all other CRC tumours was < 0.0125 (correcting for testing for 4 subtypes) and odds ratio (OR) >1. Frequencies are derived from OncoKB annotation and represent minimum numbers of pathogenic variants. Actual observed somatic variant frequencies are shown in Supplementary Table 4.

Supplementary Table 6. Frequency of recurrent driver mutations across CRC subtypes. All mutations in candidate driver genes with carrier count >40 are listed. Fisher's exact test was used to test for significant differences in pairwise carrier frequencies between CRC subtypes. Enriched subtypes are those in which the Fisher's exact test P-value for that subtype compared to all other CRC tumours was <0.0125 (correcting for testing for 4 subtypes) and odds ratio (OR) >1. Bold annotation shows coding oligonucleotide tracts.

Supplementary Table 7. Indel hotspots at oligonucleotide repeats selected for the detailed analysis of evidence to support driver status. *two different loss-of-function mutations or high variant allele frequency suggestive of homozygosity.

Supplementary Table 8. Distribution of per tumour driver mutation counts by CRC subtype. Pathogenic mutations from 185 candidate driver genes were included in the per tumour counts.

Supplementary Table 9. Driver genes reported in other studies of CRC and validation status in the IntOGen analyses of the four main CRC sub-types in this study.

Supplementary Table 10. Hotspots of simple structural variants. Related data are shown in **Fig. 1b**. The top table shows SV signature frequency (presence/absence) by tumour group (POL tumours too rare to show). The lower tables show SV hotspots. bp: base pair; COSMIC: Catalog of Somatic Mutations in Cancer; TCGA: The Cancer Gene Atlas; PCAWG: The Pan-Cancer Analysis of Whole Genomes; TSG: Tumour Suppressor Gene. *Number of samples with at least one SV break end in hotspot. **Minimum FDR from all enriched SV types. ***Higher mean replication timing values correspond to earlier replication. ****Whether the region was identified as recurrently deleted or amplified by TCGA [7]. *****Whether an overlapping hotspot was reported by PCAWG [103]. Candidate genes are either SNV/indel drivers (**Supplementary Table 4**) or the only coding gene within the hotspot region. Note that the SNV/indel driver may not be the site of the most frequent breakpoints in the region. In MSS cancers, 52% (45/87) of SV hotspots were at fragile sites, compared with 87% (20/23) in MSI cancers (Fisher's exact, P=0.002).

Supplementary Table 11. Kinase gene fusions identified. The following genes were considered: *ALK, BRAF, EGFR, ERBB2, ERBB4, FGFR1, FGFR2, FGFR3, KIT, MET, NTRK1, NTRK2, NTRK3, RET,* and *ROS1*.

Supplementary Table 12. Enrichment of complex SVs in primary MSS cancers with arm-level CNAs. Complex SV considered present if a chromothripsis or unclassified complex SV was identified on the same chromosome.

Supplementary Table 13. Extrachromosomal DNA (ecDNA) detected across CRC subtypes and its contribution to common oncogene amplification. (a) Counts of tumours carrying at least one ecDNA amplicon across tumour subtypes. For example, a tumour was counted as "Circular" if ≥ 1 circularised amplicon detected, otherwise "BFB" if ≥ 1 BFB amplicon detected until "No amp" where no valid amplicon detected. *(b) ecDNA classification of commonly amplified oncogenes in CRC subtypes.* Classification was restricted to gene amplifications with a total copy number ≥ 5 in diploid tumours and ≥ 10 in tetraploid tumours (i.e. "big gains"). Primary POL cohort not shown as no genes were amplified. *(c) Association between tumours with ≥ 1 circularised amplicon detected and presence of chromothripsis.* Significance was assessed using Fisher's exact test.

Supplementary Table 14. Associations of copy number signatures with MSS and MSI status.

Supplementary Table 15. Arm-level copy number gains and deletions. Significantly enriched copy number gain (Gain) . S[illegible]ificantly enriched copy number loss (Del) . H[illegible]ology in PAR between chromosomes X and Y means results for sex chromosomes should be interpreted with caution.

Supplementary Table 16. Focal copy number gains (AMP) and deletions (DEL). The table shows the locations and frequencies of each shared CNA region in primary MSS, primary MSI, primary POL and metastasis MSS cancers. Annotation is as follows: Ambiguous - unclear whether oncogene or TSG; CRC peak TCGA GISTIC - overlap in 1+ gene from this GISTIC analysis with TCGA CRC GISTIC analysis [89]; Pan-cancer peak TCGA GISTIC - overlap in 1+ gene from this GISTIC analysis with TCGA CRC GISTIC analysis [89]; CRC highlighted: TCGA GISTIC: Prioritised candidate in

colorectal peak GISTIC analysis in TCGA [89]; and Pan-cancer highlighted TCGA GISTIC - Prioritised candidate in pan-cancer peak GISTIC analysis in TCGA [89]. SV hotspot overlap (*) only shown for primary MSS and primary MSI. Candidate genes are either identified SNV/indel drivers in the region (Supplementary Table 4) or the only coding gene within that focal region. Fragile sites include *FHIT, MACROD2, WWOX, PDE4D, CCSER1, PRKN* and *RBFOX1*. Frequency includes all CNAs of that type that involve the focal peak region.

Supplementary Table 17. Comparison of focal copy number gains and deletions identified in 100KGP, PCAWG and TCGA. Bold: identified in 1KGP and either PCAWG or TCGA. *Identified in PCAWG and TCGA.

Supplementary Table 18. Second hits at driver genes in MSS and MSI cancers. Genes with overall second hit frequencies >20% are shown. Note that possible homozygous mutations not resulting from LOH events are not shown owing to uncertainty in identifying them based on read depth and purity estimates. This leads in particular to under-estimation of second hits at hotspots, such as indels at short repeats in MSI cancers.

Supplementary Table 19. ReactomeDB pathway annotation of the 196 driver genes in the analysis of the four main CRC subtypes.

Supplementary Table 20. Genetic features targeted by an approved therapy.

Supplementary Table 21. Genetic features for which compelling biological evidence supports prediction of response to a therapy.

Supplementary Table 22. Coding driver mutations, with data on efficacy and selectivity from shinyDepMap and on druggability from canSAR. Note that no data are available for some identified drivers and these are not shown. Similarly, druggability is not shown where information does not exist.

Supplementary Table 23. MSS cluster features. For most variables, per cluster average is shown if any cluster group showed a significant difference (FDR < 0.05) compared to the set of MSS tumours. All driver genes detected (**Supplementary Table 4**) are shown even if no association with cluster is present. A few selected additional variables are shown. Variables either represent mean proportion of cancers with that feature or mean value for continuous variables. All tumours are primary and treatment-naïve. Average values for all MSS tumours, including those that failed clustering (see **Methods**), are shown for comparison. The cancers that failed clustering show no special distinguishing or outlier features in that variable values are almost always intermediate between the cluster groups with the lowest and highest values. Additional variables (not shown here) that showed no significant difference (FDR ≥ 0.05) between groups were: BMI; (self-reported) ethnicity; tumour grade; all other mutational signatures; specific *KRAS* variants (G12A, G12C, G12D, G12R, G12S, G12V, G13C, G13D, Q61H, Q61K, Q61L, K117N, A146T or A146V); microbiome diversity; prevalence of top 20 bacterial genera; and whether immune escape was caused by mutations or loss of heterozygosity in HLA or other antigen presenting genes (*B2M, CALR, CANX, CIITA, ERAP1, ERAP2, HSPBP1, IRF1, PDIA3, PSMA7, PSME1, PSME2, TAP1* and *TAP2*). Fisher's exact test was used for binary and categorical variables, and the Wilcoxon rank-sum test for continuous variables. False discovery rate (FDR) was calculated with the Benjamini-Hochberg method: ns (FDR ≥ 0.05), * (0.01 ≤ FDR < 0.05), ** (0.001 ≤ FDR < 0.01), *** (FDR < 0.001).

Supplementary Table 24. Clonal and subclonal driver mutations across CRC subtypes.

Supplementary Table 25. Non-coding elements identified by OncodriveFML as significantly enriched for mutations of high functional impact. Primary MSS cancers only were analysed. Recurrently mutated non-coding elements with Q<0.01 are shown.

Supplementary Table 26. Testing for co-occurrence relationships between protein-coding and non-canonical splice region mutations in APC and SMAD4. Fisher's exact test was used to test for significant differences in pairwise carrier frequencies between oncogenic protein-coding mutations, loss of heterozygosity (LOH), bi-allelic loss and non-canonical splice mutations in *APC* and *SMAD4.* Fisher's exact P-value < 0.01 was taken to indicate statistical significance (correction for testing 5 subtypes), with odds ratio (OR) > 1 indicating co-occurrence and OR < 1 indicating mutual exclusivity. APC chr5:112815487 A>G is c.835-8A>G. *SMAD4* chr18:51058332 A>G is c.788-8A>G. These are the most common recurrent mutations with predicted neo-splice site activity. The 11/10/01/00 terminology refers to presence of both mutation types 1 and 2, presence of type 1 only, presence of type 2 only, and presence of neither type. In summary, the neo-splice site mutations typically occur with one other truncating mutation or with LOH, indicating pathogenicity.

Supplementary Table 27. Mitochondrial mutations. P-values and Q-values < 0.05 highlighted blue. CI: confidence interval.

Supplementary Table 28. Numbers of microbial taxa identified. (a) Raw microbial read numbers for primary, metastases, local recurrences and blood samples. (b) Number of taxa after decontamination. (c) Numbers of taxa significantly associating with metadata categories according to the multivariate MaAsLin2 package [139]. The analysis was run at species and genus level. "CRC", "Control", and "Oral" columns indicate the number of those species or genera were previously defined as CRC-associated, control-associated, or oral species. "Available for comparison" indicates how many taxa in each category were prevalent enough to be considered.

Supplementary Table 29. Microbiome genus-level clinicopathological correlations.

Supplementary Table 30. Microbiome species-level clinicopathological correlations.

Supplementary Table 31. Clinical data availability.

Supplementary Table 32. Correlation of clinical variables with mutational burden and signature activities. SBS, DBS, ID and SV signature activities were quantified as the number of variants attributed to each signature in each sample. CN signatures were considered binary variables indicating the presence or absense of the signature. For tumour mutational burden, SBS, DBS, ID and SV signatures, estimates were made using multiple linear regression. For CN signatures and WGD, estimates were made using multiple logistic regression. Predictor and response variable values were subsequently scaled to means of 0 and standard deviations of 1. Analysis was restricted to MSS primary and MSI cancers due to small numbers of POL and MSS metastasis samples with full anatomical location and stage data. P-values < 0.05 highlighted blue. CI: confidence interval; Bonf.: Bonferroni-corrected. As appropriate, regression models included co-variables age, primary tumour site, and stage, together with *TP53* status where this had been shown previously to associate with the outcome variable.

Supplementary Table 33. Correlation of clinical variables with driver gene mutations. Predictor variables were scaled to means of 0 and standard deviations of 1. Logistic regression estimates therefore represent standardised coefficients. Analysis was restricted to primary MSS and primary MSI cancers due to low sample numbers elsewhere. P-values < 0.05 highlighted blue. CI: confidence interval; Bonf.: Bonferroni-corrected.

Supplementary Table 34. Correlation of clinical variables with recurrent copy number alterations. Predictor variables were scaled to means of 0 and standard deviations of 1. Logistic regression estimates therefore represent standardised coefficients. Analyses were restricted to primary MSS and primary MSI tumours due to sample numbers. P-values < 0.05 highlighted blue. CI: confidence interval; Bonf.: Bonferroni-corrected. Proportion of genome with CNA was calculated as proportion of genome with total copy number not equal 4 If tumour has undergone whole genome duplication, and 2 otherwise.

Supplementary Table 35. Full details of driver genes discovered in the four main sub-types and in the analysis of Primary MSS cancers by anatomical location. Note that this table is an extension of **Supplementary Table 4** to include drivers detected in specific locations of the large bowel. Forty-eight drivers in the table were discovered in analysis of MSS primary cancers by anatomical location. Note that location-specific drivers *PTPRK*, *THEMIS* and *UTRN* are also at sites of SV hotspots, and that in addition to the 241 genes in this table, the following eight genes were separately identified as putative drivers from SV analysis: *CDKAL1, BRD4, EZH2, IGF2, KCNQ1, MYC, UBE3A and VMP1.* Driver total from the whole study thus reached 249 based on the addition of location-specific drivers to drivers in the four CRC sub-types and putative SV and CNA drivers.

Supplementary Table 36. Candidate driver genes identified by separate of analysis of each of the four genomic cluster sub-groups of MSS primary tumours. Thirty-five candidate drivers in the table (bold) were discovered solely in this analysis, and not in the analysis by MSS primary/MSI/POL/MSS metastasis sub-types, or in the analysis of MSS primary cancers by anatomical location. Since the cluster sub-groups are subject to refinement in future analyses, we regard these as "candidate" driver genes to reflect the additional uncertainty regarding their statuses.

Supplementary Table 37. Variation of selected molecular variables (mutation burdens, mutation signatures, driver mutations and cluster groups) with (a) anatomical location and (b) age at sampling. See also representation of data in **Fig. 4**.

Supplementary Table 38. Terminology used to describe different copy number states detected. Copy number of each allele is shown for situations in which whole-genome doubling has or has not occurred. t_{CN} = total copy number.

SUPPLEMENTARY ITEM IN 100,000 GENOMES RESEARCH ENVIRONMENT

Genomic Data Table

Contains individual tumour data for each of the following variables:

Tumour sample platekey sequencing identifier
Participant ID
Sex
Ethnicity (self-reported)
Origin from primary tumour (P) or metastasis (M)
Grade
Date of diagnosis
Date of sampling
Time from diagnosis to sampling (days)
Age at diagnosis (years)
Date of last follow-up
Date of death
Dead (1) or Alive (0)
Time from diagnosis to death or last follow-up (days)
Age of death (years)
Molecular sub-type (MSS, MSI, POL)
MSS cluster sub-group
WGD (Yes/No)
Median sequencing coverage autosomes, paired normal
Median sequencing coverage autosomes, tumour
Total no. SNVs
Total no. indels
Total no. SVs
Total no. CNAs
Estimated purity (0-1)
Estimated ploidy
SBSs, DBSs and IDs assigned to signatures
SVs assigned to signatures
CNAs assigned to signatures
Specific driver gene (n=249) mutations including 2nd hits
Homologous recombination repair deficiency (Yes/No)
Chromosome arm-level CNAs
Focal CNAs
SV hotspot changes
Selected fusion gene changes and ecDNA group
Immune escape including driver mutations
Microbiome metrics

Available to all individuals registered with 100,000 Genomes Research Environment. The 100,000 Genomes Project data can be accessed by those at registered institutions who complete the online form at: https://www.genomicsengland.co.uk/research/academic/join-gecip.
