# Supplementary material for: The genomic landscape of 2,023 colorectal cancers
Source: Nature. 2024 Aug 7;633(8028):127–36. doi: 10.1038/s41586-024-07747-9 (PMC11374690; doi:10.1038/s41586-024-07747-9)
Supplement: Supplementary file 2 — Reporting Summary [file 41586_2024_7747_MOESM2_ESM.pdf]

nature portfolio

Corresponding author(s): Ian Tomlinson

Last updated by author(s): Jun 3, 2024

Reporting Summary

Nature Portfolio wishes to improve the reproducibility of the work that we publish. This form provides structure for consistency and transparency in reporting. For further information on Nature Portfolio policies, see our Editorial Policies and the Editorial Policy Checklist.

Statistics

For all statistical analyses, confirm that the following items are present in the figure legend, table legend, main text, or Methods section.

n/a	Confirmed	
☐	☒	The exact sample size (*n*) for each experimental group/condition, given as a discrete number and unit of measurement
☒	☐	A statement on whether measurements were taken from distinct samples or whether the same sample was measured repeatedly
☐	☒	The statistical test(s) used AND whether they are one- or two-sided<br>*Only common tests should be described solely by name; describe more complex techniques in the Methods section.*
☐	☒	A description of all covariates tested
☐	☒	A description of any assumptions or corrections, such as tests of normality and adjustment for multiple comparisons
☐	☒	A full description of the statistical parameters including central tendency (e.g. means) or other basic estimates (e.g. regression coefficient) AND variation (e.g. standard deviation) or associated estimates of uncertainty (e.g. confidence intervals)
☐	☒	For null hypothesis testing, the test statistic (e.g. *F*, *t*, *r*) with confidence intervals, effect sizes, degrees of freedom and *P* value noted<br>*Give P values as exact values whenever suitable.*
☒	☐	For Bayesian analysis, information on the choice of priors and Markov chain Monte Carlo settings
☒	☐	For hierarchical and complex designs, identification of the appropriate level for tests and full reporting of outcomes
☐	☒	Estimates of effect sizes (e.g. Cohen's *d*, Pearson's *r*), indicating how they were calculated

Our web collection on statistics for biologists contains articles on many of the points above.

Software and code

Policy information about availability of computer code

Data collection

The standard Illumina sequencing pipeline (NorthStar v2.6.53.23) implemented in the 100,000 Genomes Project was used. Poor quality sequenced samples were identified based on % mapped reads, % chimaeric DNA fragments, averae insert size, AT/CG dropout, and evenness of local coverage.

Other data accessed comprises
CADD 1.6 https://cadd.gs.washington.edu
CancerMine February 2021 http://bionlp.bcgsc.ca/cancermine/
COSMIC Cancer Gene Census 92 https://cancer.sanger.ac.uk/census
COSMIC Reference Mutational Signatures 3.2 https://cancer.sanger.ac.uk/signatures/
eHOMD - http://www.homd.org/
ENCODE - https://www.encodeproject.org
Ensembl 101 https://www.ensembl.org/index.html
GATK pathseq resource bundle - ftp://ftp.broadinstitute.org/bundle/beta/PathSeq/
GnomAD 2. l https ://gnomad.broadi nstitute.org/downloads#v2-constraint
Homo sapiens GRCh38Decoy reference assembly - http://emea.support.illumina.com/sequencing/sequencing_software/igenome.html
lntOGen Gene Annotations 1 February 2020 https://www.intogen.org/download?file=lnt0Gen-Cohorts-2020020l.zip
OncoKB 3.3 https://www.oncokb.org/
Protein Data Bank March 2020 https://www.rcsb.org/#Category-download ReplicationDomain - https://www2.replicationdomain.com
Segmental Duplication Database - https://humanparalogy.gs.washington.edu

UCSC Genome Browser - https://hgdownload.soe.ucsc.edu/downloads.html

Comparisons with previous larger-scale cancer sequencing utilised data from the following sources that contain accessible data or instructions for access to that data.

Bailey, M. H., C. Tokheim, E. Porta-Pardo, S. Sengupta, D. Bertrand, A. Weerasinghe, A. Colaprico, M. C. Wendl, J. Kim, B. Reardon, P. K. Ng, K. J. Jeong, S. Cao, Z. Wang, J. Gao, Q. Gao, F. Wang, E. M. Liu, L. Mularoni, C. Rubio-Perez, N. Nagarajan, I. Cortés-Ciriano, D. C. Zhou, W. W. Liang, J. M. Hess, V. D. Yellapantula, D. Tamborero, A. Gonzalez-Perez, C. Suphavilai, J. Y. Ko, E. Khurana, P. J. Park, E. M. Van Allen, H. Liang, M. S. Lawrence, A. Godzik, N. Lopez-Bigas, J. Stuart, D. Wheeler, G. Getz, K. Chen, A. J. Lazar, G. B. Mills, R. Karchin and L. Ding (2018). Comprehensive Characterization of Cancer Driver Genes and Mutations. Cell 173(2): 371-385.e318.

Giannakis, M., X. J. Mu, S. A. Shukla, Z. R. Qian, O. Cohen, R. Nishihara, S. Bahl, Y. Cao, A. Amin-Mansour, M. Yamauchi, Y. Sukawa, C. Stewart, M. Rosenberg, K. Mima, K. Inamura, K. Nosho, J. A. Nowak, M. S. Lawrence, E. L. Giovannucci, A. T. Chan, K. Ng, J. A. Meyerhardt, E. M. Van Allen, G. Getz, S. B. Gabriel, E. S. Lander, C. J. Wu, C. S. Fuchs, S. Ogino and L. A. Garraway (2016). Genomic Correlates of Immune-Cell Infiltrates in Colorectal Carcinoma. Cell Rep 15(4): 857-865.

Grasso, C. S., M. Giannakis, D. K. Wells, T. Hamada, X. J. Mu, M. Quist, J. A. Nowak, R. Nishihara, Z. R. Qian, K. Inamura, T. Morikawa, K. Nosho, G. Abril-Rodriguez, C. Connolly, H. Escuin-Ordinas, M. S. Geybels, W. M. Grady, L. Hsu, S. Hu-Lieskovan, J. R. Huyghe, Y. J. Kim, P. Krystofinski, M. D. M. Leiserson, D. J. Montoya, B. B. Nadel, M. Pellegrini, C. C. Pritchard, C. Puig-Saus, E. H. Quist, B. J. Raphael, S. J. Salipante, D. S. Shin, E. Shinbrot, B. Shirts, S. Shukla, J. L. Stanford, W. Sun, J. Tsoi, A. Upfill-Brown, D. A. Wheeler, C. J. Wu, M. Yu, S. H. Zaidi, J. M. Zaretsky, S. B. Gabriel, E. S. Lander, L. A. Garraway, T. J. Hudson, C. S. Fuchs, A. Ribas, S. Ogino and U. Peters (2018). Genetic Mechanisms of Immune Evasion in Colorectal Cancer. Cancer Discov 8(6): 730-749.

Liu, Y., N. S. Sethi, T. Hinoue, B. G. Schneider, A. D. Cherniack, F. Sanchez-Vega, J. A. Seoane, F. Farshidfar, R. Bowlby, M. Islam, J. Kim, W. Chatila, R. Akbani, R. S. Kanchi, C. S. Rabkin, J. E. Willis, K. K. Wang, S. J. McCall, L. Mishra, A. I. Ojesina, S. Bullman, C. S. Pedamallu, A. J. Lazar, R. Sakai, V. Thorsson, A. J. Bass and P. W. Laird (2018). Comparative Molecular Analysis of Gastrointestinal Adenocarcinomas. Cancer Cell 33 (4): 721-735.e728.

Martincorena, I., K. M. Raine, M. Gerstung, K. J. Dawson, K. Haase, P. Van Loo, H. Davies, M. R. Stratton and P. J. Campbell (2017). Universal Patterns of Selection in Cancer and Somatic Tissues. Cell 171(5): 1029-1041.e1021.

TCGA Network (2012). Comprehensive molecular characterization of human colon and rectal cancer. Nature 487(7407): 330-337.

Seshagiri, S., E. W. Stawiski, S. Durinck, Z. Modrusan, E. E. Storm, C. B. Conboy, S. Chaudhuri, Y. Guan, V. Janakiraman, B. S. Jaiswal, J. Guillory, C. Ha, G. J. Dijkgraaf, J. Stinson, F. Gnad, M. A. Huntley, J. D. Degenhardt, P. M. Haverty, R. Bourgon, W. Wang, H. Koeppen, R. Gentleman, T. K. Starr, Z. Zhang, D. A. Largaespada, T. D. Wu and F. J. de Sauvage (2012). Recurrent R-spondin fusions in colon cancer. Nature 488(7413): 660-664.

Yaeger, R., W. K. Chatila, M. D. Lipsyc, J. F. Hechtman, A. Cercek, F. Sanchez-Vega, G. Jayakumaran, S. Middha, A. Zehir, M. T. A. Donoghue, D. You, A. Viale, N. Kemeny, N. H. Segal, Z. K. Stadler, A. M. Varghese, R. Kundra, J. Gao, A. Syed, D. M. Hyman, E. Vakiani, N. Rosen, B. S. Taylor, M. Ladanyi, M. F. Berger, D. B. Solit, J. Shia, L. Saltz and N. Schultz (2018). Clinical Sequencing Defines the Genomic Landscape of Metastatic Colorectal Cancer. Cancer Cell 33(1): 125-136.e123.

Data analysis

Software Version (where applicable) URL
ActivePathways 1.1.0 https://cran.r-project.org/web/packages/ActivePathways/index.html
alleleCount-FixVAF - https://github.com/danchubb/alleleCount-FixVAF
AmpliconArchitect 1.2 https://github.com/virajbdeshpande/AmpliconArchitect
AmpliconClassifier 0.4.6 https://github.com/jluebeck/AmpliconClassifier
ANNOVAR 2018v16 https://annovar.openbioinformatics.org/en/latest/user-guide/download/
ape 5.5 https://cran.r-project.org/web/packages/ape/index.html
Battenberg 2.2.8 https://github.com/Wedge-Oxford/battenberg
bcftools 1.9 http://www.htslib.org/download/
bedops 2.4.39 https://github.com/bedops/bedops
bedtools 2.3.0 https://github.com/arq5x/bedtools2
bwa 0.7.17 https://github.com/lh3/bwa
cBase 1.0 http://genetics.bwh.harvard.edu/wiki/sunyaevlab/cbase
Ccube 1.0 https://github.com/keyuan/ccube
CleanCNA 0.1.0 https://github.com/afrangou/CleanCNA
ClusterSV February 2019 https://github.com/cancerit/ClusterSV
CNAqc 1.0.0 https://github.com/caravagnalab/CNAqc
COSMIC June 2022 https://cancer.sanger.ac.uk/signatures/
Delly 0.7.8 https://github.com/dellytools/delly/releases/download/v0.7.9/delly_v0.7.9_linux_x86_64bit
DISCOVER 0.9 https://github.com/NKI-CCB/DISCOVER
dNdSCV 0.1.0 https://github.com/im3sanger/dndscv
DPClust 2.2.8 https://github.com/Wedge-Oxford/dpclust
fastMitoCalc 1 https://lgsun.irp.nia.nih.gov/hsgu/software/mitoAnalyzer/index.html
GISTIC 2.0.2.3 https://github.com/broadinstitute/gistic2
GTAK Pathseq 4.0.4.0 https://github.com/broadinstitute/gatk/releases
hdp 0.1.5 https://github.com/nicolaroberts/hdp
HotMaps3D 1.1.3 https://github.com/KarchinLab/HotMAPS
HRDetect (from signature.tools.lib) 0.0.0.9000 https://github.com/Nik-Zainal-Group/signature.tools.lib
igraph 1.2.4.2 https://igraph.org/r/
IntOGen February 2021 https://bitbucket.org/intogen/intogen-plus/src/master
Isaac 03.16.02.19 https://github.com/Illumina/Isaac3/releases/tag/iSAAC-03.16.02.19
LEfSe Galaxy version 1.0 https://huttenhower.sph.harvard.edu/galaxy/
LOHHLA 1.0 https://bitbucket.org/mcgranahanlab/lohhla/src/master/
Lumpy 0.2.13 https://github.com/arq5x/lumpy-sv/releases/download/0.2.13/lumpy-sv-v0.2.13.tar.gz
Manta 0.28.0 https://github.com/Illumina/manta/releases/download/v0.28.0/manta-0.28.0.release_src.tar.bz2
MaAsLin2 0.99.2 https://huttenhower.sph.harvard.edu/maaslin

Mitoseek 1.3 https://github.com/riverlee/MitoSeek
MSINGS 1.0 https://bitbucket.org/uwlabmed/msings/src/master/
MutationTimeR 0.99.2 https://github.com/gerstung-lab/MutationTimeR
MuTect 1.16 https://software.broadinstitute.org/cancer/cga/mutect_download
MuTect2 (for mitochondrial analysis) 4.1.4.1 https://software.broadinstitute.org/cancer/cga/mutect_download
MutPanning 2 https://github.com/vanallenlab/MutPanningV2
NeoPredPipe 1.1 https://github.com/MathOnco/NeoPredPipe
NorthStar 2.6.53.23
OncodriveCLUSTL 1.1.3 https://bitbucket.org/bbglab/oncodriveclustl/src/master/
OncodriveFML 2.4.0 https://bitbucket.org/bbglab/oncodrivefml/src/master
PathSeq 2018 http://software.broadinstitute.org/pathseq/Downloads.html
PCAWG SV merge 2020 https://hub.docker.com/r/weischenfeldt/pcawg_sv_merge
POLYSOLVER 1.0 https://software.broadinstitute.org/cancer/cga/polysolver_download
R 3.4.0 and 4.0.3 https://cran.ma.imperial.ac.uk/
SHAPEIT2 2.r904 https://mathgen.stats.ox.ac.uk/genetics_software/shapeit/shapeit.html#download
SigProfilerExtractor 1.1.3 https://github.com/AlexandrovLab/SigProfilerExtractor/releases/tag/v1.1.3
SigProfilerMatrixGenerator 1.2 https://github.com/AlexandrovLab/SigProfilerMatrixGenerator
smRegions 1 https://bitbucket.org/bbglab/smregions/src/master/
Strelka 2.4.7 https://github.com/Illumina/strelka/releases/tag/v2.4.7
Strelka (for immune escape prediction) 2.9.9 https://github.com/Illumina/strelka/releases/tag/v2.9.9
TelomereCat 3.3.0 https://github.com/cancerit/telomerecat
TelomereHunter 1.1.0 https://pypi.org/project/telomerehunter/
trackViewer 3.19 https://github.com/jianhong/trackViewer
UTRannotator 2020 https://github.com/ImperialCardioGenetics/UTRannotator
VEP 108.1 https://www.ensembl.org/info/docs/tools/vep/script/vep_download.html
Vegan 2.5-7 https://CRAN.R-project.org/package=vegan
xTea 1.1 https://github.com/parklab/xTea

For manuscripts utilizing custom algorithms or software that are central to the research but not yet described in published literature, software must be made available to editors and reviewers. We strongly encourage code deposition in a community repository (e.g. GitHub). See the Nature Portfolio guidelines for submitting code & software for further information.

Data

Policy information about availability of data

All manuscripts must include a data availability statement. This statement should provide the following information, where applicable:

- Accession codes, unique identifiers, or web links for publicly available datasets
- A description of any restrictions on data availability
- For clinical datasets or third party data, please ensure that the statement adheres to our policy

This is stated in the manuscript. Genomics England permits access to data used for this study subject to the following conditions. Research on the de-identified patient data used in this publication can be carried out in the Genomics England Research Environment subject to a collaborative agreement that adheres to patient led governance. All interested readers will be able to access the data in the same manner that the authors accessed the data. For more information about accessing the data, interested readers may contact research-network@genomicsengland.co.uk or access the relevant information on the Genomics England website: https://www.genomicsengland.co.uk/research. In order to expedite follow-on analyses, we have made available in the Genomics England Research Environment a 'Genomic Data Table' that provides for each patient and their tumour, all the individual clinical and molecular variable data used in this manuscript (see Supplementary Information Guide). It is recommended that those planning to access data consult the latest Genomics England regulations.

Research involving human participants, their data, or biological material

Policy information about studies with human participants or human data. See also policy information about sex, gender (identity/presentation), and sexual orientation and race, ethnicity and racism.

Reporting on sex and gender	Patients were not recruited to the study according to any sex- or gender-based criteria. Since colorectal cancer is more common in males, exploratory sex-specific analyses, or analyses using sex as a covariable, were performed throughout the study. Very few differences between the sexes were found as regards molecular variables and most results were therefore reported without respect to sex or gender. Some colorectal cancer driver genes are on the X chromosome and may in theory act differently in male and female patients.
Reporting on race, ethnicity, or other socially relevant groupings	We report the proportions of individuals of different self-reported and genetic ancestries in the study. A detailed analysis of differences with respect to ancestry is planned for a follow-up manuscript, but a preliminary assessment shows very few major differences.
Population characteristics	Any patient presenting with colorectal carcinoma to one of 13 Genomic Medicine Centres and their affiliated hospitals throughout England with was eligible for the study, subject to tumour sampling for molecular analysis being possible. Data are not available on the entire set of individuals invited to participate in the study. Participant characteristics are described in the manuscript. Median age at cancer sampling was 69 (range 23-94). 41% participants were female. Samples comprised 1898 primary carcinomas, 122 metastases from primary colorectal cancers, and 3 recurrences. Nineteen individuals had an unreported Mendelian cancer syndrome. We estimated that 90.2% patients were of European ancestry, 2.6%` African, 0.7% East Asian, 3.2% South Asian and 3.3% mixed. Age, sex, treatment, germline genetics and the presence of co-morbidities or family history were not factors listed as relevant in patient recruitment. Cancer patients treated successfully with

	neoadjuvant therapy may be under-represented owing to a very small cancer or impure sample following that therapy.
Recruitment	Participant recruitment was by NHS staff. Recruitment was open to all patients with colorectal carcinoma who were able to provide informed consent. Small biases are likely based on patient willingness to take part in research, and also clinical features (e.g. patients presenting as emergencies were likely to be under-recruited).
Ethics oversight	Ethical approval was provided to the 100,000 Genomes Project by the HRA Committee East of England – Cambridge South research ethics committee (REC Ref 14/EE/1112). Samples were obtained as part of the 100kGP cancer programme, an initiative for high throughput tumour sequencing for NHS cancer patients. Patient recruitment was organised by 13 Genomic Medicine Centres (GMCs) and their affiliated hospitals across England. All patients provided written informed consent. Study oversight was subsequently undertaken by Genomics England through regular reporting updates to the GeCIP steering committee and data Airlock committee.

Note that full information on the approval of the study protocol must also be provided in the manuscript.

Field-specific reporting

Please select the one below that is the best fit for your research. If you are not sure, read the appropriate sections before making your selection.

[x] Life sciences   [ ] Behavioural & social sciences   [ ] Ecological, evolutionary & environmental sciences

For a reference copy of the document with all sections, see nature.com/documents/nr-reporting-summary-flat.pdf

Life sciences study design

All studies must disclose on these points even when the disclosure is negative.

Sample size	Sample size was determined by the recruitment achieved by NHS staff, by availability of tumour and matched normal samples for DNA extraction, and by quality control thereafter in terms of DNA extraction. In addition, some samples were excluded from copy number analysis owing to failure to establish a fit to reported purity metrics.
Data exclusions	Exclusions were based on low sample purity, standard sequencing quality metrics, and availability of clinicopathological data (for sub-studies). Specific sequence data were excluded from regions of duplications or repeats, low mappability, or seuencing chemistry errors (e.g. strand bias). All criteria were based on standards or norms in the field, although some additional exclusions were made ad hoc based on our own findings.
Replication	Comparisons with previous work in the field were performed wherever possible. Almost all the common colorectal cancer driver mutations and copy number alterations found by other studies were also found by us, and there was overlap with previously reported mutational signatures. However, we only replicated ~7% of previously reported drivers and some signatures were present at much higher frequencies or absent in our data compared with other data sets. We make relevant comparisons with previous data at various points in the manuscript. Since some of our discoveries were of uncommon mutations or cancer sub-groups, we did not sub-divide our study into test and validation patient sets. We did, however, test the stability of mutational signatures and derived clusters by analyses of random sub-sets of the data.
Randomization	This was not an intervention-based study and hence randomisation is inappropriate.
Blinding	N/A. The study has no assessments or procedures that are appropriate for blinding.

Reporting for specific materials, systems and methods

We require information from authors about some types of materials, experimental systems and methods used in many studies. Here, indicate whether each material, system or method listed is relevant to your study. If you are not sure if a list item applies to your research, read the appropriate section before selecting a response.

Materials & experimental systems

n/a	Involved in the study	
[x]	[ ]	Antibodies
[x]	[ ]	Eukaryotic cell lines
[x]	[ ]	Palaeontology and archaeology
[x]	[ ]	Animals and other organisms
[ ]	[x]	Clinical data
[x]	[ ]	Dual use research of concern
[x]	[ ]	Plants

Methods

n/a	Involved in the study	
[x]	[ ]	ChIP-seq
[x]	[ ]	Flow cytometry
[x]	[ ]	MRI-based neuroimaging

Clinical data

Policy information about clinical studies

All manuscripts should comply with the ICMJE guidelines for publication of clinical research and a completed CONSORT checklist must be included with all submissions.

Clinical trial registration	N/A
Study protocol	This is described in https://www.bmj.com/content/361/bmj.k1687
Data collection	Within Genomics England Genomic Medicine Centres and their satellite hospitals, with central data collection by Genomics ENgland core team.
Outcomes	Certain studies have utilised overall survival as an outcome. Other outcomes include fundamental measures found on the histopathological reporting proforma for colorectal malignancy, e.g. stage.
