# Supplementary material for: The genomic landscape of 2,023 colorectal cancers
Source: Nature. 2024 Aug 7;633(8028):127–36. doi: 10.1038/s41586-024-07747-9 (PMC11374690; doi:10.1038/s41586-024-07747-9)
Supplement: Supplementary file 3 — This file contains Supplementary Results 1–9, Supplementary Note, Supplementary Figs 1–11 and Supplementary References. [file 41586_2024_7747_MOESM3_ESM.docx]

**SUPPLEMENTARY RESULTS**

**Supplementary Result 1. Co-occurrence of mutational signatures and other molecular changes**

Many signatures co-occurred with specific driver mutations (**Fig. 2a; Extended Data 1d**). In some cases, the likely underlying factor was over-representation of the driver gene in MSS, MSI or POL cancers (**Supplementary Tables 4-7**). In an MSS-only analysis, *TP53* mutations were positively or negatively associated with 17 signatures of all types, but particularly with copy number signatures (**Extended Data 2b**), reflecting various types of chromosomal-scale aberrations plausibly resulting from, or permitted by, p53 deficiency. SBS18 was associated with *KRAS* and *BRAF* mutations, suggesting endogenous oxidative DNA damage perhaps resulting from altered cancer metabolism. Of the SBS signatures without a known aetiology, SBS17a/17b were associated with multiple driver mutations (*PIK3CA, BRAF, TNF43, FBXW7* and *SMAD4*) and SBS93 with *APC, TP53* and *B2M* changes. Higher activities of SV4 (fragile site deletions; *P*=6×10^-8^), SV8 (unbalanced inversions; *P=*6×10^-9^) and SV9 (unbalanced translocations; *P*=3×10^-9^) ^1^ were associated with *TP53* mutation. SV5 (small tandem duplication) and SV6 (early replicating medium tandem duplication) activities correlated with *FBXW7* mutation (*P*=5×10^-5^ and *P*=6×10^-6^ respectively), concordant with observations in other cancers ^2^. Additionally, SV1 (small deletions; *P*=1×10^-9^) correlated with mutation of *ATM*, consistent with a causal aetiology resulting from defective double-strand break repair ^3^.

Many SV and CN signatures co-occurred, as expected given their overlapping molecular features. The strongest associations (all P<10^-8^) were between unbalanced inversions and translocations (SV8 and SV9) and CN17 (HRD), and between SV3 (mid-size deletions) and CN9 (CIN on a diploid background). The strongest associations of SV and CN signatures with SBS/DBS/ID signatures all involved SV4 (medium-sized deletions), which co-occurred with SBS93 and SBS17b, and was negatively associated with SBS5 (all P<10^-8^).

**Supplementary Result 2.** **Selected driver gene mutations identified in primary CRCs.**

Coloured circles represent mutations predicted as oncogenic and are annotated with their predicted consequence. Faint circles represent variants of unknown significance.

(a) Non-synonymous mutations in driver genes identified in MSS primary tumours.


(b) Non-synonymous mutations in driver genes identified in MSI tumours

(c) Non-synonymous mutations in driver genes identified in primary POL tumours

**Supplementary Result 3. Ras-Raf-Mek-Erk pathway driver mutations**

The new driver genes extended the role of the Ras-Raf-Mek-Erk and broader MAP kinase pathway in CRC (**Extended Data 2d**), including five MAP2 or MAP3 kinase driver genes, mostly involved in Jun kinase activation and signalling to Mek ^4^. Other new Ras-pathway drivers included the Ras activator *RASGRF1* (recurrently mutated in its RhoGEF domain), *RAF1* (hotspot p.Ser257Leu, a site mutated in the germline of Noonan syndrome patients), and Ras suppressor *RASA1* (primarily loss of function changes). These mutations were frequently accompanied by LOH (**Supplementary Table 18**), conventionally implying loss of function, even though some of the genes are predicted pathway activators. None of the new Ras-related drivers were mutually exclusive (*P*>0.05) with an established, major Ras driver (*KRAS, NRAS* or *BRAF*)*,* suggesting that the former act as modifiers and/or in a different branch of the MAP-kinase pathway. An exemplar novel MSI driver in the MAPK pathway was the GTPase *RGS12,* principally inactivated by indels at two short repeats (K1178delC in 29% cases and P1290delA in 25% cases). *RGS12* inactivation is likely to enhance MAPK signalling ^5,6^ and frequently co-occurred with *BRAF* V600E. Whilst recurrent passenger indels are common in MSI CRCs, additional support for *RGS12*’s driver status was that: (i) biallelic mutations were relatively common; (ii) ~10% MSI cancers had non-hotspot *RGS12* frameshift mutations (e.g. P869delA); (iii) *RGS12* was also inactivated by nonsense mutations (3 MSI and 3 MSS); and (iv) the functional homologue *RGS7* is a driver in POL cancers. Evidence for *RGS12* driver status and assessment of other hotspot mutations in MSI cancers is detailed in **Supplementary Table 7.**

**Supplementary Result 4. SVs underlie some chromosomal-scale CNAs**

In MSS primary tumours, chromothripsis and complex SVs were enriched on chromosomes 8, 17, 18 and 20 (**Extended Data 3b,c**). The non-fragile site having the greatest complex SV enrichment was chr8:28-62Mb, which contained a chromothripsis or unclassified complex SV in 9% and 18% of MSS and MSI cancers respectively. Complex SVs at this site were associated with arm-level somatic copy number alterations (CNAs) on the same chromosome (**Supplementary Table 12**), suggesting that the former can underlie the latter changes. Most notably, MSS primary cancers with arm-level 8p deletion (*P=*9×10^-32^), 8q amplification (*P*=2×10^-19^), and 20q amplification (*P*=1×10^-16^) were enriched for complex SVs on the respective chromosome. Specifically, 44% of cancers with arm-level 8p deletions had a complex SV at chr8:28-62Mb, compared with 12% without those deletions.

**Supplementary Result 5. Clinical actionability**

Using OncoKB and COSMIC Mutation Actionability in Precision Oncology databases, genetic alterations for which an approved therapy is indicated were identified in 25% (497/2,023) of cancers (**Supplementary Table 20**). The most frequent actionable genetic features were high tumour mutational burden (21%), MSI (18%), and *BRAF* V600E (14%). Genomic features with compelling evidence for predicting response to a therapy, as annotated by OncoKB, were present in 64% (1,304/2,023) of cancers (**Supplementary Table 21**). Data on druggability were available for 214/242 CRC drivers (**Supplementary Table 22**): 26 had approved therapies and 84 had an identified quality probe or were deemed ligandable. Of the remainder, four had an “investigational” status, leaving nine that may be viable targets for novel therapeutic intervention. Based on DepMap data, 15 of the genes are predicted to have very good evidence (efficacy<-1.5) of being essential, with positive selectivity” ^7^. Of these genes, two have approved therapies and eight are deemed ligandable.

It is unclear whether the new, rarer driver genes represent good therapeutic targets. On the one hand, rare drivers such as *IDH1, IDH2, BRCA1* and *BRCA2* are already therapeutic targets, yet other evidence – such as the low frequency of second hits for most of the less common drivers – raises concerns that the effects of such mutations are generally modest, perhaps optimising existing pre-dysfunction, and that their therapeutic targeting could have correspondingly limited effects.

**Supplementary Result 6. Stage, survival and molecular variables**

In multivariable analysis of MSS primary cancers, tumour stage, a major determinant of survival, was associated with higher frequencies of arm-level 4p (*P*=8x10^-4^) and 8p deletions (*P*=8x10^-4^) (**Supplementary Table 34**) ^8^. Greater SV (*P*=9×10^-5^) and CNA (*P*=1×10^-5^) numbers were also observed in later-stage cancers.

We compared genomic differences between MSS primaries and unpaired MSS CRC metastases. Almost all of the metastases had received radiotherapy or chemotherapy. Although there was no significant difference in TMB (Wilcoxon signed rank test; *P=*0.073), metastatic cancers tended to have higher ploidy (Wilcoxon signed rank test; *P=*3.67x10^-5^), consistent with reports of frequent whole genome doubling in metastatic CRC ^9^. Comparing CNAs between metastases and primaries based on 1Mb bins identified no significant differences, but when copy number was considered relative to mean ploidy, several regions differed significantly between primaries and metastases (Q<0.05). Notably, the 8p23-8p12 region had a lower copy number in metastases, consistent with reports of 8p as a ‘metastatic susceptibility locus’ ^10^. Whilst differences in immune pressure between primary and metastatic CRCs might be expected (**Fig. 3; Extended Data 7**), we detected no significant difference in immune escape frequency (*P=*0.142), or neoantigen burden (*P=*0.44). Six genes (*CDK12, CUL4A, FAT3, MET, PRKCB* and *WNK2*) were identified as candidate drivers only in the metastasis-MSS group (**Supplementary Tables 4 & 35**). These mutations could be markers or act directly in several ways, increasing the propensity to metastasise, driving tumorigenesis only in extra-colonic sites, or conferring resistance to anti-cancer therapy.

In MSS primary cancers without prior therapy (n=894 with survival data, 144 deaths), poorer overall survival (OS) was associated with higher tumour stage, greater age, and proximal anatomical location. Accounting for age, sex, tumour stage and anatomical location, higher global SV burden and presence of an *NRAS* mutation were the only molecular features associated with OS (*Q*<0.05). There was also borderline better survival for immune escaped tumours in a similar analysis of the MSI group (*Q*=0.051).

**Supplementary Result 7. Five exemplar analyses of rare molecular sub-types of CRC**

Our patient sample size provided an opportunity not only to add to the data on existing rare sub-groups of CRC, (e.g. new driver genes in POL cancers), but also to identify or characterise other rare molecular sub-groups of CRC. Five exemplars are presented here.

(i) Driver SNVs and indels in CRC have been shown to be monoclonal and present from an early stage of tumorigenesis ^11^, in contrast to some other cancer types. Our data generally supported this notion. We found that 97% of MSS primary driver mutations were clonal, compared with 89% in primary MSI tumours (*P*<2.2x10^-16^; **Supplementary Table 24**) ^12^. The allele frequencies of these mutations were slightly higher in MSS than MSI cancers (means 0.154 *v* 0.123, *P*=0.0005, t test). Although some of the excess of sub-clonality in MSI tumours could result from genetic drift alone, the changes we see more often have predicted functional effects, suggesting a model in which sub-clonal drivers tend to confer small selective advantages, arise more often in hypermutant cancers, and often fail to cause a selective sweep ^13^. In general, driver genes with higher proportions of sub-clonal mutations had lower mutational frequency (for MSS primary, Spearman's rho = 0.17, *P*=0.038). Sub-clonal driver mutations in MSS tended to be isolated (*i.e.* no other drivers existed in the sub-clone), whereas MSI sub-clones tended to have multiple drivers (42/166 MSS *v* 179/215 MSI, *P*<0.001, Fisher’s exact). Among major MSS drivers (mutation prevalence >5%), three genes – *SMAD4* (13%, 18/135), *ZFP36L2* (5.7%, 6/105) and *PIK3CA* (5.7%; 14/253) – had the highest proportion of sub-clonal mutations. Respective frequencies in MSI tumours were 17%, 20% and 10%. *PIK3CA* is known to occur sub-clonally, plausibly because its selective advantage is weak ^13^, whereas *ZFP36L2* and *SMAD4* are drivers in several cancers, yet have not been noted to be sub-clonal. After excluding some mutations with unclear functional importance (*e.g*. very low allele frequency changes), we found five CRCs in which a sub-clonal *SMAD4* mutation co-existed with a sub-clonal 18q deletion, and one tumour in which two mutations were sub-clonal (on the same parental chromosome but in different cells). Arm-level 18q deletion, thought principally to target *SMAD4,* is present in ~90% of MSS CRCs and is accompanied by chromosomes carrying clonal *SMAD4* mutations in ~10% of cases. The deletions usually caused allelic loss, with one or two copies remaining of the retained 18q, but in a few cancers, we found independent presence in different sub-clones of a pathogenic *SMAD4* mutation or sub-clonal loss of a remaining chromosome 18 homologue (**Extended Data 6a**) – this was thus an unusual example of parallel evolution of a copy number changes and a point mutation, both presumably targeting *SMAD4*. These sub-clones persisted as the cancer grew and were accompanied not by additional sub-clonal driver SNVs/indels, but by additional sub-clonal copy number changes. Parallel evolution has been regarded as rare in CRC, but actually appears to involve at least two major drivers, *PIK3CA* and *SMAD4/*18q deletion*.*

(ii) The ‘major’ Wnt drivers (*APC, RNF43, CTNNB1* and R-spondin fusions) were generally mutually exclusive, but sometimes co-occurred with other rarer, ‘minor’ Wnt drivers (*TCF7L2, SOX9,* *AMER1, AXIN1, AXIN2, BCL9* and *BCL9L;* **Extended Data 6b**). We found 1.8% of CRCs to carry activating *CTNNB1* (β-catenin) exon 3 mutations. *CTNNB1* was a rare driver in both MSS and MSI cancers, with over-representation in the latter (12/1641 MSS v 24/364 MSI, *P*<0.001, Fisher’s exact test). Twenty-one of the 36 *CTNNB1-*mutant cancers with pathogenic mutations also had mutations in the minor Wnt driver, *TCF7L2* (*P*<0.01, logistic regression including MSI, location and age). It has long been assumed that, as an oncogene activated by missense mutations, *CTNNB1* becomes a driver through monoallelic mutations and does not require a second hit. However, *CTNNB1* showed almost universal second hits, mostly by copy-neutral LOH (12/12 MSS and 22/24 MSI tumours), plausibly owing to selection for increased dosage of the mutant and/or absence of the wildtype allele (**Supplementary Table 18**) ^14,15^. Since this association was present in MSS cancers as well as MSI, it was not necessarily related to the co-occurrence of driver mutations in the MMR gene *MLH1,* which is located on chr3p close to *CTNNB1*. *CTNNB1-*mutant cancers presented about 8 years younger than other tumours, whether MSS or MSI (*P*=2.7x10^-3^ and *P*=9.2x10^-4^ respectively). No CRCs with distant metastases carried *CTNNB1* mutations (*P*=0.027, Fisher’s exact test), consistent with better survival of this group as per our earlier work ^13^.

(iii) Whilst it is established that not all MSS CRCs have multiple chromosomal changes, there have been only occasional reports of MSI cancers with chromosomal instability (MSI CIN). After stringent quality control to exclude any type 1 errors of MSI or CIN/WGD calling, we identified eight MSI cancers with WGD (0.5%), all with multiple, often polyclonal arm-level CNAs (**Extended Data 6c**). In these tumours TMB was variable (range 3.1-189/Mb) and SBS1 (clock-like) activity low (6% v 12% MSI and 16% MSS, *P*<0.01, Kruskal-Wallis test). Our observations support MSI CIN being a distinct, rare subgroup of CRC, possibly associated with low CpG methylation.

(iv) The role of HRD in CRC is debated. Using HRDetect ^16^, we identified HRD in 12 (0.7%) cancers. Three had pathogenic germline *BRCA1* mutations accompanied by loss of the wild-type allele, and three had biallelic mutation or homozygous deletion of *BRCA2* (**Extended Data 6d**). The remaining six HRD-positive cancers had no clear underlying cause. We found no evidence that the *BRCA1/2* mutations were driver changes in those cancers, but even if these were bystander changes, our findings raise the prospect of therapeutic PARP inhibition in this rare subset of CRC patients.

(v) Two hundred and nineteen patients had received prior genotoxic therapy for current CRC or a non-CRC cancer. Colorectum-targeted radiotherapy (RT), generally applied in the neoadjuvant setting, was associated with higher ID8 signature activity, as previously reported (*P=*5×10^-8^; **Extended Data 6e**) ^17^. ID8 was not, however, a feature in 31 metastases with prior colorectum-targeted RT, consistent with secondary spread occurring prior to RT. Neoadjuvant oxaliplatin treatment was associated with higher DBS5 activity (*P*=8×10^-5^) ^18^, but other signatures associated with platinum-based therapy, including SBS31, were not detected ^19^. DBS5 was observed in both primary tumours and metastases, and for both RT and oxaliplatin, treatment duration (*P*=0.004 and *P*=0.006) and time since treatment (*P*=2×10^-6^ and *P*=1×10^-3^) correlated with respective signature activity. SBS17b, reported to be associated with 5FU-therapy ^9^, was not detected in prior-treated cancers. RT for prostate cancer is associated with risk of second malignancy, ^20^. Twenty-eight patients with RT-naive CRC had previously been treated for prostate cancer by RT and in these ID8 activity was elevated (*P*=3×10^-13^), consistent with induction of mutations in CRC progenitors by treatment. However, this association occurred despite only 5 (18%) of these cancers showing evidence of ID8 activity (proportion of all ID mutations 5.9-18.6%). Furthermore, these cancers showed with no excess of ID8-associated driver mutations in the major TSGs, *APC, SMAD4* and *TP53* (*P>*0.1)*.* It remains possible that RT for prostate cancer is partly or wholly causal for CRC through mechanisms other than direct mutagenesis through ID*-related processes, potentially including an excess of a variety of mutation types or non-genetic mechanisms, such as tissue repair.

**Supplementary Result 8. Four exemplar analyses of the non-coding and/or non-nuclear CRC genome**

(i) We used OncodriveFML ^21^ to identify potential driver mutations in regulatory non-coding elements in MSS Primary cancers (**Methods**). There was some evidence of enrichment of mutations in functional sequence, notably promoters and transcription factor binding sites, with 85 specific non-coding elements significantly enriched for mutations (Q<0.01; **Supplementary Table 25**).

(ii) Three sites were identified as focal CNA regions, yet contained no coding genes. All comprised deletions, occurred in MSI cancers, and were found to be partly overlapping SV hotspots (**Supplementary Tables 10 & 16**). The first and second regions (chr6:147606235-147667515 and chr7:110460281-110511282) include spliced EST ENST00000566741.1 and lincRNA AC003088.1 (LOC105375451) respectively. The latter is expressed in the colorectum (https://www.gtexportal.org/home/gene/ENSG00000226965), but its role is undetermined. The third region, chr17:72429007-72450223, contains lincRNA LINC00673 (LINC00511), a transcript with evidence of a role in carcinogenesis. LINC00673 interacts with EZH2 ^22,23^, which we have found to be an SV CRC driver (**Supplementary Table 35**), and reportedly can act as a tumour suppressor by promoting interaction between PTPN11 ^24^, a driver in MSI CRCs (**Supplementary Table 4**), and ubiquitin ligases. A nearby polymorphism, rs983318 (chr17: 72,417,112), is associated with CRC risk in genome-wide association studies ^25^. This region overlaps with an SV deletion hotspot (chr17:72,228,421-72,770,582) in MSS primary tumours that includes a regulatory element interacting with the promoter of the nearby CRC driver *SOX9* (**Extended Data 6f**).

(iii) We examined neo-splice site mutations, representing non-coding events most tractable to immediate analysis. We found *APC* neo-splice site mutations in 136 cancers, including c.835-8A>G in 111 cancers (**Supplementary Table 26**). The pattern of second hits at *APC* showed statistically that the neo-splice site mutations acted as loss-of-function changes equivalent to canonical protein-truncating mutations. Ten predicted pathogenic neo-splice site *SMAD4* mutations were identified, including five at c.788 (**Supplementary Table 26**). All were accompanied by LOH second hits, strongly suggesting functionality.

(iv) We examined the mitochondrial genome. The distribution of C>T substitutions on heavy and light mitochondrial DNA strands supported the theory that processes driving mtDNA mutation in CRC are coupled to replication rather than transcription ^26^. In MSS Primary cancers, there was positive selection of several somatic changes, notably missense and truncating mutations in MT-CYB (encoding cytochrome B) and missense mutations in the NADH dehydrogenase genes *MT-ND1, MT-ND2, MT-ND3, MT-ND4* and *MT-ND4L* (Q<0.05; **Supplementary Table 27**). The importance of mitochondria to tumorigenesis was further emphasised by the identification of nuclear genes with mitochondrial functions as CRC drivers (**Supplementary Table 4**). For example, DNA polymerase *POLG* was identified as a driver gene in MSI cancers. Although this might lead to mitochondrial hypermutation, mtDNA mutational burden did not differ between *POLG*-mutated and wild-type cancers (Wilcoxon test, *P*=0.417).

**Supplementary Result 9. Micro-organisms and the non-human CRC-associated genome**

An analysis of the CRC-associated microbiome was performed based on cancer sequencing reads mapping to bacterial and viral reference genomes. After removal of likely hospital laboratory contaminants (**Extended Data 8a**), we identified multiple bacterial species, predominantly from the genera *Bacteroides*, *Fusobacterium*, *Shigella*, *Streptococcus* and *Prevotella* (**Supplementary Tables 28-30; Extended Data 8b**). We also detected a small number of human viruses, mostly *Herpes* species, and multiple phage species. No associations between microbial content and HLA alleles was demonstrated (*P*>0.05). Considering bacterial load and diversity (**Extended Data 8c,d**), 98.5% of primary tumours had >0.001 bacterial cells per human cell, but in contrast to previous work ^27^, most metastases had almost no microbial content. Considering only MSS tumours, bacterial load decreased transitioning from proximal colon to rectum, and diversity was greater in the colon than rectum. MSS cancers had fewer bacteria (*P*=9×10^-12^), but greater diversity (*P*=4×10^-9^), than MSI cancers. Anatomical site of the tumour and MSI status had the strongest associations with bacterial diversity. In multivariate analyses of common taxa, several associations with CRC type and location were found. For example, *Fusobacterium* were more abundant in MSI than MSS cancers (*Q*=5×10^-5^), and *Akkermansia*, *Roseburia* and *Prevotella* more abundant in proximal than distal CRCs (*Q*=5x10^-5^, *Q*=2×10^-6^ and *Q*=6×10^-4^ respectively; **Extended Data 8e;** **Supplementary Tables 28-30**). pks+ bacterial prevalence was not associated with SBS88 or ID18 activity, consistent with effects earlier in life (**Extended Data 8f**). Signature DBS6 (TG>NN) was associated with 0.15% of genus-level variation (*P*=0.005) and ID8 (longer deletions at repeats with microhomology) was associated with 0.25% of variation (*P*<0.001). Of the more common genera, *Porphyromonas* (*Q*=0.0001), *Fusobacterium* (*Q*=0.0038) and *Streptobacillus* (*Q*=0.037) were all found at significantly higher levels in ID8-positive samples.

**Supplementary Note: Extended synopsis of study**

Herein we provide the largest and most comprehensive analysis of the genetic landscape of CRC to date based on 2,017 patients recruited to the UK 100,000 Genomes Project. A principal strength of our study is the power to detect uncommon features, including mutational drivers, SVs, copy number alterations, mutational signatures, and genomic complexity. Based on these data we are also able to define and characterise specific patient sub-groups. Although the lack of gene expression data is a limitation, the availability of WGS has allowed us to integrate multiple types of mutation data and identify associations with clinicopathological variables.

The CRC genome is principally shaped by base substitution and indel mutations derived from imperfect DNA replication or spontaneous DNA modifications, such as deamination. Potentially modifiable mutational processes with lesser, but significant, activity include SBS18 (ROS) and DBS2 (tobacco smoking). Acquired defects in replication-coupled repair (MMR deficiency, *POLE*) are super-added in important, well-described sub-sets of CRCs in which many driver mutations bear the mutational hallmarks of those specific defects. SVs and focal CNAs contribute relatively few mutations alongside substitutions and small indels, although such mutations remain important and a small number of likely driver TSGs and oncogenes are only mutated by SVs and/or focal SCNAs.

Our analysis has significantly expanded the list of putative CRC driver genes to nearly 250 by combining the analyses of small substitutions and indels, SVs and focal CNAs. Most drivers are present in <5% of CRCs, or are specific to a non-MSS sub-group. Some of these genes appear to be specific to cancers arising in particular anatomical locations within the large bowel. The overlap between small-scale mutations and putative driver SVs was strong, with most putative SV target genes also identified as drivers in the small-scale mutation analysis. TSGs inactivated by all three classes of mutation included *ACVR2A, APC, B2M, PTEN,* and *SMAD4*. *SMAD4* provides an example of parallel evolution of protein-inactivating driver point mutations and CNAs, a phenomenon generally thought to be rare within CRC. Our new CRC drivers will require validation in other studies, but our failure to validate over 90% of previously reported CRC driver genes suggests that this will be an ongoing process and some of our candidate drivers will not be shown to be true drivers. Given that new high-frequency drivers will very rarely be detected in the future, we believe it likely that real drivers will be derived using a consensus approach, starting with lists from WGS, and subsequently supported by multiple strands of evidence, including other genomic data and functional studies.

Using clustering methods, we found four molecular sub-groups of MSS primary CRC, in addition to the established MSI and POL groups. These new sub-groups are characterised by a variety of molecular features, including: WGD; SV and CNA burdens; *KRAS* and *TP53* mutations; and signatures SBS18, SBS88, SBS93, ID18 and CN9. The largest cluster (MSS-WGD-B) generally has features of ‘classical’ MSS CRCs, but the three other clusters respectively combine severe CIN with *RNF43* and *BRAF* mutations (MSS-WGD-A), have MSI-like features but normal SNV and indel burdens (MSS-GS), or have a form of CIN characterised by focal loss of heterozygosity (MSS-LOH). The clusters have potential prognostic associations and share some features with CRC groups reported by others ^28,29^, supporting their validity.

Nested within the six major clusters of primary CRC are rare groups of patients, such as tumours with uncommon Wnt drivers, HRD, or both chromosomal and microsatellite instability. Whilst we have identified multiple new potential therapeutic targets for CRC through driver discovery, it is arguably rare sub-groups for which there are existing targeted therapies who will benefit most in the short term.

Our data indicate that hypermutated CRCs have near-universal immune escape driver mutations, often by impairing antigen presentation, whereas some MSS CRCs have weaker immune escape mechanisms, mostly caused by HLA copy number changes. It will be interesting to determine the extent to which these mutations limit response to therapies that inactivate immune checkpoints. Immune editing appears to be frequent. Using TMB as a covariate, we have decoupled the effects of neoantigen burden and TMB, thus providing additional insights into immuno-editing. The strength of immune surveillance appears highest in the distal colorectum. Perhaps related to this, CRCs from more distal sites have more SVs and CNAs, but fewer SNVs and indels. The frequencies of some major driver genes and mutational signatures also vary monotonically along the large bowel from caecum to rectum, independent of MSI status. Perhaps surprisingly, in MSS cancers, we found that SNV and SV burden are not associated with age, but indel burden is highest in the youngest and oldest patients. Several mutational signatures were age-associated, but none has an established aetiology that could explain the recent rise in early-onset CRC.

Signature SBS93 has been a recurrent feature in our work. It is the fourth most common SBS signature (after SBS1, SBS5 and SBS18) in our samples, yet was not linked to CRC until very recently, and has not been accorded any importance in CRC until now. It is associated with younger patients, proximal location, MSS cancers and signature ID14. It also featured as a discriminant in our discovery of MSS clusters. Given that SBS93 is also prominent in oesophageal squamous and gastric cancers, has been linked to smoking at the behavioural level, and shows features associated with by-pass of DNA adducts by error-prone polymerases such as Rev1, Rev3L and Pol, we speculate that the signature arises from alcohol, other dietary components, or perhaps the microbiome. Further investigation of SBS93, ID14 and their correlates appears warranted.

The benefits of WGS in practice lie mostly in consistency of the analytical pipeline, enhanced detection of driver mutations (including intronic and chromosomal-scale changes), and better measurement of whole-genome metrics, such as mutational signatures and burdens. There is also the potential for assessment of features such as mitochondria, the microbiome and other forms of non-nuclear DNA. Future research studies, especially of the non-coding genome, will require even larger sample numbers. For clinical purposes, we have found WGS to identify a few large CRC sub-groups with different behaviours (MSI, POL and a small number of MSS clusters), and some less common groups defined by rare driver mutations or mutational processes. It is important that these genomic classifiers, or surrogates, are fully assessed in the diagnostic setting for their utility in patient management.

**SUPPLEMENTARY FIGURES**


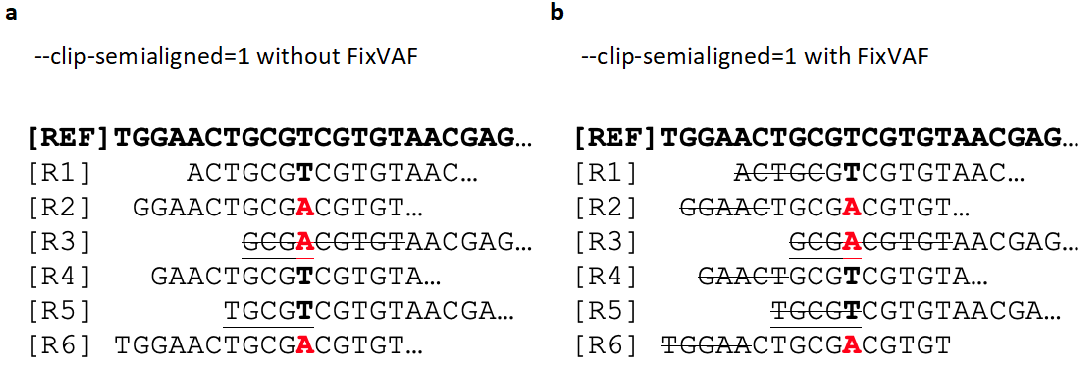


**Supplementary Figure 1.** **Removing bias introduced by soft clipping of semi-aligned reads.** An alignment of six reads to a reference sequence containing an A/T variant is shown. Bold black T and red A represent reference and alternate alleles respectively. Soft clipping is represented by strikethrough. Without soft clipping, three reads would support both the reference (T) and alternative (A) alleles, resulting in an unbiased variant allele frequency (VAF) of 3/6=0.5. (**a**) Read R3 is soft clipped until five consecutive matches with the reference are obtained. After clipping, only two reads support the alternate allele (A), whilst three reads support the reference allele (T), resulting in a biased VAF of 2/5=0.4. (**b**) FixVAF clips all reads by five bases, regardless of whether they contain a variant site or support a reference or alternate allele. Reads supporting both the reference and alternate alleles are now clipped by five bases. In this example, FixVAF would compute a VAF of 2/4=0.5, and therefore remove bias.


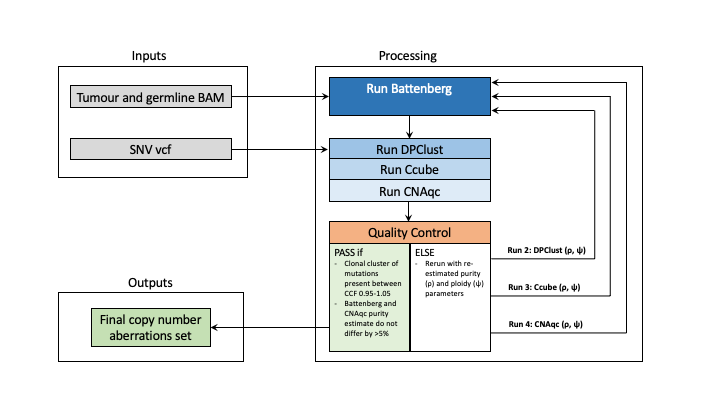


**Supplementary Figure 2. Overview of copy number aberration calling pipeline.** BAM: binary sequence alignment map, CCF: cancer cell fraction, SNV: single nucleotide variant, vcf: variant call format.

**Supplementary Figure 3. Overview of structural-variant-calling pipeline.** BAM: binary sequence alignment map, PCAWG: The Pan-Cancer Analysis of Whole Genomes, SV: structural variant.

**
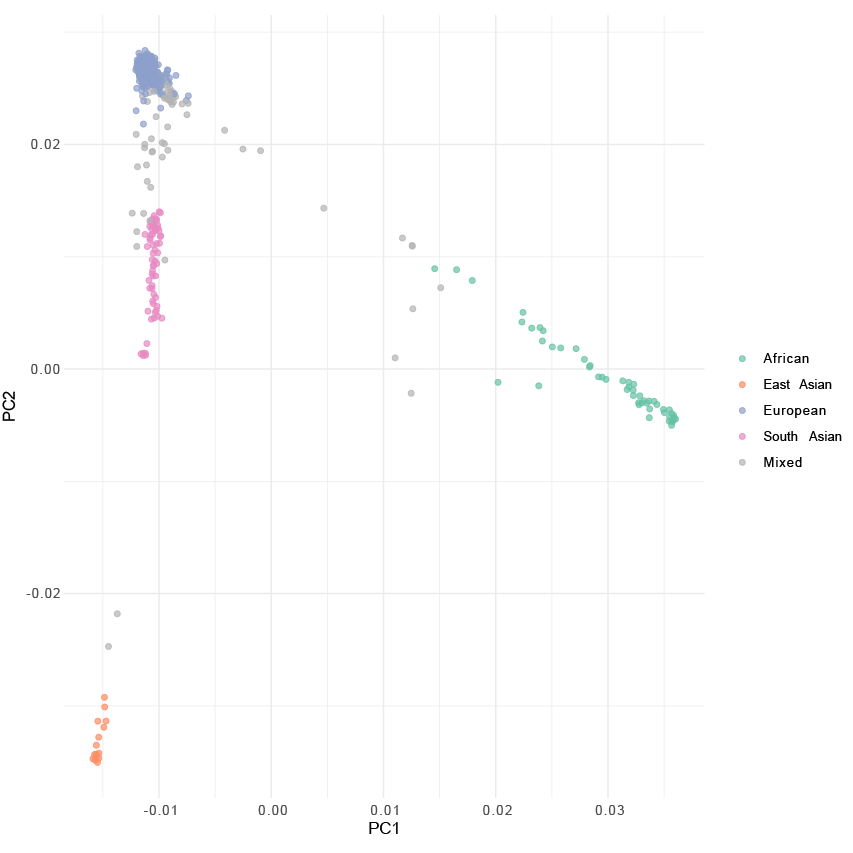
**

**
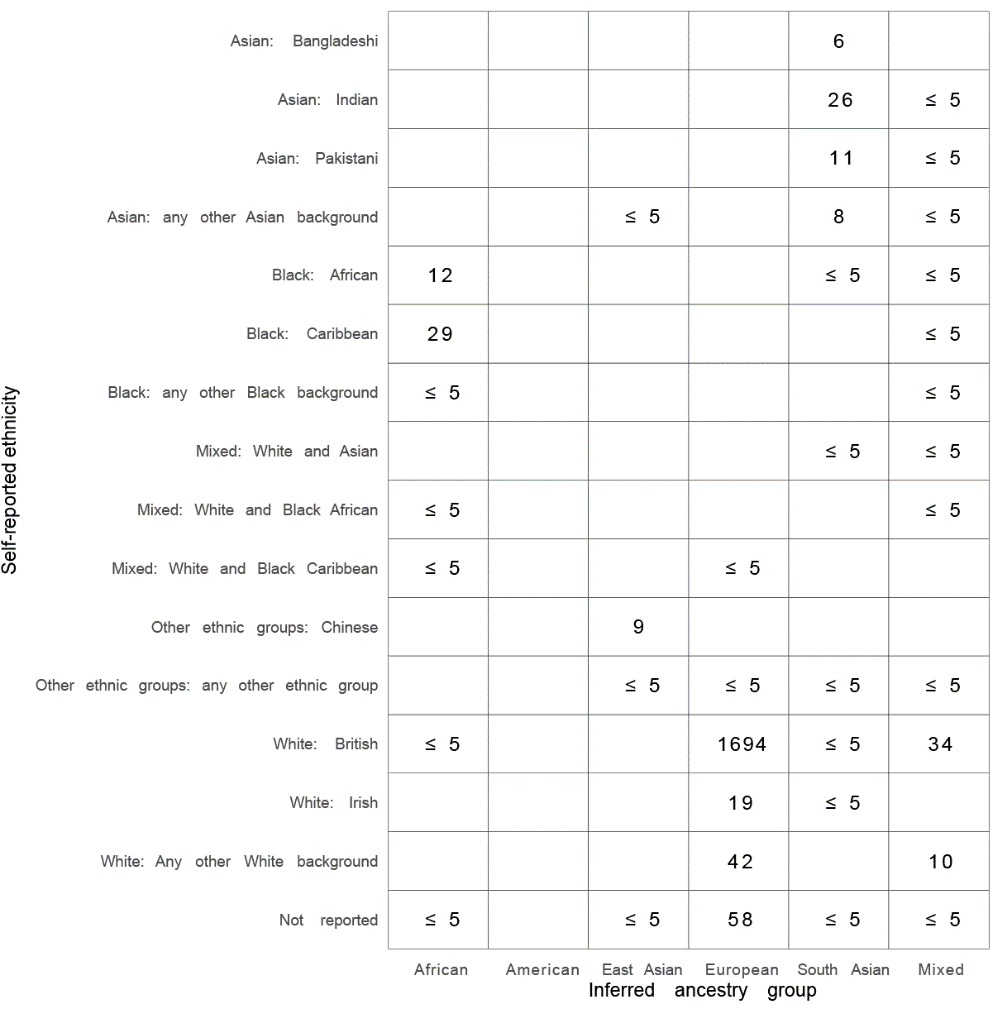
**

**Supplementary Figure 4. Ancestry.** Upper, principal component analysis of 2,017 CRC patients showing major European, smaller clusters of Asian and African ancestry, and individuals of mixed ancestry outside the major cluster. Lower, high correspondence between 16 self-reported ancestry groups and PCA classification.

**Supplementary Figure 5. Association between SNV VAF and numbers of driver mutations called in primary microsatellite stable (MSS) colorectal cancer (CRC) samples (n=1956), stratified by median single nucleotide variant (SNV) variant allele frequency (VAF).** Potential driver mutations were defined as any coding variant annotated as probably pathogenic from 63 driver genes previously identified in MSS CRC^2,3,4,7^ ^3,30-32^. To the left of the dashed line, it is likely that low median VAF results from low cancer cell purity and this causes a failure to identify some driver mutations. Tumour samples below the threshold were therefore excluded from the analysis.

**
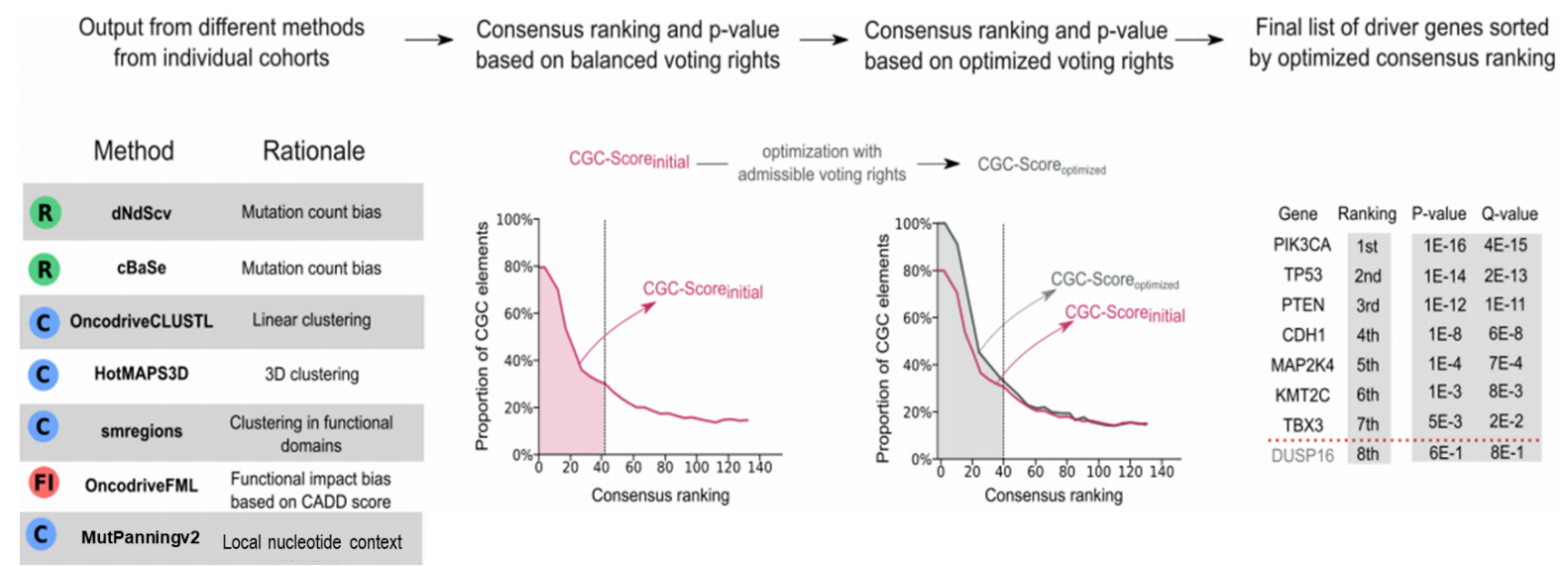
**

**Supplementary Figure 6. IntOGen pipeline.**


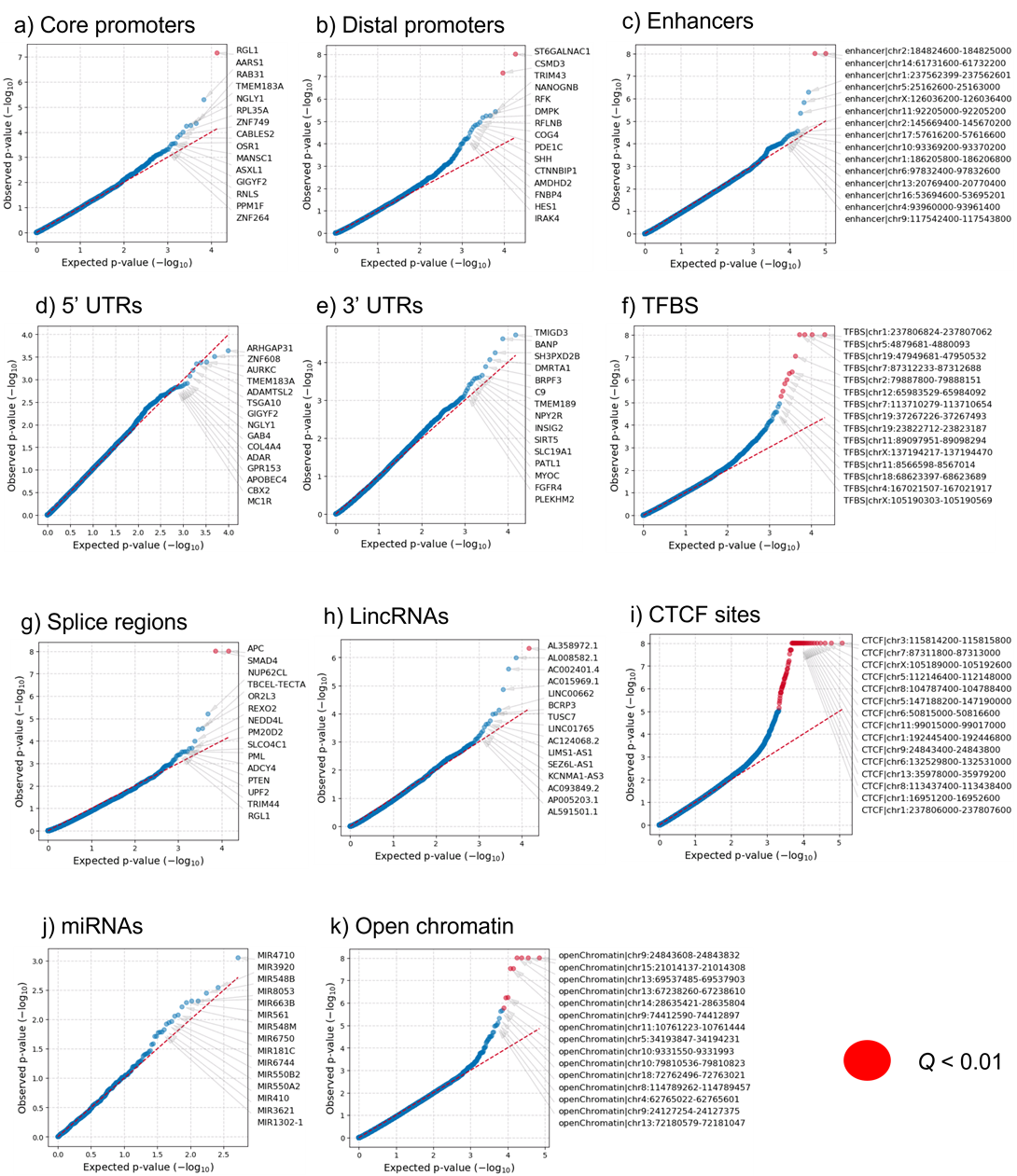


**Supplementary Figure 7. Quantile-quantile plots of OncodriveFML results for non-coding elements in MSS primary tumours**. Red circles indicate non-coding elements with *Q*<0.01. The overdispersion of the test statistic for driver status revealed by the QQ plots suggests numerous non-coding drivers. However, we remain cautious about declaring driver status for any specific gene or element based on genomic data alone. For example, analysis of the *CSMD3* distal promoter in MSS primary samples revealed two recurrently mutated positions at chr8:113437866 (n=7, 0.5%) and chr8:113437869 (n=11, 0.7%). The chr8:113437866 hotspot exclusively comprised by GTT>GGT transversions. The chr8:113437869 hotspot was characterised by CTT>CGT (n=5), CTT>CCT (n=4) and CTT>CAT (n=2) substitutions. Each was respectively located 3bp and 1bp immediately upstream of a CTCF binding motif predicted by the JASPAR database ^33^ and supported by CTCF ChIP data in colon ^34^ . This is consistent with the significant functional impact signals found at the CTCF binding site overlapping the hotspots (chr8:113437400-113438400). In line with previous observations, five of 12 CTT>CGY and three of four CTT>CCT mutations were attributable to SBS17b and SBS17a, respectively ^35,36^ . CTCF binding sites are known to be enriched in mutations, creating mutation clusters and hotspots ^37^. Based on this, we cannot exclude the possibility that these two hotspots in the *CSMD3* distal promoter are passenger mutations.

**Supplementary Figure 8**: **Structural variant (SV) signature extraction***. (a) Multimodal SV size and replication timing distributions.* Dashed lines represent thresholds used to categorize variants for signature extraction. *(b) To assess stability of SV signature extraction using the hierarchical Dirichlet process (HDP), the cohort was randomly split into halves and signatures extracted independently from each.* Nine signatures extracted from the cohort halves showed high similarity between halves (red and blue grey; cosine similarity >0.9) and high similarity with signatures extracted from the full cohort and were therefore included in subsequent analyses. MSS: microsatellite stable; MSI: microsatellite unstable.

**(a)**

CNV48A


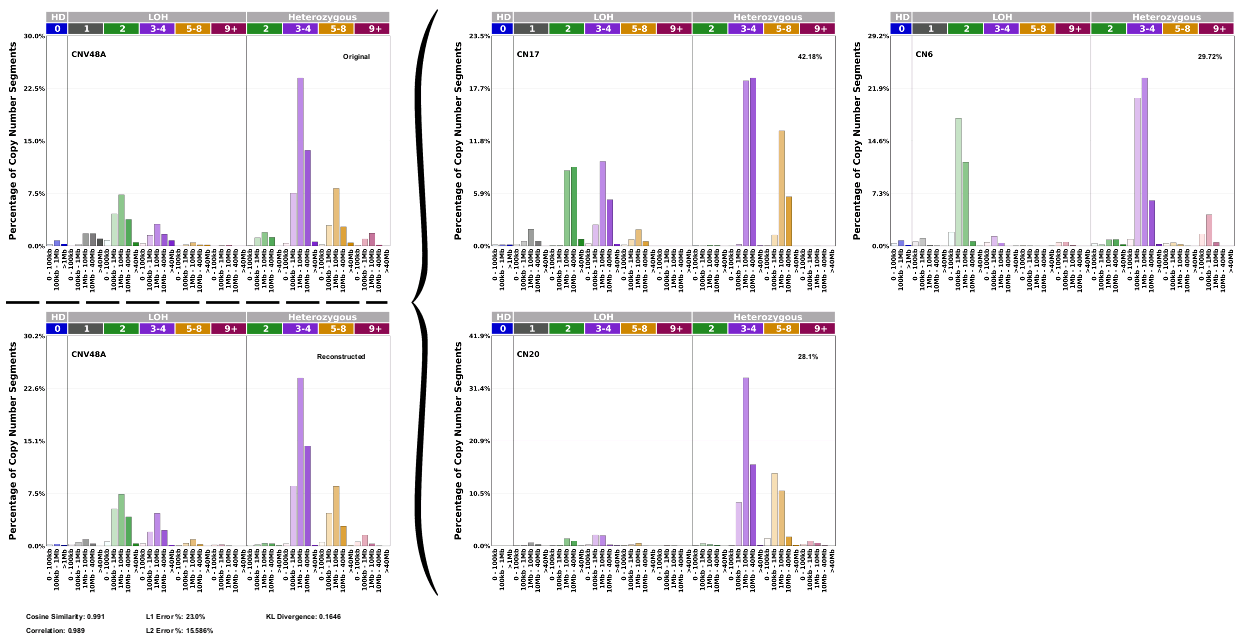


CNV48B


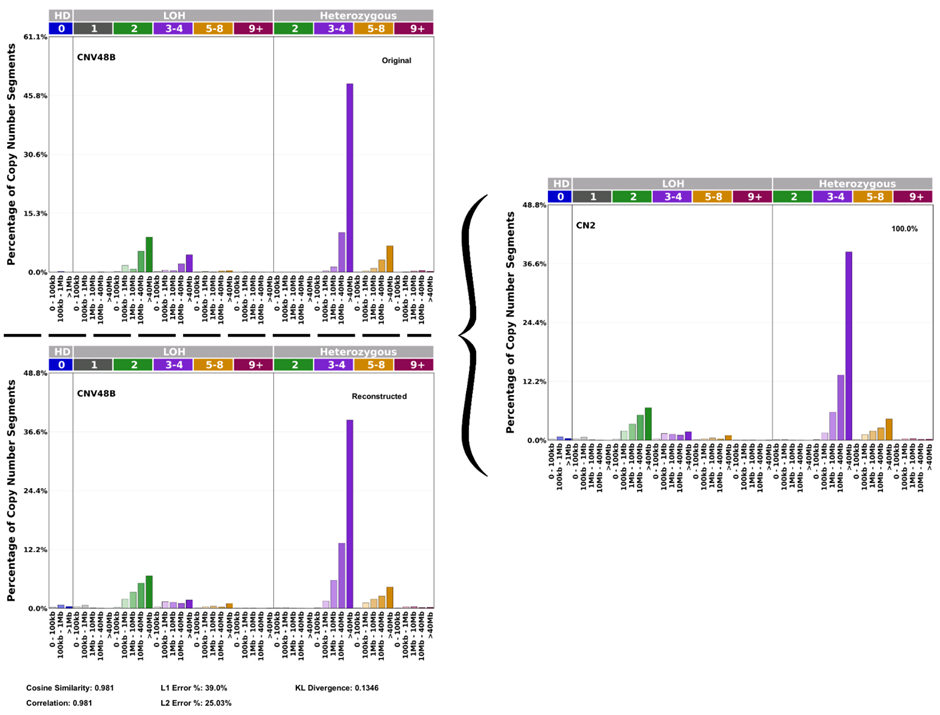


CNV48C


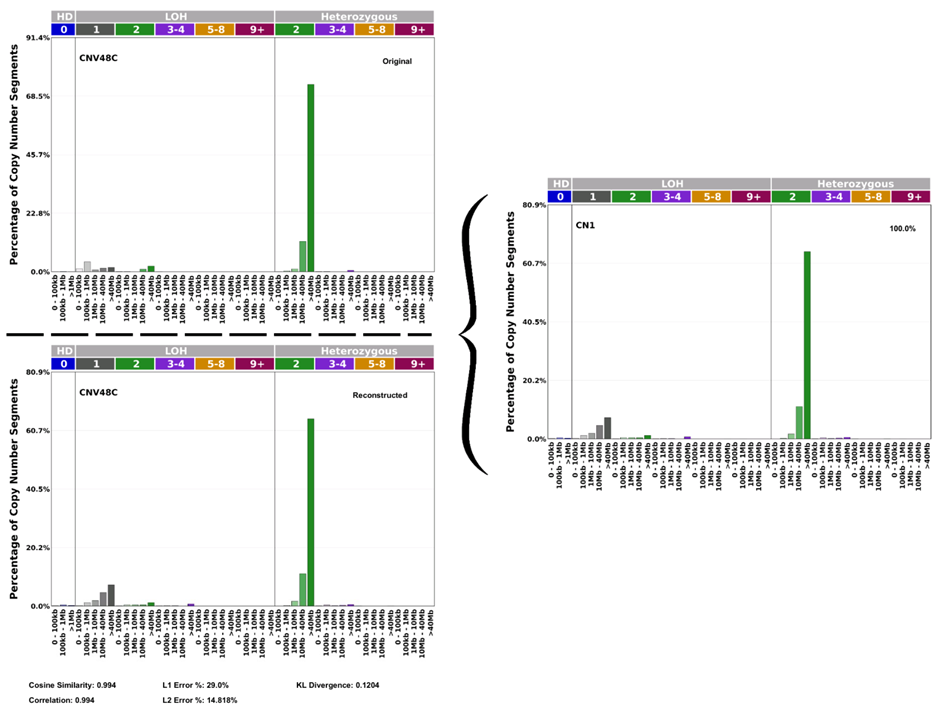


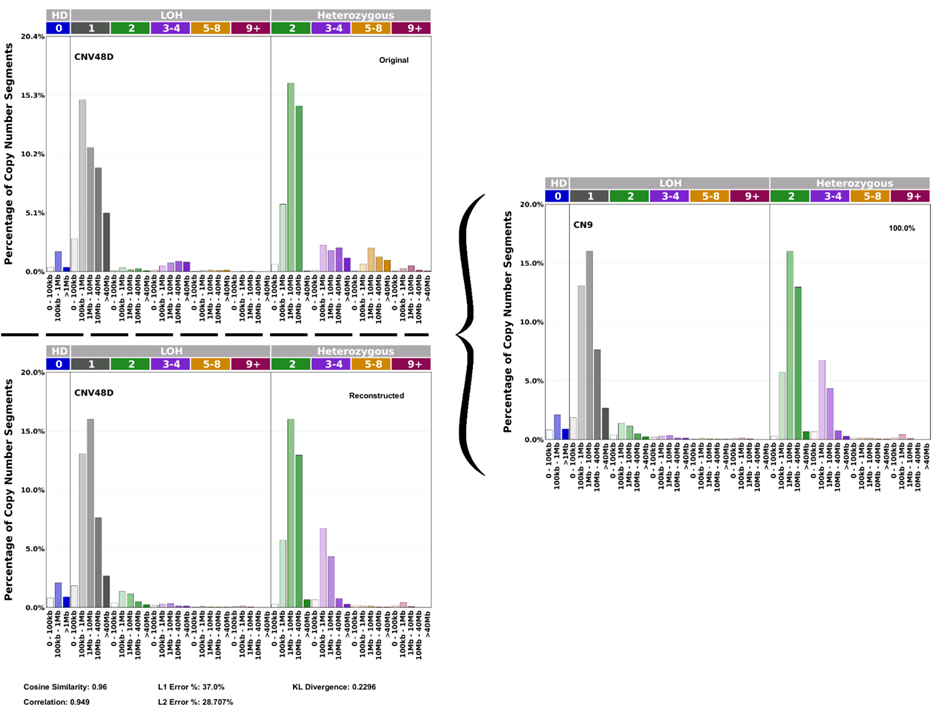
CNV48D


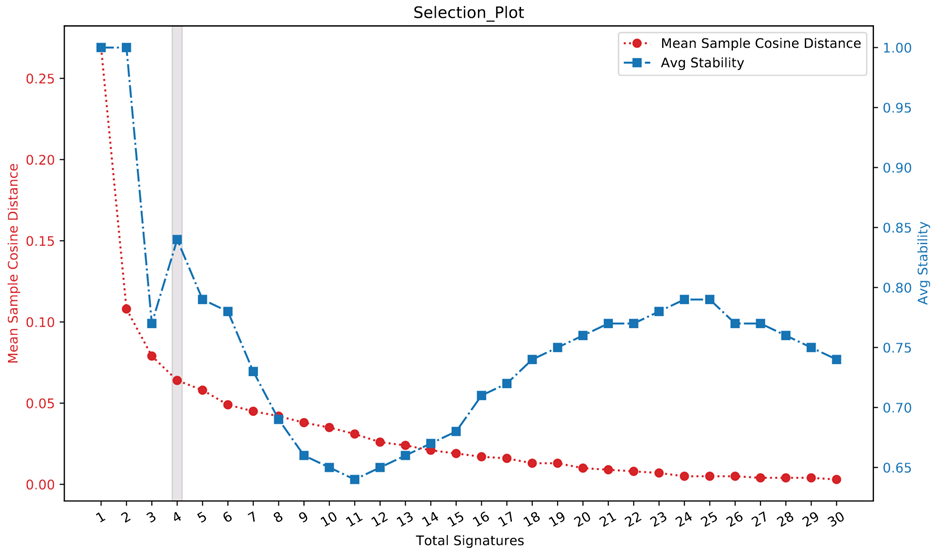
**(b)**

**Supplementary Figure 9. CN signatures.** *(a)* *Deconvolution of the four de novo signatures using the 21 COSMIC CNV signatures, to identify six contributing COSMIC signatures.* CNV48A is a heterogeneous signature, dominated by heterozygous segments of 3-8 copies. It is decomposed in to three COSMIC signatures: CN17, associated with homologous recombination deficiency (HRD) and tandem duplications (42.18%); CN6, associated with chromothripsis (29/72%); and CN20, which has a currently unexplained aetiology (28.1%). CNV48B is comprised primarily of heterozygous segments of 3-4 copies with a length of >40Mb it is deconvoluted into a single cosmic signature CN2, associated with tetraploidy. CNV48C is dominated by heterozygous segments with a copy number (CN) of 2 and is decomposed to CN1, indicative of a diploid state. CNV48D is dominated by LOH segments with a CN of 1 and heterozygous segments with a CN of 2 and to a lesser extent 3-4, it deconvoluted into CN9 which has previously been associated with chromosomally unstable diploid tumours. For each *de novo* CN signature on the top left of each plot the contributing COSMIC signatures are provided on the right, alongside the final refitted signature on the bottom left. *(b) Selection plot showing the mean sample cosine difference and average stability for de novo extraction of 1-30 copy number signature.* The accepted solution contained four de novo signatures.


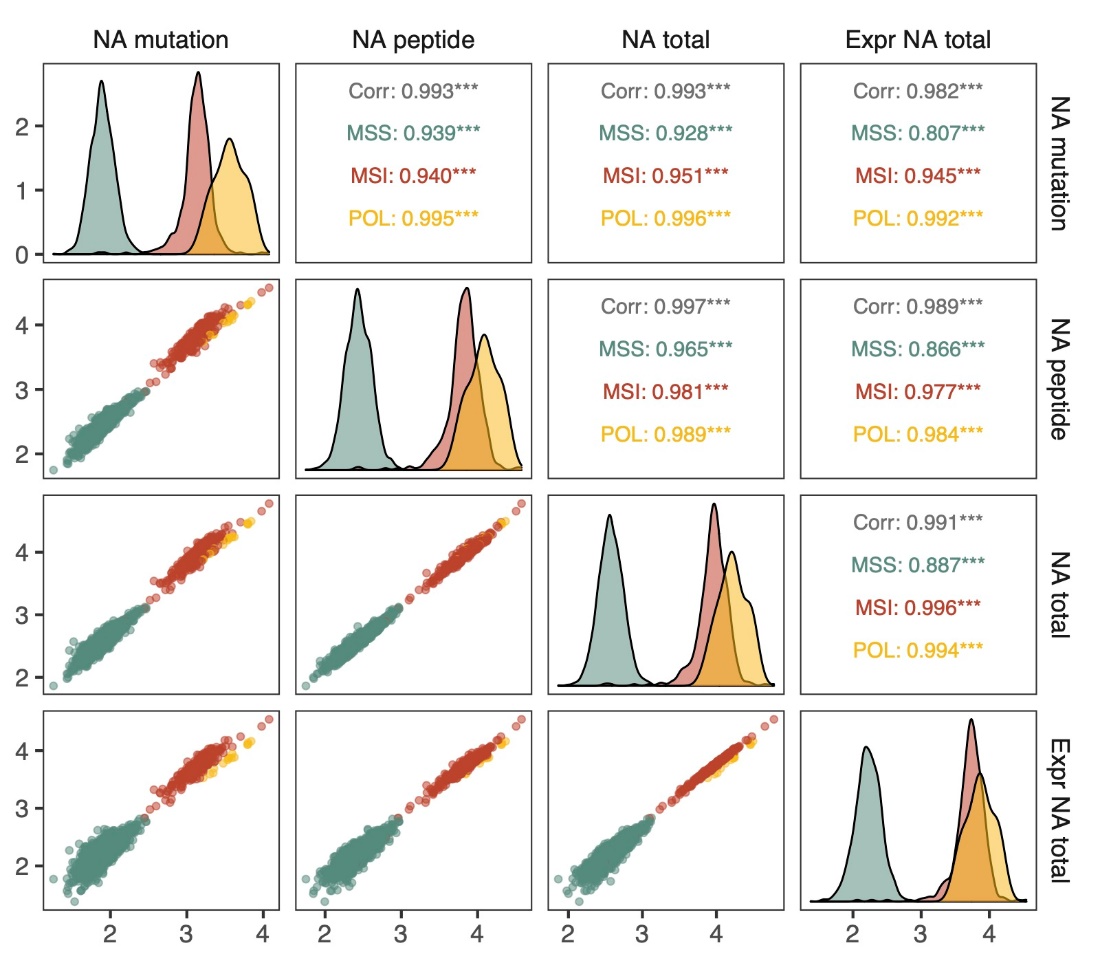


**Supplementary Figure 10. Comparison of methods for neoantigen prediction.** Lower and upper triangle and diagonal cells represent pairwise scatter plots, correlation statistics and density plots respectively (green: MSS cancers; red: MSI; yellow: POL). NA mutation: number of unique mutations giving rise to one or more neoantigens; NA peptide: number of unique neoantigen peptides; NA total: number of antigenic HLA-peptide interactions detected; Expr NA total: number of antigenic HLA-peptide interactions detected in genes expressed in ≥10% TCGA CRCs. Measures presented on log_10_ scale.

**Supplementary Figure 11. Signature activity in normal crypt epithelial cells from the right, transverse and left colon.** Where multiple crypts from the same colon region had been sampled in a single participant, the median number and proportion of variants attributed to each signature were considered. Data from Lee-Six *et al* ^38^. IDA closely resembles ID18. *P-*values were computed using two-sided Wilcoxon rank sum tests. *n*: number of participants, Trans: transverse colon.

**SUPPLEMENTARY REFERENCES**

1 Li, Y. *et al.* Patterns of somatic structural variation in human cancer genomes. *Nature* **578**, 112-121 (2020). <https://doi.org:10.1038/s41586-019-1913-9>

2 Menghi, F. *et al.* The Tandem Duplicator Phenotype Is a Prevalent Genome-Wide Cancer Configuration Driven by Distinct Gene Mutations. *Cancer cell* **34**, 197-210.e195 (2018). <https://doi.org:10.1016/j.ccell.2018.06.008>

3 Network, T. C. G. A. Comprehensive molecular characterization of human colon and rectal cancer. *Nature* **487**, 330-337 (2012). <https://doi.org:10.1038/nature11252>

4 Guo, Y. J. *et al.* ERK/MAPK signalling pathway and tumorigenesis. *Exp Ther Med* **19**, 1997-2007 (2020). <https://doi.org:10.3892/etm.2020.8454>

5 Ponting, C. P. Raf-like Ras/Rap-binding domains in RGS12- and still-life-like signalling proteins. *J Mol Med (Berl)* **77**, 695-698 (1999). <https://doi.org:10.1007/s001099900054>

6 Chakravarty, D. *et al.* OncoKB: A Precision Oncology Knowledge Base. *JCO precision oncology* **2017** (2017). <https://doi.org:10.1200/po.17.00011>

7 Shimada, K., Bachman, J. A., Muhlich, J. L. & Mitchison, T. J. shinyDepMap, a tool to identify targetable cancer genes and their functional connections from Cancer Dependency Map data. *eLife* **10** (2021). <https://doi.org:10.7554/eLife.57116>

8 Diep, C. B. *et al.* The order of genetic events associated with colorectal cancer progression inferred from meta-analysis of copy number changes. *Genes Chromosomes Cancer* **45**, 31-41 (2006). <https://doi.org:10.1002/gcc.20261>

9 Christensen, S. *et al.* 5-Fluorouracil treatment induces characteristic T>G mutations in human cancer. *Nature communications* **10**, 4571 (2019). <https://doi.org:10.1038/s41467-019-12594-8>

10 Macartney-Coxson, D. P. *et al.* Metastatic susceptibility locus, an 8p hot-spot for tumour progression disrupted in colorectal liver metastases: 13 candidate genes examined at the DNA, mRNA and protein level. *BMC cancer* **8**, 187 (2008). <https://doi.org:10.1186/1471-2407-8-187>

11 Sottoriva, A. *et al.* A Big Bang model of human colorectal tumor growth. *Nature genetics* **47**, 209-216 (2015). <https://doi.org:10.1038/ng.3214>

12 Gerstung, M. *et al.* The evolutionary history of 2,658 cancers. *Nature* **578**, 122-128 (2020). <https://doi.org:10.1038/s41586-019-1907-7>

13 Cross, W. *et al.* The evolutionary landscape of colorectal tumorigenesis. *Nature ecology & evolution* **2**, 1661-1672 (2018). <https://doi.org:10.1038/s41559-018-0642-z>

14 Arnold, A. *et al.* The majority of β-catenin mutations in colorectal cancer is homozygous. *BMC cancer* **20**, 1038 (2020). <https://doi.org:10.1186/s12885-020-07537-2>

15 Ahadova, A. *et al.* A "Two-in-One Hit" Model of Shortcut Carcinogenesis in MLH1 Lynch Syndrome Carriers. *Gastroenterology* **165**, 267-270.e264 (2023). <https://doi.org:10.1053/j.gastro.2023.03.007>

16 Davies, H. *et al.* HRDetect is a predictor of BRCA1 and BRCA2 deficiency based on mutational signatures. *Nature medicine* **23**, 517-525 (2017). <https://doi.org:10.1038/nm.4292>

17 Behjati, S. *et al.* Mutational signatures of ionizing radiation in second malignancies. *Nature communications* **7**, 12605 (2016). <https://doi.org:10.1038/ncomms12605>

18 Pich, O. *et al.* The mutational footprints of cancer therapies. *Nature genetics* **51**, 1732-1740 (2019). <https://doi.org:10.1038/s41588-019-0525-5>

19 Kucab, J. E. *et al.* A Compendium of Mutational Signatures of Environmental Agents. *Cell* **177**, 821-836.e816 (2019). <https://doi.org:10.1016/j.cell.2019.03.001>

20 Wallis, C. J. *et al.* Second malignancies after radiotherapy for prostate cancer: systematic review and meta-analysis. *BMJ (Clinical research ed.)* **352**, i851 (2016). <https://doi.org:10.1136/bmj.i851>

21 Mularoni, L., Sabarinathan, R., Deu-Pons, J., Gonzalez-Perez, A. & López-Bigas, N. OncodriveFML: a general framework to identify coding and non-coding regions with cancer driver mutations. *Genome biology* **17**, 128 (2016). <https://doi.org:10.1186/s13059-016-0994-0>

22 Ba, M. C. *et al.* Long noncoding RNA LINC00673 epigenetically suppresses KLF4 by interacting with EZH2 and DNMT1 in gastric cancer. *Oncotarget* **8**, 95542-95553 (2017). <https://doi.org:10.18632/oncotarget.20980>

23 Huang, M. *et al.* Long Noncoding RNA LINC00673 Is Activated by SP1 and Exerts Oncogenic Properties by Interacting with LSD1 and EZH2 in Gastric Cancer. *Mol Ther* **25**, 1014-1026 (2017). <https://doi.org:10.1016/j.ymthe.2017.01.017>

24 Zheng, J. *et al.* Pancreatic cancer risk variant in LINC00673 creates a miR-1231 binding site and interferes with PTPN11 degradation. *Nature genetics* **48**, 747-757 (2016). <https://doi.org:10.1038/ng.3568>

25 Fernandez-Rozadilla, C. *et al.* Deciphering colorectal cancer genetics through multi-omic analysis of 100,204 cases and 154,587 controls of European and east Asian ancestries. *Nature genetics* **55**, 89-99 (2023). <https://doi.org:10.1038/s41588-022-01222-9>

26 Cocco, E. *et al.* Colorectal Carcinomas Containing Hypermethylated MLH1 Promoter and Wild-Type BRAF/KRAS Are Enriched for Targetable Kinase Fusions. *Cancer research* **79**, 1047-1053 (2019). <https://doi.org:10.1158/0008-5472.Can-18-3126>

27 Bullman, S. *et al.* Analysis of Fusobacterium persistence and antibiotic response in colorectal cancer. *Science (New York, N.Y.)* **358**, 1443-1448 (2017). <https://doi.org:10.1126/science.aal5240>

28 Ciriello, G. *et al.* Emerging landscape of oncogenic signatures across human cancers. *Nature genetics* **45**, 1127-1133 (2013). <https://doi.org:10.1038/ng.2762>

29 Liu, Y. *et al.* Comparative Molecular Analysis of Gastrointestinal Adenocarcinomas. *Cancer cell* **33**, 721-735.e728 (2018). <https://doi.org:10.1016/j.ccell.2018.03.010>

30 Giannakis, M. *et al.* Genomic Correlates of Immune-Cell Infiltrates in Colorectal Carcinoma. *Cell reports* **15**, 857-865 (2016). <https://doi.org:10.1016/j.celrep.2016.03.075>

31 Grasso, C. S. *et al.* Genetic Mechanisms of Immune Evasion in Colorectal Cancer. *Cancer discovery* **8**, 730-749 (2018). <https://doi.org:10.1158/2159-8290.Cd-17-1327>

32 Seshagiri, S. *et al.* Recurrent R-spondin fusions in colon cancer. *Nature* **488**, 660-664 (2012). <https://doi.org:10.1038/nature11282>

33 Castro-Mondragon, J. A. *et al.* JASPAR 2022: the 9th release of the open-access database of transcription factor binding profiles. *Nucleic acids research* **50**, D165-d173 (2022). <https://doi.org:10.1093/nar/gkab1113>

34 Hammal, F., de Langen, P., Bergon, A., Lopez, F. & Ballester, B. ReMap 2022: a database of Human, Mouse, Drosophila and Arabidopsis regulatory regions from an integrative analysis of DNA-binding sequencing experiments. *Nucleic acids research* **50**, D316-d325 (2022). <https://doi.org:10.1093/nar/gkab996>

35 Guo, Y. A. *et al.* Mutation hotspots at CTCF binding sites coupled to chromosomal instability in gastrointestinal cancers. *Nature communications* **9**, 1520 (2018). <https://doi.org:10.1038/s41467-018-03828-2>

36 Katainen, R. *et al.* CTCF/cohesin-binding sites are frequently mutated in cancer. *Nature genetics* **47**, 818-821 (2015). <https://doi.org:10.1038/ng.3335>

37 Arnedo-Pac, C., Muinos, F., Gonzalez-Perez, A. & Lopez-Bigas, N. Hotspot propensity across mutational processes. *Molecular systems biology* **20**, 6-27 (2022). <https://doi.org:https://doi.org/10.1038/s44320-023-00001-w>

38 Lee-Six, H. *et al.* The landscape of somatic mutation in normal colorectal epithelial cells. *Nature* **574**, 532-537 (2019). <https://doi.org:10.1038/s41586-019-1672-7>
